# Powered vs. manual toothbrushes in fixed orthodontic patients: a systematic review and meta-analysis

**DOI:** 10.1093/ejo/cjag026

**Published:** 2026-06-11

**Authors:** Yinli Liu, Fawn Nitanee Van der Weijden, Nadine Jaquet, Godefridus August Van der Weijden, Dagmar Else Slot

**Affiliations:** Department of Orthodontics, Academic Centre for Dentistry Amsterdam (ACTA), University of Amsterdam and Vrije Universiteit Amsterdam, Gustav Mahlerlaan 3004, Amsterdam 1081 LA, The Netherlands; Department of Orthodontics, Academic Centre for Dentistry Amsterdam (ACTA), University of Amsterdam and Vrije Universiteit Amsterdam, Gustav Mahlerlaan 3004, Amsterdam 1081 LA, The Netherlands; Department of Periodontology, Academic Centre for Dentistry Amsterdam (ACTA), University of Amsterdam and Vrije Universiteit Amsterdam, Gustav Mahlerlaan 3004, Amsterdam 1081 LA, The Netherlands; Department of Periodontology, Academic Centre for Dentistry Amsterdam (ACTA), University of Amsterdam and Vrije Universiteit Amsterdam, Gustav Mahlerlaan 3004, Amsterdam 1081 LA, The Netherlands; Department of Periodontology, Academic Centre for Dentistry Amsterdam (ACTA), University of Amsterdam and Vrije Universiteit Amsterdam, Gustav Mahlerlaan 3004, Amsterdam 1081 LA, The Netherlands

**Keywords:** dental plaque, gingivitis, powered toothbrushes, orthodontic patients with fixed appliances, systematic review

## Abstract

**Background:**

The benefits of powered toothbrushes (PTBs) over manual toothbrushes (MTBs) are well established. However, for orthodontic patients with fixed appliances (FAs), the evidence remains diverging. Moreover, direct comparisons between PTBs with different action modes and MTBs in terms of plaque and gingivitis control are limited. With new data emerging, an updated synthesis of the literature, including subgroup analysis by PTB action mode, is warranted.

**Objectives:**

To determine the efficacy of PTBs vs. MTBs in orthodontic patients with FAs on reducing plaque scores (PS), gingivitis scores (GS) and gingival bleeding scores (BS).

**Search Methods:**

PubMed-MEDLINE and Cochrane-CENTRAL were searched until November 2025, and the reference lists of eligible studies were manually reviewed.

**Selection Criteria:**

Controlled clinical trials (CCTs) or randomized controlled clinical trials (RCTs); involving orthodontic patients with buccal FAs, toothbrushing performed by participants in good general health; comparing PTBs and MTBs; reporting PS, GS, and BS.

**Data Collection and Analysis:**

From the included papers, data of interest were extracted, risk of bias was evaluated by RoB 2 or ROBINS-I, and a descriptive analysis was performed. Where possible, meta-analyses were performed with subgroup meta-analyses by evaluation index or/and PTB action mode, along with heterogeneity evaluation, publication bias assessment, sensitivity analysis and trial sequential analysis. The evidence quality and effect sizes were assessed, and, where appropriate, the strength of recommendations was rated.

**Results:**

Twenty-three papers (including 39 comparisons) were eligible, with low-to-high risk of bias and considerable heterogeneity. Descriptive analysis found no overall difference in most comparisons for PS (64%), GS (76%), and BS (75%). Incremental-difference meta-analyses indicated PTBs had medium effects over MTBs on PS [standardized mean difference (SMD) = −0.77, 95% confidence interval (CI): −1.33 to −0.21, *P* = .007, *I*^2^ = 92%], large effects on GS (SMD = −0.82, 95% CI: −1.55 to −0.09, *P* = .03, *I*^2^ = 93%), and medium effects on BS (SMD = −0.51, 95% CI: −0.88 to −0.13, *P* = .008, *I*^2^ = 80%). All sensitivity analyses confirmed PTB benefits for BS, with RCT-restricted analyses supporting improvements for PS and GS. Publication bias could not be excluded for any incremental-difference meta-analyses, and evidence quality was low across all parameters. In subgroup meta-analyses by PTB action mode, counter-rotational PTBs (CR-PTBs) showed large effects on PS (very low-quality evidence).

**Conclusion:**

For fixed orthodontic patients, low-quality evidence limited by methodological weaknesses and inconsistency suggests PTBs may moderately improve PS compared with MTBs, with medium to large effects on gingival parameters. Subanalysis by action-mode showed that only CR-PTBs provided large improvements in PS over MTBs, supported by very low-quality evidence. The findings observed in this systematic review are sensitive to a limited number of studies and should be interpreted cautiously, as true effects remain uncertain and may change with more robust randomized evidence.

**Registration:**

PROSPERO (CRD42023480974).

## Introduction

Orthodontic treatment is widely recognized not only for improving dental function and facial aesthetics but also for promoting psychological well-being, including increased confidence and self-esteem [1]. However, fixed appliances (FAs) during this treatment pose challenges to achieve these benefits. Given that FAs, consisting of brackets, bands, wires, and other attachments, introduce additional surfaces, they facilitate dental plaque accumulation and impede oral hygiene procedures [2–4]. Dental plaque is a sticky biofilm that adheres to teeth and substantially contributes to the onset and development of caries and gingivitis [5]. Without proper control, long-standing, reversible gingivitis can lead to periodontal attachment loss and thereby progress to irreversible periodontitis [6]. Moreover, orthodontic tooth movement can shift supragingival plaque into a subgingival location, potentially increasing the risk of periodontitis [7]. If left untreated, periodontitis can worsen during tooth movement, ultimately resulting in unexpected and unstable orthodontic treatment outcomes [8]. Therefore, an effective oral hygiene program must be integral to fixed orthodontic treatment to reduce dental plaque and maintain good periodontal health [9].

Toothbrushing is an effective method for reducing dental plaque and is highly recommended as an essential homecare practice by oral hygiene professionals [10]. Since the efficacy of oral hygiene practices is influenced by various factors such as dexterity, motivation, and knowledge [11], powered toothbrushes (PTBs) have been introduced, demonstrating a reduced reliance on manual skills, improved patient motivation and enhanced compliance [12]. Despite this, concerns remain about the effectiveness of PTBs compared with traditional manual toothbrushes (MTBs) in plaque removal and overall oral health maintenance. Recently, several systematic reviews (SRs) have compared the effects of PTBs and MTBs in the non-orthodontic population, reaching a consensus that PTBs offer a benefit over MTBs in reducing plaque and controlling gingivitis [10, 13, 14].

For orthodontic patients with FAs, four relevant SRs have reported conflicting findings on the efficacy of PTBs vs. MTBs. Two SRs demonstrated greater effects of PTBs than MTBs [15, 16] ; one found no difference between PTBs and MTBs[17]; and the other reported insufficient evidence to draw a conclusion [18]. In addition, the most recent update of these SRs was in 2021, and since then, several new clinical trials on this topic have been conducted and published. Furthermore, owing to the previous lack of sufficient clinical trials, a direct comparison between PTBs with different action modes and MTBs in terms of plaque and gingivitis control has not been fully explored. Taking all these factors into account, there is a clear rationale for conducting a more comprehensive SR.

The present SR therefore aimed to synthesize both previously and recently published scientific papers comparing the efficacy of PTBs and MTBs on plaque scores (PS), gingivitis scores (GS), and gingival bleeding scores (BS) in orthodontic patients with FAs. Where feasible, a subgroup analysis of PTBs based on their action modes was performed.

## Materials and methods

The present SR was conducted in accordance with the Cochrane Handbook for Systematic Reviews of Interventions [19] and the Preferred Reporting Items for Systematic reviews and Meta-Analyses (PRISMA) guidelines [20]. The review protocol was established “a priori” after an initial discussion among research team members and was registered at PROSPERO (International Prospective Register of Systematic Reviews) under the number CRD42023480974 [21].

### Focused question

Based on the PICOS strategy, a focused question was formulated [22]. In orthodontic patients with FAs (P), what is the efficacy of toothbrushing with a PTB (I) vs. with an MTB (C) in removing plaque and reducing parameters of gingivitis (O) based on (randomized) controlled clinical trials (S)?

### Search strategy

To retrieve all relevant papers, a structured search strategy was employed. Two independent reviewers (Y.L. and N.J.) searched MEDLINE via PubMed (PubMed-MEDLINE) and the Cochrane Central Register of Controlled Trials (Cochrane-CENTRAL) until November 2025 to identify appropriate articles addressing the focused question. Additionally, the reference lists of included papers were manually searched to uncover any further relevant papers. No limitation was placed on publication date. For details regarding the search terms used, see Table 1.

**Table 1:** Search terms used for PubMed-MEDLINE.

The following strategy was used in the search:
((“Toothbrushing”[Mesh]) OR ((toothbrushing) OR toothbrush*))
**AND**
(Orthodontic* OR “Orthodontics”[Mesh])

The search strategy was customized according to the database being searched.

The asterisk (*) was used as a truncation symbol.

### Screening and selection

To select eligible papers, a two-stage approach was used in the Rayyan web application [23]. In the first stage, two independent reviewers (Y.L. and N.J.) screened the titles and abstracts of retrieved papers, categorizing them as definitely eligible, ineligible, or questionable. In the second stage, the same reviewers retrieved and thoroughly assessed the full texts of those had been categorized as definitely eligible and questionable. If the full text or adequate information for a final judgement was missing, repeated attempts were made to contact the first or corresponding author of the paper.

The inclusion criteria were specified as follows:

- Studies designed as controlled clinical trials (CCTs) or randomized controlled clinical trials (RCTs), including both parallel and crossover designs. Split-mouth studies were excluded.
- Studies involving human participants:
  - Participants with buccal FAs.
  - Toothbrushing performed by participants themselves.
  - Participants in good general health, with no systemic disorders.
- Studies comparing PTBs and MTBs, where PTBs were required to be rechargeable (those operated with replaceable battery were excluded), had motor-driven bristle motion and were equipped with only one brush head.
- Comparisons evaluated a single product rather than multi-product interventions.
- Studies reporting the following outcomes: PS, GS, and BS.
- Papers published in English.

Papers with studies were excluded if they involved the additional use of an interdental cleaning device, such as dental floss (DF), wood sticks, interdental brushes, and oral irrigators/dental water jets (DWJs), or examined single-brushing effects. Papers with studies that fulfilled all inclusion criteria were proceeded for data extraction and further analyses. Disagreements between the two reviewers regarding screening and selection were resolved by consensus or, if persistent, by the judgement of another reviewer (D.E.S.).

### Data extraction

Two independent reviewers (Y.L. and N.J.) utilized a specially designed form to collect information, including authors, publication year, study design, intervention duration, setting, oral prophylaxis, participant characteristics, toothbrush comparisons, toothbrushing regimen, toothpaste, other oral hygiene products or practices, compliance strategies, outcomes and the study's original conclusion. Data on PS, GS, and BS were extracted as means and standard deviations (SDs) at baseline, end-trial and as incremental differences. Whenever necessary and feasible, missing data were calculated from the reported information. Incremental differences in means were calculated as end-trial minus baseline, with negative values indicating clinical improvement. SDs of incremental differences (SDΔ) were obtained either directly from reported standard errors (SE × √*n*) or reconstructed from baseline (SD_B_) and end-trial (SD_E_) standard deviations using $\mathrm{SD}\Delta^{2}={\mathrm{SD}}_{B}^{2}+{\mathrm{SD}}_{E}^{2}-2r\;SD_{B}SD_{E}$, assuming a conservative within-subject correlation (*r* = 0.5) [19, 24]. For incomplete data that could not be derived, study authors were contacted by email to request the original data. The two reviewers resolved any disagreements through discussion. If unresolved, the final decision was made by another reviewer (D.E.S.).

### Methodological quality assessment

To assess the risk of bias in the included papers, two independent reviewers (Y.L. and F.N.V.d.W.) used the revised Risk of Bias (RoB 2) tool for RCTs and the Risk Of Bias In Non-randomized Studies of Interventions (ROBINS-I) tool for CCTs [19].

The RoB 2 tool consists of five domains for parallel RCTs and includes an additional domain (bias arising from period and carryover effects) for crossover RCTs (Supplementary Material S4A). A study was considered at “low risk of bias” if all domains indicate low risk; at “some concerns” if at least one domain shows concerns; and at “high risk of bias” if at least one domain indicates high risk or if multiple domains show some concerns, significantly lowering the confidence in the results [19].

The ROBINS-I tool assesses seven domains for CCTs (Supplementary Material S4B) and determines the overall risk of bias as follows: “low risk of bias” when the study is comparable to a well-performed RCT; “moderate risk of bias” when the study provides sound evidence for a non-randomized study; “serious risk of bias” when the study has some important methodological problems; “critical risk of bias” when the study is too problematic to provide any useful evidence and should not be included in any synthesis; and no information when there is insufficient data to make a judgment [19]. In case of disagreement, consensus between the two reviewers or the decision of another reviewer (D.E.S.) was decisive.

### Data analysis

#### Descriptive analysis

To summarize the original findings from the included studies, a descriptive analysis of PS, GS, and BS was conducted. Subgroup analyses by PTB action mode were performed only when at least two comparisons were available for each action mode.

#### Meta-analysis

A meta-analysis comparing the effects of PTBs and MTBs on reducing PS, GS, and BS in patients with FAs was conducted only if at least two comparisons were available. When feasible, the same approach was applied to subgroup meta-analyses based on evaluation index or/and PTB action mode. Baseline, end-trial, and incremental-difference scores were analyzed.

To carry out meta-analyses, Review Manager software (RevMan version 5.4.1) was used [25]. The overall mean difference of the means provided in the papers (MD) was applied as a summary statistic for indices measured on the same scale, and the standardized mean difference (SMD) for those based on different scales [19]. SMDs were estimated using Hedges’ *g* and statistically significant outcomes were interpreted as none (≤0.2), small (>0.2–0.5), medium (>0.5–0.8), or large (>0.8) effects[19, 26]. A “random-effects” model was used to calculate SMD, MD, and their corresponding 95% confidence intervals (CIs) [19, 27] with the presumption that it would be unlikely to obtain identical intervention effects across studies. When multi-arm designs were present, the sample size of any group at risk of double-counting was divided; when multiple indices for the same outcome or several measurements from a single appointment were available, the most appropriate single comparison was selected to avoid duplication in the meta-analysis [19].

#### Heterogeneity assessment

Heterogeneity was assessed across three main categories: methodological, clinical, and statistical. “Methodological and clinical heterogeneity” were evaluated based on aspects such as study design, intervention duration, oral prophylaxis, participant characteristics, PTB action modes, indices for evaluation, industry funding and conflicts of interest. “Statistical heterogeneity” was assessed by the *χ*^2^ test and the *I*^2^ statistic. A *χ*^2^ test yielding a *P* < .1 was considered significantly statistical heterogeneity. The *I*^2^ statistic is an approximate measure of inconsistency and interpreted as unimportant (<40%), moderate (30%–60%), substantial (50%–90%), or considerable (75%–100%) [19, 28].

#### Sensitivity analysis

To assess the stability of results and identify potential sources of heterogeneity, sensitivity analyses were conducted on the overall incremental-difference meta-analyses by omitting individual studies or comparisons and by restricting analyses to studies at low risk of bias, without industry support, RCTs, or combinations of these criteria, using the meta package in R [29].

#### Publication bias assessment

Publication bias was assessed by evaluating funnel plots for incremental-difference meta-analyses that included at least 10 studies [19]. Funnel forest asymmetry was evaluated using the linear regression test by Egger *et al*. [30], the rank correlation test by Begg and Mazumdar [31], and the non-parametric trim-and-fill method by Duval and Tweedie [32]. Additionally, a contour-enhanced funnel plot was introduced to improve the interpretation of funnel plot asymmetry [33]. All analyses were conducted with the meta package in R [29].

#### Trial sequential analysis

To control inflated Type I and II errors from repeated significance testing, trial sequential analysis (TSA) was performed [34]. The required information size (RIS) and significance and futility boundaries were calculated using the Lan-DeMets implementation of the O’Brien-Fleming α-spending function, with Type I and II error thresholds set at 5% and 20%, respectively (power = 80%). Incremental-difference meta-analyses using the same evaluation parameters were assessed with TSA Viewer version 0.9.5.10 Beta (Copenhagen Trial Unit, Copenhagen, Denmark).

### Grading the “body of evidence”

The Grading of Recommendation, Assessment, Development, and Evaluation (GRADE) approach was applied to rate the quality of evidence and, where applicable, the strength of recommendations [35, 36]. Subcategory analyses by PTB action mode were performed only when at least two studies were available. Two independent reviewers (Y.L. and F.N.V.d.W.) assessed the quality of evidence based on risk of bias, consistency of results, directness of evidence, precision, and publication bias. Evidence quality was initially assessed by study design, downgraded with these factors where appropriate, and finally classified as high, moderate, low, or very low [35, 36]. Effect size magnitudes were derived from pooled incremental-difference estimates when available. As high-quality evidence without methodological limitations is considered reliable and stable when updated with new data, recommendations were made only when such high-quality evidence was available [37]. Disagreements between the two reviewers were settled through discussion or, if necessary, by another reviewer (D.E.S.).

## Results

### Search and selection results

**Figure 1:**
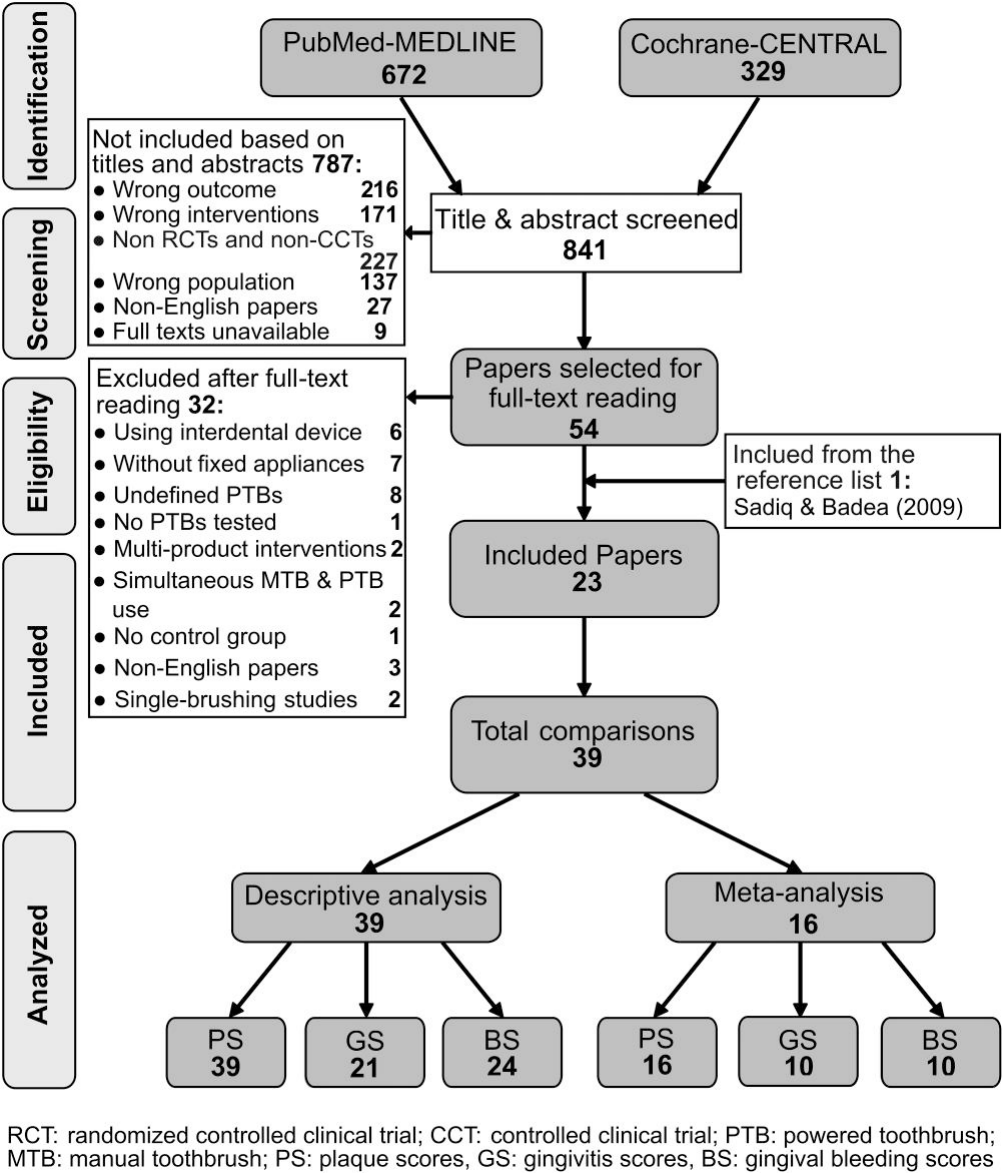
Flowchart of the study selection process for the systematic review and meta-analysis, showing identification, screening, eligibility assessment, and inclusion of studies.

The comprehensive search yielded 841 unique papers (Fig. 1). After title and abstract screening, 54 papers were advanced for full-text review and 32 were subsequently excluded for specific reasons as presented in Supplementary Material S1. Through reference list screening, an additional relevant paper was identified. In total, 23 papers [38–60], encompassing 39 comparisons, were included in the current SR. All 39 comparisons assessed PS, with 21 evaluating GS and 24 examining BS.

### Assessment of methodological and clinical heterogeneity

The 23 included papers exhibited considerable methodological and clinical heterogeneity. Supplementary Material S2 summarizes key study details, including study design, intervention duration, oral prophylaxis, participant characteristics and toothbrush types. A descriptive presentation is available in Supplementary Material S11.

#### Participant characteristics

Among the included papers, the available information indicated sample sizes ranging from 20 to 144 participants, aged 10–53 years. Specifically, nine papers focused on adolescent orthodontic patients with FAs [38, 41, 42, 44, 46, 48, 53–55]. Most papers required participants to have full-mouth FAs [38–41, 44, 47, 49–56, 58–60]. Two papers specified that participants had completed the leveling and aligning phase [38, 42]. Additional inclusion and exclusion criteria are detailed in Supplementary Material S11.

#### Clinical indices for evaluation

Significant variability in the three parameters of interest was noted among the included papers, as summarized in Supplementary Material S3. Specifically, nine clinical indices and their modifications were reported for PS, four for GS, and seven for BS, as described in Supplementary Material S11.

#### Industry funding and conflicts of interest

Industry funding also contributed to heterogeneity among the included papers. Five papers reported financial support from scientific research projects or public funds [38, 40, 42, 45, 58]. Seven disclosed financial relationships with manufacturers, including Procter & Gamble [41, 44, 54], Braun [50, 52, 54], Deprophar [54], Hospithera [54], Optiva Corporation [55], and Woog International [59]. Six papers declared no conflicts of interest [38–42, 44]. The remaining papers provided no information on funding or conflicts of interest [43, 46–49, 51, 53, 56, 57, 60].

### Methodological quality assessment

The methodological quality assessment results are shown in Supplementary Material S2, with details in Supplementary Material S4. Of the 19 RCTs, 3 were judged to have low risk of bias [39, 41, 52], 4 to have some concerns [38, 40, 42, 44], and the remaining 12 to have high risk of bias [45–51, 53–55, 57, 60]. Among the four CCTs, one were considered to have low risk of bias [59], two to have moderate risk of bias [56, 58] and the other one to have serious risk of bias [43].

### Study outcomes results

Extracted data results are detailed in Supplementary Material S5. Baseline, end-trial, and incremental-difference scores were collected or calculated by the reviewers as needed. Additional data were obtained from several first or corresponding authors after requests.

#### Descriptive analysis

Table 2 presents the descriptive summary of individual study outcomes on PS, GS, and BS, grouped by PTB action mode. Overall, most comparisons showed no significant difference between PTBs and MTBs (64% for PS, 76% for GS, and 75% for BS).

**Table 2:** A descriptive summary of the statistical significance of individual study outcomes related to the effect of toothbrushing with a PTB and an MTB on PS, GS and BS.

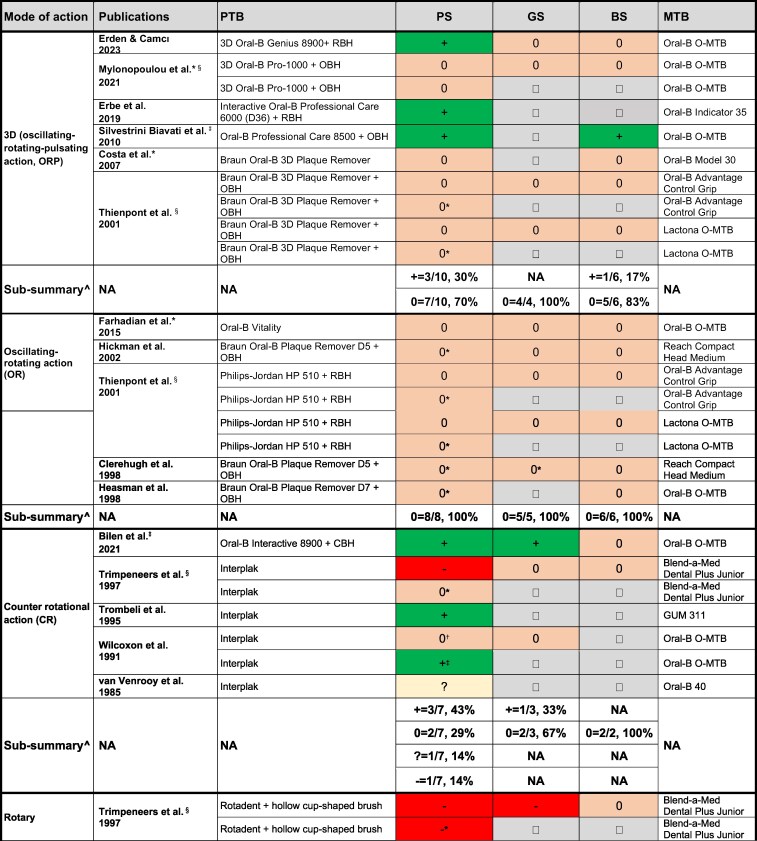
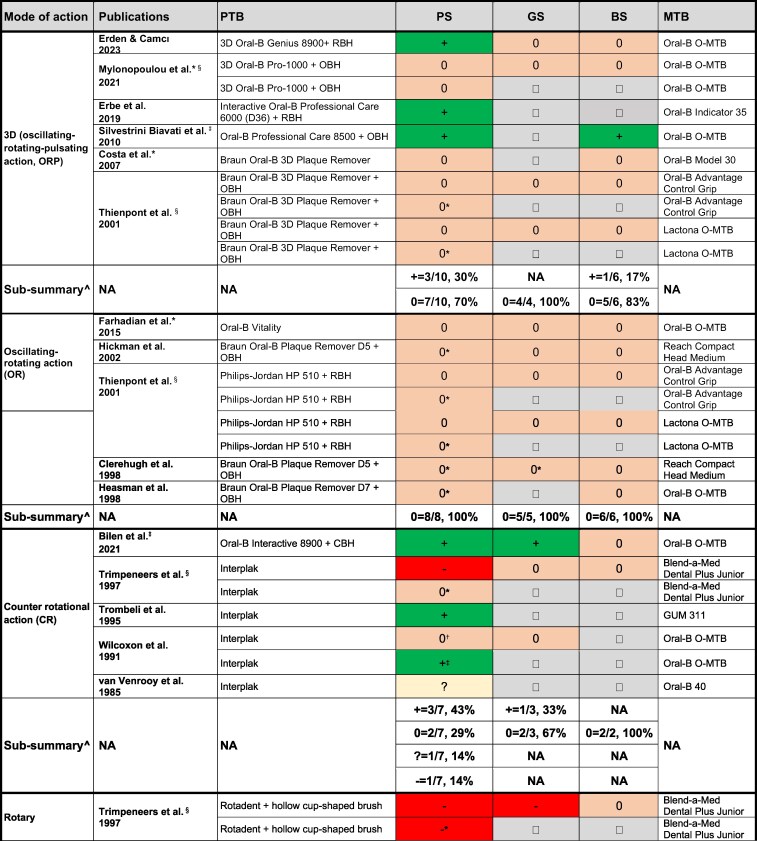
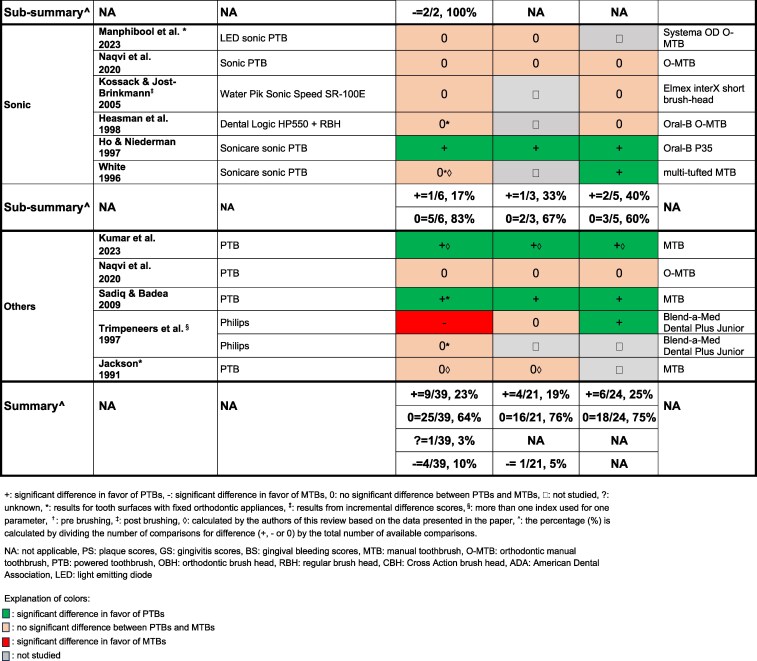

#### Meta-analysis

Overall and subgroup meta-analyses by evaluation index or/and PTB action mode were performed. Incremental-difference meta-analyses are summarized in Tables 3–6, with all forest plots shown in Supplementary Material S6.

**Table 3:** The summary of forest plots of all available studies comparing a PTB with an MTB concerning the PS, GS, and BS after the longest follow-up.

Parameters	Indices used for evaluation	No. of included studies (comparisons)	SMD	SMD effect [26]	Test overall		Test for heterogeneity		For details see the Supplementary Materials
					95% CI	*P*-value	*I* ^2^ value (%)	*P*-value	
PS^a^	QHPI, TMQH, OMPI, O’Leary PI, PI, JOPI	13 (14) [38–42, 44, 50, 52, 53, 55, 56, 58, 59]	−0.77	Medium	−1.33; −0.21	.**007**	92	**<**.**00001**	S6A-3
GS	GI, RGI	10 (10) [38–42, 50, 52, 55, 58, 59]	−0.82	Large	−1.55; −0.09	**.03**	93	**<.00001**	S6B-3
BS	BOP, MPBI, EIBI, BI, MGI-S	9 (10) [38, 39, 41, 42, 50, 52, 53, 55, 56]	−0.51	Medium	−0.88; −0.13	**.008**	80	**<.00001**	S6C-3

SMD and other data are presented for the incremental difference using a random-effects model.

*P*-values are presented in bold if *P* ≤ .05 for overall test or *P* ≤ .10 for heterogeneity test.

^a^In paper of Mylonopoulou *et al*. [41], the comparison on the orthodontic modification of the Silness and Löe plaque index was used, and in paper of Wilcoxon *et al*. [58], the postbrushing comparison was used.

PTB, powered toothbrush; MTB, manual toothbrush; SMD, standardized mean difference; QHPI, Quigley-Hein plaque index (1962) [61] and its other modifications; TMQH, the Turesky-modified Quigley–Hein index [62]; OMPI, the Orthodontic modification of the Silness and Löe plaque index (1964) [63] by Williams *et al*. (1991) [64]; O’Leary PI, O’Leary plaque index and its modification [94]; PI, plaque index by Silness and Löe (1964) [63]; VPI, visible plaque index by Ainamo and Bay (1975) [65]; HAI, hygiene analysis index; JOPI, Jackson orthodontic plaque index (1991) [59]; GI, Löe and Silness gingival index (1963, 1964, 1967) [63, 66, 67] and its modifications; RGI, Ramfjord Gingival Index (1959) [68]; BOP, bleeding on probing; MPBI, modified papillary bleeding index; EIBI, Eastman interdental bleeding index by Caton and Polson [69]; BI, gingival bleeding scores by Ainamo and Bay (1975) [65]; MGI-S, the modified simplified gingival index of Lindhe *et al*. [70].

**Table 4:** The summary of forest plots of available studies comparing a PTB with an MTB concerning the same plaque, gingivitis or gingival bleeding index after the longest follow-up.

Parameters	Indices used for evaluation	No. of included studies (comparisons)	MD	Test overall		Test for heterogeneity		For details see the Supplementary Materials
				95% CI	*P*-value	*I* ^2^ value (%)	*P*-value	
PS	OMPI (0–3)	4 (4) [41, 42, 50, 52]	−0.03	−0.11; 0.04	.39	55	.**08**	S6A-6
	QHPI, TMQH (0–5)	2 (2) [38, 44]	−0.54	−1.14; 0.06	.08	94	**<.0001**	S6A-9
	O’Leary PI^a^ (0–1)	2 (2) [41, 58]	−0.17	−0.48; 0.15	.30	96	**<.00001**	S6A-12
	PI (0–3)	3 (3) [39, 40, 55]	−0.61	−1.35; 0.12	.10	99	**<.00001**	S6A-15
	VPI (0–1)	1 (2) [53]	0.06	0.00; 0.13	**.04**	0	.42	S6A-18
GS	GI (0–3)	9 (9) [38–42, 50, 52, 55, 59]	−0.22	−0.43; −0.02	**.04**	98	**<.00001**	S6B-6
BS	BOP (0–1)	3 (3) [38, 42, 55]	−0.18	−0.46; 0.11	.22	98	**<.00001**	S6C-6
	MPBI (0–3)	2 (2) [39, 56]	−0.54	−0.71; −0.36	**<.00001**	0	.50	S6C-9
	EIBI (0–1)	2 (2) [50, 52]	−0.06	−0.14; 0.02	.12	0	.71	S6C-12
	BI (0–1)	1 (2) [53]	0.01	−0.04; 0.05	.82	0	.82	S6C-15

MD and other data are presented for the incremental difference using a random-effects model.

*P*-values are presented in bold if *P* ≤ .05 for overall test or *P* ≤ .10 for heterogeneity test.

^a^In paper of Wilcoxon *et al*. [58], the postbrushing comparison was used.

PTB, powered toothbrush; MTB, manual toothbrush; MD, mean difference; OMPI, the Orthodontic modification of the Silness and Löe plaque index (1964) [63] by Williams *et al*. (1991) [64]; QHPI, Quigley-Hein plaque index (1962) [61] and its other modifications; TMQH, the Turesky-modified Quigley–Hein index (1970) [62]; O’Leary PI, O’Leary plaque index and its modification [94]; PI, plaque index by Silness and Löe (1964) [63]; VPI, visible plaque index by Ainamo and Bay (1975) [65]; GI, Löe and Silness gingival index (1963, 1964, 1967) [63, 66, 67] and its modifications; BOP, bleeding on probing; MPBI, modified papillary bleeding index; EIBI, Eastman interdental bleeding index by Caton and Polson [69]; BI, gingival bleeding scores by Ainamo and Bay (1975) [65].

**Table 5:** The summary of forest plots of available studies comparing a PTB with an MTB concerning the PS, GS and BS based on the same action mode of PTBs after the longest follow-up.

Parameters	Action mode of PTBs	No. of included studies (comparisons)	SMD	SMD effect [26]	Test overall		Test for heterogeneity		For details see the Supplementary Materials
					95% CI	*P*-value	*I* ^2^ value (%)	*P*-value	
PS	ORP/OR^b^	6 (6) [38, 41, 44, 50, 52, 53]	−0.46	NA	−1.20; 0.28	.23	92	**<**.**00001**	S6A-21
	ORP^b^	3 (3) [38, 41, 44]	−1.13	NA	−2.74; 0.49	.17	95	**<.00001**	S6A-24
	OR	3 (3) [50, 52, 53]	0.15	NA	−0.21; 0.51	.42	46	.16	S6A-27
	CR^c^	2 (2) [42, 58]	−1.45	Large	−2.85; −0.05	**.04**	77	**.04**	S6A-30
	Sonic	4 (4) [40, 53, 55, 56]	−1.20	NA	−2.67; 0.28	.11	93	**<.00001**	S6A-33
GS	ORP/OR GI (0–3)	4 (4) [38, 41, 50, 52]	0.00^a^	NA	−0.03; 0.04	.79	0	.91	S6B-9
	ORP GI (0–3)	2 (2) [38, 41]	0.01^a^	NA	−0.03; 0.05	.79	0	.60	S6B-12
	OR GI (0–3)	2 (2) [50, 52]	0.00^a^	NA	−0.05; 0.06	.93	0	.61	S6B-15
	CR	2 (2) [42, 58]	−0.70	NA	−1.80; 0.39	.21	73	**.05**	S6B-18
	Sonic GI (0–3)	2 (2) [40, 55]	−0.30^a^	NA	−0.75; 0.15	.19	94	**<.0001**	S6B-21
BS	ORP/OR	5 (5) [38, 41, 50, 52, 53]	−0.15	NA	−0.36; 0.07	.18	0	.89	S6C-18
	ORP	2 (2) [38, 41]	−0.11	NA	−0.46; 0.25	.56	0	.72	S6C-21
	OR	3 (3) [50, 52, 53]	−0.17	NA	−0.43; 0.10	.21	0	.63	S6C-24
	Sonic	3 (3) [53, 55, 56]	−1.65	NA	−3.53; 0.22	.08	94	**<.00001**	S6C-27

SMD, MD and other data are presented for the incremental difference scores using a random-effects model.

*P*-values are presented in bold if *P* ≤ .05 for overall test or *P* ≤ .10 for heterogeneity test.

^a^MD.

^b^In paper of Mylonopoulou *et al*. [41], the comparison on the orthodontic modification of the Silness and Löe plaque index was used.

^c^In paper of Wilcoxon *et al*. [58], the postbrushing comparison was used.

PTB, powered toothbrush; MTB, manual toothbrush; SMD, standardized mean difference; MD, mean difference; NA, not applicable; PS, plaque scores; GS, gingivitis scores; BS, gingival bleeding scores; ORP, oscillating-rotating-pulsating action; OR, oscillating-rotating action; CR, counterrotational action; GI, Löe and Silness gingival index (1963, 1964, 1967) [63, 66, 67] and its modifications.

**Table 6:** The summary of forest plots of available studies comparing a PTB with an MTB concerning the PS, GS and BSbased on the same index and action mode of PTBs after the longest follow-up.

Parameters	Action mode of PTBs and index	No. of included studies (comparisons)	MD	Test overall		Test for heterogeneity		For details see the Supplementary Materials
				95% CI	*P*-value	*I* ^2^ value (%)	*P*-value	
PS	ORP/OR OMPI (0–3)	3 (3) [41, 50, 52]	0.00	−0.04; 0.04	.99	0	.57	S6A-36
	ORP QHPI, TMQH (0–5)	2 (2) [38, 44]	−0.54	−1.14; 0.06	.08	94	**<.0001**	S6A-39
	OR OMPI (0–3)	2 (2) [50, 52]	0.00	−0.11; 0.11	.98	11	.29	S6A-42
	Sonic PI (0–3)	2 (2) [40, 55]	−0.51	−1.96; 0.94	.49	99	**<.00001**	S5A-45
GS	ORP/OR GI (0–3)	4 (4) [38, 41, 50, 52]	0.00	−0.03; 0.04	.79	0	.91	S6B-24
	ORP GI(0–3)	2 (2) [38, 41]	0.01	−0.03; 0.05	.79	0	.60	S6B-27
	OR GI (0–3)	2 (2) [50, 52]	0.00	−0.05; 0.06	.93	0	.61	S6B-30
	Sonic GI (0–3)	2 (2) [40, 55]	−0.30	−0.75; 0.15	.19	94	**<.0001**	S6B-33
BS	OR EIBI (0–1)	2 (2) [50, 52]	−0.06	−0.14; 0.02	.12	0	.71	S6C-30

MD and other data are presented for the incremental difference scores using a random-effects model.

*P*-values are presented in bold if *P* ≤ .05 for overall test or P ≤ .10 for heterogeneity test.

PTB, powered toothbrush; MTB, manual toothbrush; MD, mean difference; PS, plaque scores; GS, gingivitis scores; BS, gingival bleeding scores; ORP, oscillating-rotating-pulsating action; OR, oscillating-rotating action; OMPI, the Orthodontic modification of the Silness and Löe plaque index (1964) [63] by Williams *et al*. (1991) [64]; QHPI, Quigley-Hein plaque index (1962) [61] and its modification; TMQH, the Turesky-modified Quigley–Hein index (1970) [62]; PI, plaque Index by Silness and Löe (1964) [63]; GI, Löe and Silness gingival index (1963, 1964, 1967) [63, 66, 67] and its modifications; EIBI, Eastman interdental bleeding index by Caton and Polson [69].

Overall incremental-difference meta-analyses showed that PTBs were more effective than MTBs in reducing PS (SMD = −0.77, 95% CI: −1.33 to −0.21, *P* = .007), GS (SMD = −0.82, 95% CI: −1.55 to −0.09, *P* = .03), and BS (SMD = −0.51, 95% CI: −0.88 to −0.13, *P* = .008), as shown in Table 3.

In subgroup meta-analyses by PTB action mode, counter-rotational PTBs (CR-PTBs) showed greater benefits than MTBs when PS were assessed (SMD = −1.45, 95% CI: −2.85 to −0.05, *P* = .04). Other subgroup meta-analyses by PTB action mode showed no significant differences between PTBs and MTBs across any outcome, including PS, GS, and BS (Table 5).

#### Statistical heterogeneity

All overall incremental-difference meta-analyses presented substantial to considerable heterogeneity (Table 3), and approximately half of the subgroup incremental-difference meta-analyses by evaluation index or/and PTB action mode showed moderate to considerable heterogeneity (Tables 4–6).

#### Sensitivity analysis

Sensitivity analyses are detailed in Supplementary Material S7, with results from analyses restricted to studies at low risk of bias, without industry support, RCTs, or combinations of these criteria summarized in Table 7. Sensitivity analyses were not feasible for studies meeting both low risk of bias and no industry support, or all three criteria combined. All sensitivity analyses of BS showed greater effects for PTBs than MTBs, with small-to-medium effect sizes and numerically lower heterogeneity than the primary meta-analysis. Sensitivity analyses restricted to RCTs demonstrated PTB benefits over MTBs for PS and GS, with comparable effect sizes and heterogeneity to overall analysis.

**Table 7:** A summary of sensitivity analyses of incremental-difference meta-analyses restricted to studies at low risk of bias, without industry involvement, randomized controlled trials, and their combinations.

Restriction factors	Parameters	No. of included studies (comparisons)	SMD	SMD effect [26]	Test overall		Test for heterogeneity		For details see the Supplementary Materials
					95% CI	*P*-value	*I* ^2^ value (%)	*P*-value	
Only low-risk-of-bias studies	PS	4 (4) [39, 41, 52, 59]	−0.55	NA	−1.38; 0.27	.19	92	**<**.**0001**	S7A-2
	GS	4 (4) [39, 41, 52, 59]	−0.67	NA	−1.96; 0.61	.30	97	**<.0001**	S7B-2
	BS	3 (3) [39, 41, 52]	−0.43	Small	−0.85; −0.02	**.04**	68	**.04**	S7C-2
Only studies without industry involvement	PS	5 (5) [38–40, 42, 58]	−0.89	NA	−1.80; 0.03	.06	91	**<.0001**	S7A-3
	GS	5 (5) [38–40, 42, 58]	−0.85	NA	−1.95; 0.26	.13	94	**<.0001**	S7B-3
	BS	3 (3) [38, 39, 42]	−0.55	Medium	−1.03; −0.07	**.02**	59	**.09**	S7C-3
Only RCT studies	PS	10 (11) [38–42, 44, 50, 52, 53, 55]	−0.73	Medium	−1.39; −0.06	**.03**	94	**<.0001**	S7A-4
	GS	8 (8) [38–42, 50, 52, 55]	−1.02	Large	−1.90; −0.14	**.02**	95	**<.0001**	S7B-4
	BS	8 (9) [38, 39, 41, 42, 50, 52, 53, 55]	−0.44	Small	−0.83; −0.06	**.02**	81	**<.0001**	S7C-4
Only low-risk-of-bias RCTs	PS	3 (3) [39, 41, 52]	−0.60	NA	−1.66; 0.45	.26	95	**<.0001**	S7A-5
	GS	3 (3) [39, 41, 52]	−0.83	NA	−2.48; 0.83	.33	98	**<.0001**	S7B-5
	BS	3 (3) [39, 41, 52]	−0.43	Small	−0.85; −0.02	**.04**	68	**.04**	S7C-5
Only RCTs without industry involvement	PS	4 (4) [38–40, 42]	−0.62	NA	−1.61; 0.38	.23	92	**<.0001**	S7A-6
	GS	4 (4) [38–40, 42]	−1.02	NA	−2.29; 0.25	.12	95	**<.0001**	S7B-6
	BS	4 (4) [38, 40, 42]	−0.55	Medium	−1.03; −0.07	**.02**	59	**.09**	S7C-6

*P*-values are presented in bold if *P* ≤ .05 for overall test or *P* ≤ .10 for heterogeneity test.

RCT, randomized controlled clinical trials; SMD, standardized mean difference; NA, not applicable; PS, plaque scores; GS, gingivitis scores; BS, gingival bleeding scores.

#### Publication bias

Results of publication bias assessment are presented in Supplementary Material S8. Based on funnel plot asymmetry and further statistical analyses of the overall incremental-difference meta-analyses, publication bias could not be excluded for any parameter.

#### Trial sequential analysis

Almost all TSA results showed that the accumulated number of participants did not reach the RIS, and the cumulative Z-curve did not ultimately cross the O’Brian-Fleming boundaries (Supplementary Material S9), indicating that the corresponding incremental-difference meta-analyses were inconclusive.

### Evidence profile

Following GRADE guidelines, the factors employed to evaluate the quality of evidence are outlined in Table 8 [35]. The overall quality of evidence was low for all three parameters, and therefore no recommendation was made. The estimated magnitudes of the effect size [26] were medium for PS, large for GS and medium for BS. As predetermined, the subcategories included oscillating-rotating-pulsating (ORP)/oscillating-rotating (OR), ORP, OR, CR, and sonic PTB groups. Their corresponding evidence quality and effect size magnitudes are summarized in Table 9, with further details in Supplementary Material S10A–C.

**Table 8:** Estimated evidence profile regarding the efficacy of PTBs vs. MTBs on dental plaque and gingivitis control in orthodontic patients with FAs.

Determinants of the quality	PS	GS	BS
Study design	RCT/CCT	RCT/CCT	RCT/CCT
No. of studies (Fig. 1, Table 2)	23	15	17
No. of comparisons (Fig. 1, Table 2)	39	21	24
No. of meta-analysis (Fig. 1, Table 3)	14	10	10
Risk of bias (Supplementary Materials S2, S4)	Low to high	Low to high	Low to high
Consistency	Inconsistent	Inconsistent	Inconsistent
Directness	Direct	Direct	Direct
Precision	Precise	Precise	Precise
Publication bias (Supplementary Material S8)	Possible	Possible	Possible
The quality of a body of evidence	Low	Low	Low
Magnitudes of the effect size	Medium	Large	Medium

PTB, powered toothbrush; MTB, manual toothbrush; PS, plaque scores; GS, gingivitis scores; BS, gingival bleeding scores; RCT, randomized controlled clinical trial; CCT, controlled clinical trial; FAs, fixed appliances.

**Table 9:** A summary of estimated evidence profile regarding the efficacy of PTBs with different action modes vs. MTBs on dental plaque and gingivitis control in orthodontic patients with FAs.

Parameters	Determinants	ORP/OR	ORP	OR	CR	Sonic	For details see the Supplementary Materials
PS	The quality of evidence	Low	Very low	Moderate	Very low	Low	S10A
	Magnitudes of the effect size	None	None	None	Large	None	
GS	The quality of evidence	Moderate	Low	Low	Very low	Very low	S10B
	Magnitudes of the effect size	None	None	None	None	None	
BS	The quality of evidence	Moderate	Low	Moderate	Low	Low	S10C
	Magnitudes of the effect size	None	None	None	Not available	None	

PTB, powered toothbrush; MTB, manual toothbrush; PS, plaque scores; GS, gingivitis scores; BS, gingival bleeding scores; FAs, fixed appliances; ORP, oscillating-rotating-pulsating action; OR, oscillating-rotating action; CR, counter-rotational action.

## Discussion

Orthodontic patients with FAs are at higher risk of developing caries and gingivitis due to increased plaque accumulation [3, 4]. Since toothbrushing is a key strategy for plaque control [10], determining the comparable efficacy of PTBs and MTBs is essential for managing oral health in these patients. The current SR included 23 papers with 39 comparisons evaluating the effects of PTBs vs. MTBs on plaque and gingivitis control in orthodontic patients wearing FAs. Overall, PTBs demonstrated significant reductions in PS (medium effects), GS (large effects), and BS (medium effects) compared with MTBs, with evidence rated as low quality. Subcategory analyses further revealed that CR-PTBs had large effects over MTBs on PS (very low-quality evidence).

### In the light of previous SRs

The present SR indicated that PTBs were more effective than MTBs in reducing dental plaque and gingivitis in orthodontic patients with FAs, consistent with two earlier SRs [15, 16]. However, two other earlier SRs reported “no difference” [17] or “insufficient evidence” to conclude [18]. These discrepancies likely stem from variations in the included studies based on differing inclusion criteria across SRs.

The present SR excluded three papers evaluating replaceable battery-operated PTBs [71–73], two of which [72, 73] were included in three previous SRs [15–17]. As rechargeable PTBs showed greater PS reduction than replaceable battery-operated ones [74], targeting the synthesis on rechargeable models can reflect the most likely effects of PTBs. PTBs were additionally restricted to those with motor-driven bristle motion, resulting in the exclusion of three papers [75–77]. This restriction maintained a focused comparison on the mechanical plaque-removal effects of PTBs vs. MTBs, supported by the literature [74, 78]. The current SR also excluded two papers that assessed PTBs with multiple functional brush heads per use [79, 80]. This is consistent with the established definition of PTBs, which states that the brush has a single functional brush head [74]. Furthermore, the present SR, along with two previous SRs [15, 18], excluded studies where participants used interdental cleaning devices, such as DWJs and DF, while the other two earlier SRs permitted studies with their additional use [16, 17]. Since adding DWJs or DF to regular toothbrushing significantly improves plaque and gingival condition in orthodontic patients with FAs [81, 82], excluding such studies helps avoid confounding effect in PTB vs. MTB comparisons.

Regarding study design, the present SR and one earlier SR included both RCTs and CCTs [16], while the other three earlier SRs included only RCTs [15, 17, 18]. Although RCTs have long been considered the gold standard for evaluating effectiveness [83], including CCTs can ensure comprehensive coverage of relevant clinical trials [19]. In addition, the current SR and three previous SRs included crossover trials [15, 16, 18], whereas one did not [17]. The Cochrane Handbook confirmed that crossover trials are suitable for evaluating interventions with temporary effects in treating stable, chronic conditions [19]. Furthermore, the split-mouth design was excluded by all SRs, as it poorly represents the real-world toothbrushing practice and differ analytically from parallel-group studies, increasing heterogeneity [84].

Unlike three previous SRs requiring a minimum 4-week intervention duration [15, 17, 18], this SR and one other did not set such restrictions [16]. Short-term studies (≤4 weeks) are useful for assessing immediate plaque reduction while controlling confounders like subject compliance [85]. In addition, GS can typically respond to plaque control within 3 weeks [85]. Therefore, despite the ADA Seal of Acceptance guidelines suggesting a 30-day evaluation period for gingivitis management, the present SR included both short- and long-term trials to comprehensively evaluate toothbrush efficacy on plaque removal and gingivitis control, as advocated in earlier work [86].

The present SR included only papers published in English, while two previous SRs included an unpublished dissertation [87] and imposed no language limitation [15, 18]. The Cochrane Handbook suggests including all publication types in any language to reduce bias [19], but research has also indicated that non-English and unpublished studies represented a small proportion of included studies and rarely affected the review results [88]. Accessing these sources is also challenging due to language barriers and reliance on authors’ cooperation to share their unpublished results [89]. Thus, restricting the inclusion to published, English-language papers was considered a justified approach.

### PTBs with different action modes

Alongside overall analyses, subcategory analyses were conducted to assess the effectiveness of PTBs with different action modes against MTBs. Given that ORP- and OR-PTBs share oscillating and rotating movements, they were also analyzed collectively.

ORP-PTBs, building upon OR-PTBs, are suggested by manufacturers as more advanced with enhanced cleaning efficacy, particularly in hard-to-reach approximal sites [90]. In non-orthodontic individuals, ORP-PTBs offer advantages over MTBs in removing plaque and improving gingival health [90]. However, among orthodontic patients with FAs, the present findings indicated no clear advantage of ORP-PTBs compared with MTBs for any parameter. This is likely because orthodontic patients with FAs tend to accumulate more plaque than non-orthodontic individuals, and this plaque is more difficult to remove due to the hard-to-reach areas created by FAs [2–4]. Thus, a possible explanation for the discrepancy in effectiveness of ORP-PTB vs. MTBs between non-orthodontic and orthodontic populations is that orthodontic patients with FAs may retain sufficient plaque to sustain gingivitis, regardless of the toothbrush used. Similarly, the present SR also identified no significant differences between OR-PTBs and MTBs for any evaluated parameter for orthodontic patients with FAs, consistent with a previous SR examining short-term gingivitis outcomes [15].

CR-PTBs produced a greater reduction in PS in orthodontic patients with FAs than MTBs, but no significant differences were observed for GS or BS. Although the current findings may appear to conflict with the well-established relationship between plaque and gingivitis in other populations, the findings are compatible when considering that the meta-analysis measured incremental changes in plaque rather than absolute residual plaque, which drives the initiation and progression of gingivitis [5]. Therefore, even if CR-PTBs achieved a statistically significant reduction compared with MTBs in PS, orthodontic patients with FAs may still retain enough plaque to sustain gingival inflammation. Additional factors, such as alterations in subgingival microbial composition favoring periodontopathic bacteria [91] and mechanical irritation caused by FAs [92], may further maintain gingivitis. Moreover, these findings are based on only two comparisons, which had moderate to high risk of bias and substantial heterogeneity; consequently, the quality of evidence was rated as very low and should be interpreted cautiously (Table 9, Supplementary Material S10A).

### Discrepancy between descriptive and meta-analytic results

Some discrepancies between descriptive and meta-analytic results can be found in Tables 2 and 3. While overall descriptive analyses showed no significant differences between PTBs and MTBs in most comparisons across all three parameters, the corresponding meta-analyses demonstrated medium to large effects of PTBs over MTBs. These divergences arise because the meta-analyses included fewer comparisons, particularly missing non-significant PTB-MTB comparisons from the descriptive analyses.

The discrepancy in the number of included comparisons between the descriptive and meta-analysis arises from their fundamental methodological differences. In the current SR, the descriptive analysis was performed with conventional vote counting method. Since only minimal statistical information is required (e.g. direction of effect with *P*-value), it can accommodate studies with incomplete outcome reporting (a common barrier to meta-analysis) [19]. However, vote counting does not quantify effect size magnitudes, cannot account for differences in the relative sizes of studies, and fails to maintain statistical power with increasing study numbers unless studies are large and intervention effects are at least moderate [19]. Meta-analysis, as a more robust statistical approach, can address these limitations [19]. Nevertheless, certain comparisons from the descriptive analyses were unfit for the meta-analyses due to missing necessary data, which aligns with the indication of possible publication bias. Furthermore, substantial to considerable heterogeneity was observed for overall incremental-difference meta-analyses (Table 3) and methodological limitations may have also influenced the results (Table 7). To reinforce the rigor of the evidence base, these factors were incorporated into the evidence grading, leading to downgraded quality assessments for meta-analytic findings.

### Heterogeneity

According to the Cochrane Handbook and GRADE guidelines [19, 28] , heterogeneity among the included studies was statistically tested (Table 3–6).

The numerically lower level of heterogeneity in the subgroup meta-analyses by evaluation index suggests that the diversity of indices used across the included studies may contribute to the substantial to considerable heterogeneity found in the overall meta-analyses. Researchers utilized nine different indices for PS, four for GS, and seven for BS. Evidence indicates that plaque indices are not interchangeable for quantifying plaque during orthodontic therapy [93]. Therefore, to enhance consistency in further SRs on orthodontic patients with FAs, it is valuable to establish a consensus on “common indices” for clinical trials, as suggested for non-orthodontic individuals [14]. The current SR identifies the orthodontic modification of the Silness and Löe plaque index (OMPI) [64], GI [63, 66, 67] and its modifications, and bleeding on probing as the most frequently used indices for PS, GS, and BS separately. This suggests that these indices could serve as “common indices” in future research for evaluating plaque and gingivitis in an orthodontic patient group.

Heterogeneity may also be the result of the subjective nature of the indices used to score PS and GS. Most plaque indices visually assess plaque presence or coverage on tooth surfaces [61,63–65, 62, 94]. Similarly, most gingivitis indices rely on visual signs such as color, consistency, contour, and spontaneous bleeding to quantify gingivitis severity [63, 66–68, 95]. This subjectivity makes consistent scoring challenging, resulting in low inter-examiner reliability [96]. Therefore, more standardized and objective indices are needed. Digital plaque imaging analysis, which evaluates plaque coverage through photographic analysis, offers a more objective and sensitive measure by enabling reproducible assessments from permanent fluorescence images [93, 97, 98]. However, because these intraoral images are captured using extraoral cameras positioned at a fixed position to participants, only the anterior teeth can currently be assessed [93, 97, 98]. Another relatively novel approach is 3D imaging using intraoral scanners [99]. As intraoral scanners can image the entire dental arch in a relatively short time and all areas can be easily assessed by rotating the 3D model, this approach has merged as a promising tool for comprehensively detecting and monitoring plaque levels [99]. Furthermore, an automatic identification method that combines intraoral scanning with deep learning algorithms has demonstrated high accuracy in detecting surface characteristics of gingival inflammation, enhancing the consistency and reliability of its evaluation [100]. However, the comprehensiveness of intraoral scanning comes at the expense of image quality, and further technological advancements are needed to address this limitation [99].

### Novelty and Hawthorne effects in short-term studies

As previously noted, both short- and long-term studies were included and analyzed together to provide a comprehensive evaluation of toothbrush efficacy; however, short-term trials may raise concerns about potential Hawthorne and novelty effects [101, 102]. The novelty effect refers to a positive effect relates to the newness of a device rather than its inherent performance [102]. Over time, this effect wears off, and the true effectiveness of the intervention may prove limited or even insignificant [102].

Although eight included studies had short intervention durations (≤4 weeks) [40, 45, 49, 51, 53, 55, 57, 60], only three borderline-duration trials (4 weeks) contributed to the meta-analyses [40, 53, 55]. Compared with the large number of comparisons in the overall meta-analyses (10–14 comparisons), any novelty effects present in these studies were likely limited and would have had minimal influence on the pooled results. Furthermore, the GRADE assessment accounted for heterogeneity. Thus, any potential novelty effects from these short-term studies are unlikely to have meaningfully impacted the reliability of the current findings.

### Implications of sensitivity analysis and publication bias

While restricting inclusion to published, English-language studies was justified, it remained essential to examine potential biases within the included trials. Accordingly, sensitivity analyses and publication-bias assessments were conducted to evaluate the influence of individual studies, methodological quality, industry involvement and study design on the pooled estimates (Supplementary Material S7 and S8).

These analyses indicated that certain studies appeared to drive the statistically significant findings favoring PTBs in the overall meta-analyses. Specifically, five studies influenced PS [39, 42, 44, 55, 58], three studies GS [39, 42, 55], and four studies BS results [39, 42, 55, 56]. Their considerable influence may be explained by several factors. Two studies received industry support [44, 55], which may be associated with publication bias through selective non-reporting of negative findings [19]. Correspondingly, sensitivity analyses excluding such studies showed no significant differences between PTBs and MTBs for PS and GS. Five studies had moderate to high risk of bias [42, 44, 55, 56, 58], potentially producing invalid results [19]; and sensitivity analyses including only low-risk-of-bias studies likewise showed no significant differences between PTBs and MTBs for PS and GS. Additionally, one study had a large sample size [39], resulting in greater weighting in the meta-analysis.

Despite these influences, these studies were retained in this SR, as the GRADE evaluation also incorporated risk of bias, heterogeneity, and publication bias, ensuring cautious and reliable conclusions.

### Limitations and recommendations for future research

The first limitation of the current SR is the small population size in most included studies. Only 9 of the 23 met the recommended minimum of 30 patients per group for the ADA Seal of Acceptance for toothbrushes [39, 41, 43, 44, 50–54]. Thus, larger sample sizes are suggested for future clinical trials.

Secondly, the included RCTs exhibited low to high risk of bias, resulting in a one-level downgrade in evidence quality. The main concerns involved inadequate reporting of the randomization process and selective result reporting, highlighting the need for more rigorously designed and transparently reported trials.

Thirdly, incomplete data limited both descriptive and meta-analyses, and the RIS was not reached in most TSAs, reducing the robustness of our findings. This limitation is further emphasized by the fact that CR-PTBs, although effective for plaque removal compared with MTBs, are no longer widely available. The current market is dominated by OR and sonic designs, creating a gap between evidence and the products accessible to consumers. These factors underscore the need for additional clinical trials with standardized outcome reporting, particularly for PTBs with OR and sonic action modes, which are widely used.

## Conclusion

For orthodontic patients with FAs, low-quality evidence limited by methodological weaknesses and inconsistency suggests that, compared with MTBs, PTBs may moderately improve PS, with medium to large effects on gingival parameters. Subanalysis by action-mode showed that only CR-PTBs provided large improvements in PS over MTBs, supported by very low-quality evidence. These findings observed in this SR are sensitive to a limited number of studies and should therefore be interpreted cautiously, as true effects remain uncertain and may change with more robust randomized evidence.

## Supplementary Material

cjag026_Supplementary_Data

## Data Availability

The data supporting this article can be found within the article and in its online Supplementary material.
