# Supplementary material for: Powered vs. manual toothbrushes in fixed orthodontic patients: a systematic review and meta-analysis
Source: Eur J Orthod. 2026 Jun 11;48(4):cjag026. doi: 10.1093/ejo/cjag026 (PMC13256007; doi:10.1093/ejo/cjag026)
Supplement: cjag026_Supplementary_Data [file cjag026_supplementary_data.docx]

**Powered vs. Manual Toothbrushes in Fixed Orthodontic Patients**

***-A systematic review and meta-analysis-***

**Supplementary Materials**

**S1.** Papers excluded with details for rejection.

**S2.** Overview of the studies processed for data extraction and further analyses.

**S3A.** Summary of plaque indices heterogeneity

**S3B.** Summary of gingivitis indices heterogeneity

**S3C.** Summary of gingival bleeding indices heterogeneity

**S4A.** Potential risk of bias judgement of the individual studies that were included for this review (**RoB 2** was used for RCTs).

**S4B.** Potential risk of bias judgement of the individual studies that were included for this review (**ROBINS-I** was used for CCTs).

**S5A.** Overview of extracted data of **Plaque scores** after **the longest follow-up** of the selected studies with various indices and their modifications. Baseline, end-trial measurements and incremental differences are presented as the (adjusted) means and standard deviations (SD) shown in parentheses. Statistically significant changes within groups are presented.

**S5B.** Overview of extracted data of **Gingivitis scores** after **the longest follow-up** of the selected studies with various indices and their modifications. Baseline, end-trial measurements and incremental differences are presented as the (adjusted) means and standard deviations (SD) shown in parentheses. Statistically significant changes within groups are presented.

**S5C.** Overview of extracted data of **Gingival** **Bleeding scores** after **the longest follow-up** of the selected studies with various indices and their modifications. Baseline, end-trial measurements and incremental differences are presented as the (adjusted) means and standard deviations (SD) shown in parentheses. Statistically significant changes within groups are presented.

**S6A.** Forrest Plots of the overall meta-analysis for **Plaque scores**, subgroup meta-analyses for the same plaque index, subgroup meta-analyses for plaque scores based on the same action mode of powered toothbrushes, and subgroup meta-analyses based on the same plaque index and action mode of powered toothbrushes after the longest follow up. SMD or MD are presented for the baseline, end-trial and incremental difference scores using a random-effects model.

**S6B.** Forrest Plots of the overall meta-analysis for **Gingivitis scores**, subgroup meta-analyses for the same gingival index, subgroup meta-analyses for gingivitis scores based on the same action mode of powered toothbrushes, and subgroup meta-analyses based on the same gingival index and action mode of powered toothbrushes after the longest follow up. SMD or MD are presented for the baseline, end-trial and incremental difference scores using a random-effects model.

**S6C.** Forrest Plots of the overall meta-analysis for **Gingival** **Bleeding scores**, subgroup meta-analyses for the same bleeding index, subgroup meta-analyses for gingival bleeding scores based on the same action mode of powered toothbrushes, and subgroup meta-analyses based on the same bleeding index and action mode of powered toothbrushes after the longest follow up. SMD or MD are presented for the baseline, end-trial and incremental difference scores using a random-effects model.

**S7. Sensitivity analyses** of overall meta-analyses with end-trial data.

**S8. Publication bias** assessment of end-trial meta-analyses.

**S9. Trial Sequential Analysis (TSA)** results**.**

**S10A.** Estimated evidence profile and strength of the recommendation on the efficacy of PTBs with different action modes vs. MTBs for reducing **Plaque scores** in orthodontic patients with fixed appliances.

**S10B.** Estimated evidence profile and strength of the recommendation on the efficacy of PTBs with different action modes vs. MTBs for reducing **Gingivitis scores** in orthodontic patients with fixed appliances.

**S10C.** Estimated evidence profile and strength of the recommendation on the efficacy of PTBs with different action modes vs. MTBs for reducing **Gingival** **Bleeding scores** in orthodontic patients with fixed appliances.

**S11.** A descriptive presentation of Supplementary S2-3 and other relevant information.

**S1.** Papers excluded with details for rejection.

| **Excluded** | |
| --- | --- |
| Full texts are not available | Borutta & Fischer (2000), Doll et al. (1999), Heasman et al. (1997), Cunha & Carvelho (1994), Fernandez et al (1994), Asmar et al. (1992), Fenwick & Williams (1985), Long & Killoy (1985), Zachrisson & Zachrisson (1971) |
| Using interdental devices (e.g., dental floss, interdental brushes, wood sticks, oral irrigators, etc.) | Johal et al. (2023), Nammi et al. (2019), Erbe et al. (2019), Marini et al. (2014), Heintze et al. (1996), Burch et al. (1994) |
| Without fixed appliances | Ferrari Peron et al. (2025), Statie et al. (2024), Agrawal et al. (2023), Al-Omiri et al. (2023), Al-Omiri et al. (2021), Nieri et al (2020), Erbe et al. (2018) |
| Undefined powered toothbrushes | Kim et al. (2024), Saluja et al. (2024), Shilpa et al. (2019), Saruttichart et al. (2017), Sharma et al. (2015), Ousehal et al. (2011), Borutta et al. (2002), Boyd et al. (1989) |
| Multi-product interventions. | Sharma et al. (2025), Zingler et al. (2014) |
| No powered toothbrushes tested | Matić et al. (2011) |
| One subject using both toothbrushes simultaneously | Jongsma et al. (2015), Womack et al. (1968) |
| No control group | Yankell et al. (1985) |
| Non-English papers | Lin et al (2014), Park et al. (2005), Park et al. (2005) |
| Single-brushing studies | Koretsi (2022), Erbe et al. (2013) |

**S2.** Overview of the studies processed for data extraction and further analyses.

| **Authors**  **Publication year**  **Study design**  **Intervention duration**  **Setting**  **Risk of bias** | **Participant number**  **(baseline/endpoint)**  **Sex (**♀/♂**)**  **Age (mean/range)**  **Oral prophylaxis (OP)** | **Comparison (PTB [manufacturers, location; action mode] / MTB [manufacturers, location])**  **Regimen (technique/frequency/duration)** | **Toothpaste (TP [manufacturers])**  **Other oral hygiene products or practices (OHP)**  **Compliance strategy** | **Outcomes**  **Study’s original conclusion** |
| --- | --- | --- | --- | --- |
| **Erden & Camcı**  **2023**  RCT, single-blinded, parallel  12 weeks  Turkey  Some concerns | 40 (40)  ♀: 20 (50%◊)  ♂: 20 (50%◊)  Mean age: 15.39  Age range: 12-18  OP: ? | **Comparison:**   - **PTB:** 3D Oral-B Genius 8900 with the EB20 brush head [Procter & Gamble, Marktheidenfeld, Germany; oscillating-rotating-pulsating] - **MTB:** Oral-B Orthodontic TB [Procter & Gamble, Dublin, Ireland]   **Regimen:**   - PTB: “daily clean” mode   MTB: modified Bass technique   - Per day: ? - Per time: ? | **TP:**  The same TP, Colgate Triple Action [Colgate-Palmolive, China]  **Other OHP:**  No  **Compliance:**  ? | PS, GS, BS  The PTB was more effective at removing plaque than the MTB. The results of the gingival index, on the other hand, did not reflect the plaque scores. |
| **Kumar et al.**  **2023**  RCT, single-blinded, parallel  2 months  India  Low risk of bias | 144◊ (140◊)  ♀: ? (?)  ♂: ? (?)  Mean age: ?  Age range: 13-35  OP: no within the past 1 month | **Comparison:**   - **PTB:** motorized TB [manufacturers ?, location ?; action mode ?] - **MTB:** physically driven TB [manufacturers ?, location ?]   **Regimen:**   - Technique: ? - Per day: ? - Per time: ? | **TP:**  ? [?]  **Other OHP:**  ?  **Compliance:**   - A compliance worksheet - Daily reminders through SMS | PS, GS, BS  At the one-month and two-month follow-ups, it was noted that the values of plaque, gingival and bleeding indices significantly decreased in the study participants using PTBs. |
| **Manphibool et al.**  **2023**  RCT, single-blinded, crossover (w.o. 4 week)  4 weeks  Thailand  Some concerns | 24 (23)  ♀: 18* (78%*)  ♂: 5* (22%*)  Mean age: 21.2*  Age range: 16-31  OP: ? | **Comparison:**   - **PTB:** WHITENGO™ LED TB [manufacturers ?, UK; sonic] - **MTB:** Systema OD orthodontic TB [Lion Company, Tokyo, Japan]   **Regimen:**   - Bass technique - Per day: 2 times - Per time: ? | **TP:**  The same TP, Sensodyne repair and protect [GSK™, Thailand]  **Other OHP:**  ?  **Compliance:**  ? | PS, GS  Clinically, the PTB was not more effective in reducing dental plaque or gingival inflammation  than the MTB in orthodontic patients with fixed appliances. |
| **Mylonopoulou et al.**  **2021**  RCT, single-blinded, parallel  3 months  Greece  Low risk of bias | 80 (80)  ♀: 40◊ (50%◊)  ♂: 40◊ (50%◊)  Mean age: 14.1◊  Age range: ≤16  OP: ? | **Comparison:**   - **PTB:** 3D Oral-B Pro-1000 with the Oral-B Ortho brush head [Procter & Gamble, Cincinnati, Ohio; oscillating-rotating-pulsating] - **MTB:** Oral-B orthodontic TB [Procter & Gamble, location ?]   **Regimen:**   - Technique: ? - Per day: 2 times - Per time: 2 mins | **TP:**  ? [?]  **Other OHP:**   - no other TBs, TP, whitening products, or tooth cleaning - no oral hygiene comments or instructions from treating clinicians   **Compliance:**  ? | PS, GS, BS  No difference in plaque removal efficacy and gingival inflammation reduction was found between the PTBs and MTBs in adolescents with fixed orthodontic appliances. |
| **Bilen et al.**  **2021**  RCT, single-blinded, parallel  4 months  Turkey  Some concerns | 36 (36◊)  ♀: 18 (50%◊)  ♂: 18 (50%◊)  Mean age: 14.5◊  Age range: 12-18  OP: ? | **Comparison:**   - **PTB:** Oral-B Interactive 8900 with the Oral-B 8D16u/EB50 Cross Action brush head [Procter & Gamble, location ?; counter-rotational action] - **MTB:** Oral-B Orthodontic TB [Procter & Gamble, Albany, GA]   **Regimen:**   - PTB: manufacturer's instructions;   MTB: modified Bass technique   - Per day: 2 times - Per time: 2 mins | **TP:**  The same TP, Colgate [Colgate-Palmolive Company, Warsaw, Poland]  **Other OHP:**  No IDB, MW, or any other mechanical or chemical cleaning products  **Compliance:**  ? | PS, GS, BS  PTBs were found more effective than MTBs in patients undergoing fixed orthodontic treatment due to the significant changes in periodontal parameters after the second month of the study. |
| **Naqvi et al.**  **2020**  CCT, parallel  8 weeks  Saudi Arabia  Serious risk of bias | 60 (?)  ♀: ? (?)  ♂: ? (?)  Mean age: ?  Age range: ?  OP: ? | **Comparison:**   - **PTB 1:** ? [manufacturers ?, location ?; action mode ?] - **PTB 2:** Sonic TB [manufacturers ?, location ?; sonic] - **MTB:** Orthodontic TB [manufacturers ?, location ?]   **Regimen:**   - Technique: ? - Per day: 2 times - Per time: 2 mins | **TP:**  ? [?]  **Other OHP:**  ?  **Compliance:**  ? | PS, GS, BS  Sonic PTBs were effective in reducing plaque, gingival and interdental bleeding indices followed by PTBs and MTBs. |
| **Erbe et al.**  **2019**  RCT, single-blinded, parallel  6 weeks  Germany  Some concerns | 60 (58◊)  ♀: 30 (50%)  ♂: 30 (50%)  Mean age: 14.5  Age range: 13-17  OP: no OP within the past 4 weeks | **Comparison:**   - **PTB:** Interactive Oral-B Professional Care 6000 D36 with the Oral-B Precision Clean brush head EB20 [Procter & Gamble, Cincinnati, Ohio; oscillating-rotating-pulsating] - **MTB:** Oral-B Indicator 35 soft TB [Procter & Gamble, location ?]   **Regimen:**   - PTB: manufacturer's instructions;   MTB: customary manner   - Per day: 2 times - Per time: at least 2 mins | **TP:**  The same TP, Blend-a-Med Classic (1450 ppm NaF) [Procter & Gamble, Gross Gerau, Germany]  **Other OHP:**  No  **Compliance:**  ? | PS  A PTB generated significantly greater plaque removal versus an MTB. |
| **Farhadian et al.**  **2015**  RCT, single-blinded, parallel  2 weeks  Iran  High risk of bias | 36◊ (?)  ♀: ? (?)  ♂: ? (?)  Mean age: 18.6  Age range: 13.8-23.4◊  OP: ? | **Comparison:**   - **PTB:** Oral-B Vitality TB [Procter & Gamble, Kronberg, Germany; oscillating-rotating] - **MTB:** Oral-B orthodontic TB [Procter & Gamble, Kildare, Ireland]   **Regimen:**   - Modified Bass technique - Per day: at least 2 times - Per time: 2-4 mins | **TP:**  ? [?]  **Other OHP:**  ?  **Compliance:**  ? | PS, GS, BS  In the entire oral cavity, there was a statistically significant improvement in plaque, gingival and bleeding indices in all the groups except for hyperplastic index. No significant differences were found between the PTB and MTB groups. |
| **Silvestrini Biavati et al.**  **2010**  RCT, parallel  8 weeks  Italy  High risk of bias | 20 (20◊)  ♀: 12 (60%◊)  ♂: 8 (40%◊)  Mean age: 11.4  Age range: 10-14  OP: no | **Comparison:**   - **PTB:** Oral-B Professional Care 8500 with the Oral-B Ortho Refill orthodontic brush head [Procter & Gamble, location ?; oscillating-rotating-pulsating] - **MTB:** Oral-B 35 orthodontic TB [Procter & Gamble, location ?]   **Regimen:**   - PTB: manufacturer's instructions;   MTB: rotation/vibration technique   - Per day: 2 times - Per time: at least 2 mins | **TP:**  The same TP, Oral-B Teeth and Gums, 0.4% stabilized stannous fluoride [?]  **Other OHP:**  No  **Compliance:**  ? | PS, BS  With respect to the orthodontic MTB, the PTB was found to better improve both plaque and bleeding indices. |
| **Sadiq & Badea**  **2009**  RCT, parallel  8 weeks  Iraq  High risk of bias | 54 (?)  ♀: 25◊ (46%◊)  ♂: 29◊ (54%◊)  Mean age: 14.9◊  Age range: 10-20  OP: no OP within the past 4 weeks | **Comparison:**   - **PTB:** ? [Triza, Switzerland; action mode ?] - **MTB:** ? [Triza, Switzerland]   **Regimen:**   - Technique: ? - Per day: 2 times - Per time: ? | **TP:**  The same fluoride TP free from antiplaque and anti-calculus agents [?]  **Other OHP:**   - The same fluoride MW, rinsing with 10 ml once daily - No interdental cleaning aids   **Compliance:**  ? | PS, GS, BS  The reduction of the values of all parameters in PTB group is higher than in MTB group and the difference is statistically significant. Thus, the PTB is real alternative to the often laborious MTB used during active appliance therapy. |
| **Costa et al.**  **2007**  RCT, single-blinded, crossover (w.o. 2 weeks)  30 days  Brazil  High risk of bias | 21 (21)  ♀: 10 (48%◊)  ♂: 11 (52%◊)  Mean age: 15.2  Age range: 12-18  OP: ? | **Comparison:**   - **PTB**: Braun Oral-B 3D Plaque Remover [Braun GmbH, Kronberg, Germany; oscillating-rotating-pulsating] - **MTB**: Oral-B Model 30 [Procter & Gamble, Gillete do Brasil, Manaus, Brazil]   **Regimen:**   - PTB 1 & 2: manufacturer's instructions;   MTB: Bass technique   - Per day: 3 times - Per time: 2 mins | **TP:**  The same TP, Sorriso [Colgate-Palmolive Indústriae Comércio Ltda., São Bernardo do Campo, SP, Brazil]  **Other OHP:**  No  **Compliance:**  ? | PS, BS  When the two brush groups were compared for each time point, there were no significant differences in any of the study parameters. |
| **Kossack & Jost-Brinkmann**  **2005**  RCT, single-blinded, crossover (w.o. 2 weeks)  4 weeks  Germany  High risk of bias | 20◊ (?)  ♀: ? (?)  ♂: ? (?)  Mean age: 13.9  Age range: 12-22  OP: ? | **Comparison:**   - **PTB**: Water Pik Sonic Speed SR-100E [Intersanté, Bensheim, Germany; sonic] - **MTB**: Elmex interX short brush-head [GABA, Lörrach, Germany]   **Regimen:**   - Technique: ? - Per day: ? - Per time: ? | **TP:**  The same Elmex TP [?]  **Other OHP:**  ?  **Compliance:**  ? | PS, BS  The sonic PTB does not clean teeth more effectively than does the new generation elmex interX short brush-head MTB. |
| **Hickman et al.**  **2002**  RCT, single-blinded, parallel  8 weeks  ?  High risk of bias | 63 (60)  ♀: 35◊ (56%◊)  ♂: 28◊ (44%◊)  Mean age: 15.1◊  Age range: 10-20  OP: no OP within the past 4 weeks | **Comparison:**   - **PTB:** Braun Oral-B Plaque Remover D5 with the orthodontic brush head [Braun AG, Germany; oscillating-rotating] - **MTB:** Reach Compact Head Medium TB [Johnson and Johnson, Maidenhead, UK]   **Regimen:**   - Technique: ? - Per day: 2 times - Per time: 2 mins | **TP:**  The same fluoride-containing TP, Colgate Great Regular Flavor [Colgate-Palmolive Ltd, Guildford, UK]  **Other OHP:**   - The same fluoride MW, Colgate Fluorigard [Colgate-Palmolive Ltd, Guildford, UK], rinsing with 10 ml once daily - No IDB   **Compliance:**  Written instructions and a toothbrushing diary | PS, GS, BS  There were no measurable differences between the PTB with modified orthodontic brush head and an MTB with respect to mean change in plaque, gingivitis, or interdental bleeding scores when used by patients wearing fixed appliances. |
| **Thienpont et al.**  **2001**  RCT, single-blinded, crossover (w.o. 4 weeks)  4 weeks  Belgium  High risk of bias | 33 (33)  ♀: 18 (54.5%◊)  ♂: 15◊ (45.5%◊)  Mean age: ?  Age range: 11.1-20.4◊  OP: yes, after scoring | **Comparison:**   - **PTB 1:** Braun Oral-B 3D Plaque Remover with the orthodontic brush head [Braun GmbH, Kronberg, Germany; oscillating-rotating-pulsating] - **PTB 2:** Philips-Jordan HP 510 with the standard brush head [Philips Domestic Appliances, Groningen, The Netherlands; oscillating-rotating] - **MTB 1:** Oral-B Advantage Control Grip TB [Braun, location ?] - **MTB 2:** Lactona orthodontic TB [Bergen op Zoom, The Netherlands]   **Regimen:**   - PTB 1 & 2: manufacturer's instructions;   MTB 1 & 2: modified Bass technique   - Per day: 2 times - Per time: 3 mins | **TP:**  The same TP, Oral-B (Braun)  **Other OHP:**  No topical fluoride application, dental floss, IDB, MW, or other oral hygiene aids  **Compliance:**  ? | PS, GS, BS  This study found no difference in efficacy among the 2 MTBs and the 2 PTBs in children undergoing fixed orthodontic appliance therapy concerning gingival index, bleeding on probing index, and plaque index. |
| **Clerehugh et al.**  **1998**  RCT, single-blinded, parallel  8 weeks  UK  Low risk of bias | 84 (79◊)  ♀: 47 (56%◊)  ♂: 37 (44%◊)  Mean age: ?  Age range: 10-20  OP: no OP within the past 4 weeks | **Comparison:**   - **PTB:** Braun Oral-B Plaque Remover D5 with the orthodontic brush head OD5 [Oral-B UK, Isleworth, UK; oscillating-rotating] - **MTB:** Reach Compact Head Medium conventional TB [Johnson and Johnson, Maidenhead, UK]   **Regimen:**   - Technique: ? - Per day: 2 times - Per time: 2 mins | **TP:**  The same fluoride-containing TP, Colgate Great Regular Flavor [Colgate-Palmolive Ltd, Guildford, UK]  **Other OHP:**   - The same 0.05% sodium fluoride MW, Colgate Fluorigard [Colgate-Palmolive Ltd., Guildford, UK], rinsing with 10 ml daily - No other oral hygiene aids   **Compliance:**  A toothbrushing diary | PS, GS, BS  Use of a PTB with an orthodontic brush head may be of benefit in promoting gingival health in fixed orthodontic appliance patients. |
| **Heasman et al.**  **1998**  RCT, single-blinded, crossover (no w.o.)  4 weeks  UK  High risk of bias | 60 (60)  ♀: 39 (65%◊)  ♂: 21 (35%◊)  Mean age: 13.6  Age range: 10-16  OP: yes, after week 4 and week 8 scoring | **Comparison:**   - **PTB 1:** Braun Oral-B Plaque Remover D7 with the orthodontic brush head OD5-1 [Braun AG, Germany; oscillating-rotating] - **PTB 2:** Dental Logic HP550 with the regular brush head HP5924 [Philips, U.K.; sonic] - **MTB:** Oral-B P35 orthodontic TB [Oral-B Laboratories, Calif.]   **Regimen:**   - PTB 1 & 2: manufacturer's instructions;   MTB: combination of Bass and Charters techniques   - Per day: 2 times - Per time: at least 3 mins for PTBs 1 & 2; at least 2 mins for MTBs | **TP:**  The same fluoride-containing TP [?]  **Other OHP:**  ?  **Compliance:**  ? | PS, BS  The two types of PTBs, and the orthodontic MTB were equally effective in removing plaque and reducing gingival inflammation as indicated by bleeding on probing in patients undergoing fixed orthodontic treatment. |
| **Trimpeneers et al.**  **1997**  RCT, single-blinded, crossover (w.o. 1 month)  2 months  Belgium  High risk of bias | 36 (35◊)  ♀: 19 (53%◊)  ♂: 17 (47%◊)  Mean age: 12.8◊  Age range: 11.4-15.2◊  OP: yes, after baseline and every month 2 scoring | **Comparison:**   - **PTB 1:** Interplak TB [Bausch & Lomb, location ?; counter-rotational action] - **PTB 2:** Philips [Philips, location ?; action mode ?] - **PTB 3:** Rotadent with hollow cup-shaped TB [Novitas, location ?; rotary action] - **MTB:** Blend-a-Med Dental Plus Junior TB with medium stiffness [Proctor & Gamble, location ?]   **Regimen:**   - PTB 1 & 2 & 3: manufacturer's instructions;   MTB: modified Bass technique   - Per day: 2 times - Per time: 3 mins | **TP:**  The same fluoride-containing TP, Blend-a-Med (1450 ppm NaF) [Procter & Gamble]  **Other OHP:**   - No dental floss, IDB or other oral hygiene aids - No topical fluoride applications, no rinse with fluoride solutions nor any oral antiseptics   **Compliance:**  ? | PS, GS, BS  For all parameters, the MTB was the most effective. Of the three PTBs tested, the Philips PTB seemed to give slightly better results than the Interplak toothbrush, whereas Rotadent very clearly gave results inferior to all others. |
| **Ho & Niederman**  **1997**  RCT, single-blinded, parallel  4 weeks  US  High risk of bias | 24 (?)  ♀: 12 (50%◊)  ♂: 12 (50%◊)  Mean age: 15  Age range: 11-17  OP: ? | **Comparison:**   - **PTB:** Sonicare TB [Optiva Corp., Bellevue, WA; sonic] - **MTB:** Oral-B P35 TB [Oral-B Laboratories, Belmont, CA]   **Regimen:**   - PTB: manufacturer's instructions;   MTB: Bass technique   - Per day: 2 times - Per time: 2 mins | **TP:**  The same TP, Crest Sparkle [Procter & Gamble, Cincinnati, OH].  **Other OHP:**  ?  **Compliance:**  Parental reinforcement at home | PS, GS, BS  The PTB is superior to an MTB in improving periodontal health in adolescent orthodontic patients with existing gingivitis. |
| **White**  **1996**  CCT, parallel  Around 3 months  ?  Moderate risk of bias | 40 (32◊)  ♀: ?  ♂: ?  Mean age: ?  Age range: ?  OP: ? | **Comparison:**   - **PTB:** Sonicare TB [manufacturers ?, location ?; sonic] - **MTB:** multi-tufted TB [manufacturers ?, location ?]   **Regimen:**   - Modified Bass technique - Per day: 2 times - Per time: 2 mins | **TP:**  The same TP, Colgate [?]  **Other OHP:**  No other oral hygiene aids such as floss, MW, or oral irrigators.  **Compliance:**  ? | PS, BS  The Sonicare PTB may help many orthodontic patients improve their oral health and, consequently, lower their susceptibility to dental disease. |
| **Trombeli et al.**  **1995**  RCT, single-blinded, crossover (no w.o.)  2 weeks  Italy  High risk of bias | 20 (20)  ♀: 9 (45%◊)  ♂: 11 (55%◊)  Mean age: 15.5  Age range: 11-26  OP: yes, after baseline and week 2 scoring | **Comparison:**  **PTB:** Interplak TB [Dental Research Corporation, location ?; counter-rotational action]  **MTB:** GUM 311 conventional single-headed TB [Butler, location ?]  **Regimen:**   - PTB: manufacturer's instructions;   MTB: modified Bass technique   - Per day: at least 2 times - Per time: 2 mins | **TP:**  ? [?]  **Other OHP:**  No other oral hygiene devices, such as floss, rubber tip, or IDB, or CHX-TP and MW  **Compliance:**  ? | PS  The result of this study indicated the superiority of counter-rotational PTBs over MTBs in removing supragingival plaque in patients with fixed appliances during a 2-week period. |
| **Wilcoxon et al.**  **1991**  CCT, single-blinded crossover (no w.o.)  1 month  US  Moderate risk of bias | 20 (?)  ♀: 12 (60%◊)  ♂: 8 (40%◊)  Mean age: ?  Age range: 12-53  OP: yes, after post brushing scoring | **Comparison:**   - **PTB:** Interplak TB [Dental Research Corporation, Tucker, Ga.; counter-rotational action] - **MTB:** Oral B-15 soft-bristle orthodontic TB [Cooper Care, Palo Alto, Calif.]   **Regimen:**   - PTB: manufacturer's instructions;   MTB: modified Bass technique   - Per day: at least 2 times - Per time: ? | **TP:**  ? [?]  **Other OHP:**  No other oral hygiene aids, such as floss  **Compliance:**  ? | PS, GS  Patients with fixed orthodontic appliances who use a counter-rotational PTB more effectively remove their dental plaque and can have better gingival health with the same amount of effort than those patients who use MTBs. |
| **Jackson**  **1991**  CCT, single-blinded, crossover (no w.o.)  at least 4 weeks  US  Low risk of bias | 20 (20)  ♀: 12 (60%◊)  ♂: 8 (40%◊)  Mean age: ?  Age range: ?  OP: yes, after baseline scoring | **Comparison:**   - **PTB:** TB with 2500 arc-shaped vertical movements per minute and brushing angle from 0° to 60° [manufacturers ?, location ?; action mode ?] - **MTB:** patients’ own TB [manufacturers ?, location ?]   **Regimen:**   - PTB: manufacturer's instructions;   MTB: instructions for orthodontic patients   - Per day: 2 times - Per time: 1 min | **TP:**  ? [?]  **Other OHP:**  No other oral hygiene aids, such as floss or a Proxabrush  **Compliance:**  ? | PS, GS  There were no significant differences between the means for plaque and gingival health in PTB and MTB groups. |
| **van Venrooy et al.**  **1985**  RCT, single-blinded, crossover (no w.o.)  1 week  US  High risk of bias | 24 (24)  ♀: ? (?)  ♂: ? (?)  Mean age: ?  Age range: 12-30  OP: yes, after baseline and week 1 scoring | **Comparison:**   - **PTB:** Interplak TB [manufacturers ?, location ?; counter-rotational action] - **MTB:** Oral-B 40 TB [manufacturers ?, location ?]   **Regimen:**   - PTB: manufacturer's instructions;   MTB: modified Bass technique   - Per day: as many times as possible for PTB; unknown for MTB - Per time: ? | **TP:**  No TP use for PTB group, unknown for MTB group  **Other OHP:**  ?  **Compliance:**  ? | PS  The Interplak PTB facilitates plaque removal for patients in fixed orthodontic appliances. |

♀: female, ♂: male, ◊: calculated by the authors of this review based on the data of the selected paper, ?: not reported/unknown, *: end-trial data, RCT: randomized controlled clinical trial, CCT: controlled clinical trial, OP: oral prophylaxis, w.o.: washout period for a crossover design, TB: toothbrush, MTB: manual toothbrush, PTB: powered toothbrush, TP: toothpaste, OHP: oral hygiene products or practices, LED: light emitting diode, NaF: sodium fluoride, ADA: American Dental Association, IDB: interdental brushes, MW: mouthwash, CHX: chlorhexidine, NA: not applicable

**S3A.** Summary of plaque indices heterogeneity

| **Heterogeneity**  **Study** | **Plaque indices** | | | | | | | | | **Region scored** | | | |
| --- | --- | --- | --- | --- | --- | --- | --- | --- | --- | --- | --- | --- | --- |
|  | **PI** | **QHPI** | **TMQH** | **OMPI** | **HAI** | **O’Leary PI** | **VPI** | **BPI** | **JOPI** | **Banded & bonded surfaces** | **Non-banded & bonded surfaces** | **Anterior region** | **Posterior region** |
| **Erden & Camcı**  **2023** | - | + | + | - | - | - | - | - | - | + | + | + | + |
| **Kumar et al.**  **2023** | + | - | - | - | - | - | - | - | - | ? | ? | ? | ? |
| **Manphibool et al.**  **2023** | + | - | - | - | - | - | - | - | - | + | + | + | + |
| **Mylonopoulou et al.**  **2021** | \| - \| - \| - \| \| --- \| --- \| --- \| | - | - | + | - | + | - | - | - | + | - | + | + |
| **Bilen et al.**  **2021** | - | - | - | + | - | - | - | - | - | ? | ? | ? | ? |
| **Naqvi et al.**  **2020** | + | - | - | - | - | - | - | - | - | ? | ? | ? | ? |
| **Erbe et al.**  **2019** | - | + | + | - | - | - | - | - | - | + | + | + | + |
| **Farhadian et al.**  **2015** | - | - | - | - | - | + | - | - | - | + | - | ? | ? |
| **Silvestrini Biavati et al.**  **2010** | - | - | - | - | - | + | - | - | - | ? | ? | ? | ? |
| **Sadiq & Badea**  **2009** | + | - | - | - | - | - | - | - | - | + | - | ? | ? |
| **Costa et al.**  **2007** | - | - | - | + | - | - | - | - | - | + | - | + | + |
| **Kossack & Jost-Brinkmann**  **2005** | - | + | - | - | - | - | - | - | - | + | + | + | + |
| **Hickman et al.**  **2002** | - | - | - | + | - | - | - | - | - | + | - | ? | ? |
| **Thienpont et al.**  **2001** | - | + | - | + | - | - | - | - | - | + | + | + | + |
| **Clerehugh et al.**  **1998** | - | - | - | + | - | - | - | - | - | + | - | ? | ? |
| **Heasman et al.**  **1998** | - | - | - | - | - | - | + | - | - | + | - | + | + |
| **Trimpeneers et al.**  **1997** | - | + | - | - | - | - | - | + | - | + | + | + | + |
| **Ho & Niederman**  **1997** | + | - | - | - | - | - | - | - | - | + | + | + | + |
| **White**  **1996** | - | - | - | - | + | - | - | - |  | + | - | + | - |
| **Trombeli et al.**  **1995** | - | - | - | - |  | + | - | - | - | + | - | + | + |
| **Wilcoxon et al.**  **1991** | - | - | - | - | - | + | - | - | - | + | + | + | + |
| **Jackson**  **1991** | - | - | - | - | - | - | - | - | + | + | - | ? | ? |
| **van Venrooy et al.**  **1985** | + | - | - | - | - | - | - | - | - | + | + | ? | ? |

+: used or assessed; -: unused or unassessed; ?: unknown; PI: plaque index by Silness & Löe (1964) ; QHPI: Quigley-Hein plaque index (1962) and its other modifications; TMQH: the Turesky-modified Quigley–Hein index (1970); OMPI: the Orthodontic modification of the Silness & Löe plaque index (1964) by Williams et al. (1991); HAI: hygiene analysis index; O’Leary PI: O’Leary plaque index (1972) and its modification; VPI: visible plaque index by Ainamo & Bay (1975); BPI: plaque index brackets; JOPI: Jackson orthodontic plaque index (1991)

: used or assessed

: unused or unassessed

: unknown

**S3B.** Summary of gingivitis indices heterogeneity

| **Heterogeneity**  **Study** | **Gingival indices** | | | | | **Region scored** | | | |
| --- | --- | --- | --- | --- | --- | --- | --- | --- | --- |
|  | **GI** | **LMGI** | **AGI** | **RGI** | **Banded & bonded surfaces** | | **Non-banded & bonded surfaces** | **Anterior region** | **Posterior region** |
| **Erden & Camcı**  **2023** | + | - | - | - | + | | ? | + | + |
| **Kumar et al.**  **2023** | + | - | - | - | ? | | ? | ? | ? |
| **Manphibool et al.**  **2023** | + | - | - | - | + | | + | + | + |
| **Mylonopoulou et al.**  **2021** | + | - | - | - | + | | - | + | + |
| **Bilen et al.**  **2021** | + | - | - | - | ? | | ? | ? | ? |
| **Naqvi et al.**  **2020** | + | - | - | - | ? | | ? | ? | ? |
| **Farhadian et al.**  **2015** | + | - | - | - | + | | - | ? | ? |
| **Sadiq & Badea**  **2009** | - | - | + | - | ? | | ? | ? | ? |
| **Hickman et al.**  **2002** | + | - | - | - | ? | | ? | ? | ? |
| **Thienpont et al.**  **2001** | - | + | - | - | + | | + | + | + |
| **Clerehugh et al.**  **1998** | + | - | - | - | + | | - | ? | ? |
| **Trimpeneers et al.**  **1997** | - | + | - | - | + | | + | + | + |
| **Ho & Niederman**  **1997** | + | - | - | - | + | | + | + | + |
| **Wilcoxon et al.**  **1991** | - | - | - | + | + | | + | + | + |
| **Jackson**  **1991** | + | - | - | - | + | | - | ? | ? |

+: used or assessed; -: unused or unassessed; ?: unknown; GI: Löe & Silness gingival index (1963,1964,1967) and its modification; LMGI: Lobene modified gingival index (1986) ; AGI: gingival index by Ainamo et al. (1982); RGI: Ramfjord gingival index (1959)

: used or assessed

: unused or unassessed

: unknown

**S3C.** Summary of gingival bleeding indices heterogeneity

| **Heterogeneity**  **Study** | **Bleeding indices** | | | | | | | **Region scored** | | | |  |
| --- | --- | --- | --- | --- | --- | --- | --- | --- | --- | --- | --- | --- |
|  | **BOP** | **MPBI** | **MGI-S** | **EIBI** | **BI** | **PBI** | **LSBI** | **Banded & bonded surfaces** | **Non-banded & bonded surfaces** | **Anterior region** | **Posterior region** | |
| **Erden & Camcı**  **2023** | + | - | - | - | - | - | - | + | ? | + | + | |
| **Kumar et al.**  **2023** | - | + | - | - | - | - | - | ? | ? | ? | ? | |
| **Mylonopoulou et al.**  **2021** | - | - | + | - | - | - | - | + | - | + | + | |
| **Bilen et al.**  **2021** | + | - | - | - | - | - | - | ? | ? | ? | ? | |
| **Naqvi et al.**  **2020** | - | - | - | + | - | - | - | ? | ? | ? | ? | |
| **Farhadian et al.**  **2015** | + | - | - | - | - | - | - | + | - | ? | ? | |
| **Silvestrini Biavati et al.**  **2010** | - | - | - | - | + | - | - | ? | ? | ? | ? | |
| **Sadiq & Badea**  **2009** | - | - | - | + | - | - | - | - | + | ? | ? | |
| **Costa et al.**  **2007** | - | - | - | - | - | - | + | + | - | + | + | |
| **Kossack & Jost-Brinkmann**  **2005** | - | - | - | - | - | + | - | ? | ? | + | + | |
| **Hickman et al.**  **2002** | - | - | - | + | - | - | - | - | + | ? | ? | |
| **Thienpont et al.**  **2001** | + | - | - | - | - | - | - | + | + | + | + | |
| **Clerehugh et al.**  **1998** | - | - | - | + | - | - | - | - | + | ? | ? | |
| **Heasman et al.**  **1998** | - | - | - | - | + | - | - | + | + | + | + | |
| **Trimpeneers et al.**  **1997** | + | - | - | - | - | - | - | + | + | + | + | |
| **Ho & Niederman**  **1997** | + | - | - | - | - | - | - | + | + | + | + | |
| **White**  **1996** | - | + | - | - | - | - | - | - | + | + | - | |

+: used or assessed; -: unused or unassessed; ?: unknown; BOP: bleeding on probing; MPBI: modified papillary bleeding index; MGI-S: the modified simplified gingival index by Lindhe (1984); EIBI: Eastman interdental bleeding index by Caton & Polson (1985); BI: gingival bleeding index by Ainamo and Bay (1975); PBI: papillary bleeding index by Mühlemann (1977); LSBI: Löe & Silness gingival index for bleeding

: used or assessed

: unused or unassessed

: unknown

**S4A.** Potential risk of bias judgement of the individual studies that were included for this review (**RoB 2** was used for RCTs).

| **Cochrane risk of bias**  **(RoB 2 for RCTs)** | **Randomization process** | **Period and carryover effects** | **Deviations from intended interventions** | **Missing outcome data** | **Measurement of the outcome** | **Selection of the reported result** | **Overall judgement** |
| --- | --- | --- | --- | --- | --- | --- | --- |
| **Erden & Camcı 2023** | ? |  | + | + | + | + | ? |
| **Kumar et al. 2023** | + |  | + | + | + | + | + |
| **Manphibool et al. 2023** | + | ? | + | + | + | + | ? |
| **Mylonopoulou et al. 2021** | + |  | + | + | + | + | + |
| **Bilen et al. 2021** | ? |  | + | + | + | + | ? |
| **Erbe et al. 2019** | ? |  | + | + | + | + | ? |
| **Farhadian et al. 2015** | ? |  | ? | ? | + | - | - |
| **Silvestrini Biavati et al. 2010** | + |  | + | + | - | - | - |
| **Sadiq & Badea 2009** | ? |  | ? | ? | - | - | - |
| **Costa et al. 2007** | ? | ? | + | + | + | - | - |
| **Kossack & Jost-Brinkmann 2005** | ? | ? | ? | ? | + | - | - |
| **Hickman et al. 2002** | + |  | + | + | + | + | - |
| **Thienpont et al. 2001** | ? | + | + | + | + | - | - |
| **Clerehugh et al. 1998** | + |  | + | + | + | + | + |
| **Heasman et al. 1998** | ? | + | + | + | + | - | - |
| **Trimpeneers et al. 1997** | ? | + | + | + | + | - | - |
| **Ho & Niederman 1997** | ? |  | ? | ? | + | + | - |
| **Trombeli et al. 1995** | ? | + | + | + | + | - | - |
| **van Venrooy et al. 1985** | ? | + | + | + | + | - | - |

+ : low risk of bias

? : some concerns

- : high risk of bias

RCT: Randomized controlled clinical trial

**S4B.** Potential risk of bias judgement of the individual studies that were included for this review (**ROBINS-I** was used for CCTs).

| **Cochrane risk of bias**  **(ROBINS-I for CCTs)** | **Confounding** | **Selection of participants into the study** | **Classification of interventions** | **Deviations from intended interventions** | **Missing data** | **Measurement of outcomes** | **Selection of the reported result** | **Overall judgement** |
| --- | --- | --- | --- | --- | --- | --- | --- | --- |
| **Naqvi et al. 2020** | x | + | + | + | ? | + | X | X |
| **White 1996** | x | + | + | + | - | + | ? | - |
| **Wilcoxon et al. 1991** | + | + | + | + | ? | + | + | - |
| **Jackson 1991** | + | + | + | + | + | + | + | + |

+ : low risk of bias

- : moderate risk of bias

X : serious risk of bias

! : critical risk of bias

? : no information

CCT: Controlled clinical trial

**S5A.** Overview of extracted data of **Plaque scores** after **the longest follow-up** of the selected studies with various indices and their modifications. Baseline, end-trial measurements and incremental differences are presented as the (adjusted) means and standard deviations (SD) shown in parentheses. Statistically significant changes within groups are presented.

| **Authors (year)**  **Intervention duration** | **Indices used for evaluation** | **Evaluation**  **Surfaces/ time** | **Groups** | **Included subjects**  **Baseline (endpoint)** | **Mean (SD)** | | | **Significant**  **within groups** |
| --- | --- | --- | --- | --- | --- | --- | --- | --- |
|  |  |  |  |  | **Baseline** | **Endpoint** | **Difference** |  |
| **Erden & Camcı**  **2023**  12 weeks | The modified Quigley–Hein index (QHPI)+ the Turesky-modified  Quigley–Hein index (TMQH,1970) | Buccal & lingual surfaces | **PTB:** 3D Oral-B Genius 8900 + Oral-B OBH | 20◊ (20◊) | 3.49 (0.50) | 2.39 (0.33) | -1.10◊ (0.44◊) | yes |
|  |  |  | **MTB:** Oral-B Orthodontic brush | 20◊ (20◊) | 3.56 (0.42) | 2.68 (0.37) | -0.88◊ (0.40◊) | yes |
| **Kumar et al.**  **2023**  2 months | Silness & Löe plaque index (PI, 1964) | ? | **PTB:** ? | 73 (70) | 1.4 (0.57) | 0.9 (0.47) | -0.5◊ (0.52◊) | yes |
|  |  |  | **MTB:** ? | 71 (70) | 1.3 (0.43) | 1.6 (0.46) | 0.3◊ (0.45◊) | yes |
| **Manphibool et al.**  **2023**  4 weeks | Silness & Löe plaque index (PI, 1964) | Bracket sides (proximal, gingival, incisal) | **PTB:** LED toothbrush | 24 (23) | 1.49 (0.49◊) | 1.36 (0.43◊) | -0.13 (0.34◊) | no◊ |
|  |  |  | **MTB:** Systema OD orthodontic toothbrush | 24 (23) | 1.58 (0.39◊) | 1.23 (0.43◊) | -0.36 (0.48◊) | yes◊ |
| **Mylonopoulou et al.**  **2021**  3 months | The Orthodontic modification of the Silness & Löe plaque index (1964) by Williams et al. (OMPI, 1991) | Labial surfaces (mesial, buccal, distal) | **PTB:** 3D Oral-B Pro-1000 + Oral-B OBH | 40 (40) | 0.31 (0.10) | 0.29 (0.10) | -0.02◊ (0.10◊) | no◊ |
|  |  |  | **MTB:** Oral-B Orthodontic brush | 40 (40) | 0.32 (0.11) | 0.29 (0.08) | -0.02◊ (0.10◊) | no◊ |
|  | Modified O’Leary plaque index (O’Leary PI) | Labial surfaces (mesial, buccal, distal) | **PTB:** 3D Oral-B Pro-1000 + Oral-B OBH | 40 (40) | 0.92 (0.14) | 0.92 (0.12) | 0.00◊ (0.11◊) | no◊ |
|  |  |  | **MTB:** Oral-B Orthodontic brush | 40 (40) | 0.91 (0.14) | 0.92 (0.10) | 0.01◊ (0.12◊) | no◊ |
| **Bilen et al.**  **2021**  4 months | The Orthodontic modification of the Silness & Löe plaque index (1964) by Williams et al. (OMPI, 1991) | ? | **PTB:** Oral-B Interactive 8900 + Oral-B CBH | 18 (18) | 1.05 (0.13) | 0.68 (0.19) | -0.37◊ (0.19) | yes |
|  |  |  | **MTB:** Oral-B Orthodontic brush | 18 (18) | 1.00 (0.23) | 0.78 (0.28) | -0.22◊ (0.17) | yes |
| **Naqvi et al.**  **2020**  8 weeks | Silness & Löe plaque index (PI, 1964) | ? | **PTB 1:** ? | 20 (?) | 1.14 (?) | 1.04 (?) | -0.10◊ (?) | yes |
|  |  |  | **PTB 2:** Sonic toothbrush | 20 (?) | 1.12 (?) | 1.04 (?) | -0.08◊ (?) | yes |
|  |  |  | **MTB:** Orthodontic toothbrush | 20 (?) | 1.18 (?) | 1.05 (?) | -0.13◊ (?) | yes |
| **Erbe et al.**  **2019**  6 weeks | The modified Quigley–Hein index (QHPI)+ the Turesky-modified  Quigley–Hein index (TMQH,1970) | Buccal & lingual surfaces | **PTB:** Interactive Oral-B Professional Care 6000 (D36) with Oral-B Precision Clean brush head (EB20) | 30 (28) | 3.926 (0.2672◊) | 2.102 (0.2804◊) | -1.808 (0.2804◊) | yes |
|  |  |  | **MTB:** Oral-B Indicator 35 soft toothbrush | 30 (30) | 3.894 (0.2645◊) | 2.936 (0.2799◊) | -0.974 (0.2799◊) | yes |
| **Farhadian et al.**  **2015**  2 weeks | O’Leary plaque index (O’Leary PI, 1972) | Facial surfaces | **PTB:** Oral-B Vitality toothbrush | 18 (?) | 45.40% (20.91%) | ? (?) | ? (?) | yes |
|  |  |  | **MTB:** Oral-B orthodontic toothbrush | 18 (?) | 59.99% (19.86%) | ? (?) | ? (?) | yes |
| **Silvestrini Biavati et al.**  **2010**  8 weeks | O’Leary plaque index (O’Leary PI, 1972) | ? | **PTB:** Oral-B Professional Care 8500 + Oral-B OBH | 10 (10) | 90.00% (?) | 20.55% (?) | -69.45%◊ (?) | yes |
|  |  |  | **MTB:** Oral-B 35 orthodontic toothbrush | 10 (10) | 93.20% (?) | 24.10% (?) | -69.10%◊ (?) | yes |
| **Sadiq & Badea**  **2009**  8 weeks | Silness & Löe plaque index (PI, 1964) | Labial & buccal surfaces | **PTB:** ? | 27 (?) | 1.32 (?) | 0.31 (?) | -1.01◊ (?) | yes |
|  |  |  | **MTB:** ? | 27 (?) | 1.28 (?) | 0.62 (?) | -0.66◊ (?) | yes |
| **Costa et al.**  **2007**  30 days | The Orthodontic modification of the Silness & Löe plaque index (1964) by Williams et al. (OMPI, 1991), presented in percentage | Buccal surfaces | **PTB 1:** Braun Oral-B 3D Plaque Remover | 21 (21) | 7.60% (?) | 6.28% (?) | -1.32%◊ (?) | no |
|  |  |  | **MTB:** Oral-B Model 30 toothbrush | 21 (21) | 14.89% (?) | 10.39% (?) | -4.50%◊ (?) | no |
| **Kossack & Jost-Brinkmann**  **2005**  4 weeks | Modified Quigley-Hein plaque index (QHPI) | Approximal & buccal surfaces | **PTB:** Water Pik Sonic Speed SR-100E sonic toothbrush | 40 (37) | 0 (0) | ? (?) | -0.228◊ (?) | yes |
|  |  |  | **MTB:** Elmex interX short brush-head toothbrush | 40 (37) | 0 (0) | ? (?) | -0.128◊ (?) | no |
| **Hickman et al.**  **2002**  8 weeks | The Orthodontic modification of the Silness & Löe plaque index (1964) by Williams et al. (OMPI, 1991) | Labial & buccal surfaces | **PTB:** Braun Oral-B Plaque Remover D5 + OBH | 33 (31) | 0.55 (0.26) | 0.46 (0.24) | -0.07 (0.27) | no |
|  |  |  | **MTB:** Reach Compact Head Medium | 30 (29) | 0.58 (0.35) | 0.46 (0.26) | -0.12 (0.26) | yes |
| **Thienpont et al.**  **2001**  4 weeks | The Orthodontic modification of the Silness & Löe plaque index (1964) by Williams et al. (OMPI, 1991) | Mesial, distal and gingival sides of brackets | **PTB 1:** Braun Oral-B 3D Plaque Remover + OBH | 33 (33) | ? (?) | ? (?) | ? (?) | ? |
|  |  |  | **PTB 2:** Philips-Jordan HP 510 + RBH | 33 (33) | ? (?) | ? (?) | ? (?) | ? |
|  |  |  | **MTB 1:** Oral-B Advantage Control Grip | 33 (33) | ? (?) | ? (?) | ? (?) | ? |
|  |  |  | **MTB 2:** Lactona orthodontic brush | 33 (33) | ? (?) | ? (?) | ? (?) | ? |
|  | Quigley-Hein plaque index (QHPI, 1962) | Mesiobuccal, distobuccal, and gingival surfaces (no lingual surfaces) | **PTB 1:** Braun Oral-B 3D Plaque Remover + OBH | 33 (33) | ? (?) | ? (?) | ? (?) | ? |
|  |  |  | **PTB 2:** Philips-Jordan HP 510 + RBH | 33 (33) | ? (?) | ? (?) | ? (?) | ? |
|  |  |  | **MTB 1:** Oral B Advantage Control Grip | 33 (33) | ? (?) | ? (?) | ? (?) | ? |
|  |  |  | **MTB 2:** Lactona orthodontic brush | 33 (33) | ? (?) | ? (?) | ? (?) | ? |
| **Clerehugh et al.**  **1998**  8 weeks | The Orthodontic modification of the Silness & Löe plaque index (1964) by Williams et al. (OMPI, 1991) | Buccal surfaces | **PTB:** Braun Oral-B Plaque Remover (D5) + OBH (OD5) | 41 (37) | 1.47 (0.23) | 1.00 (0.30) | -0.47◊ (0.31◊) | yes |
|  |  |  | **MTB:** Reach Compact Head Medium conventional toothbrush | 43 (42) | 1.40 (0.19) | 1.00 (0.34) | -0.41◊ (0.38◊) | yes |
| **Heasman et al.**  **1998**  4 weeks | Visible plaque index by Ainamo & Bay (VPI, 1975) | Buccal surfaces | **PTB 1:** Braun Oral-B Plaque Remover (D7) + OBH (OD5-1) | 60 (60) | 39% (20%) | 41% (21%) | 2%◊ (21%◊) | ? |
|  |  |  | **PTB 2:** Dental Logic HP550 + RBH (HP5924) | 60 (60) | 39% (20%) | 36% (20%) | -3%◊ (20%◊) | ? |
|  |  |  | **MTB:** Oral-B P35 orthodontic toothbrush | 60 (60) | 39% (20%) | 32% (19%) | -7%◊ (20%◊) | ? |
| **Trimpeneers et al.**  **1997**  2 months | Plaque index brackets  (BPI, 0-3) | Around brackets | **PTB 1:** Interplak | 36 (35◊) | 1.35 (?) | ? (?) | ? (?) | ? |
|  |  |  | **PTB 2:** Philips | 36 (35◊) | 1.26 (?) | ? (?) | ? (?) | ? |
|  |  |  | **PTB 3:** Rotadent with hollow cup-shaped brush | 36 (35◊) | 1.33 (?) | ? (?) | ? (?) | ? |
|  |  |  | **MTB:** Blend-a-Med Dental Plus Junior toothbrush | 36 (35◊) | 1.35 (?) | ? (?) | ? (?) | ? |
|  | Quigley-Hein plaque index (QHPI, 1962) | All surfaces | **PTB 1:** Interplak | 36 (35◊) | 1.10 (?) | ? (?) | ? (?) | ? |
|  |  |  | **PTB 2:** Philips | 36 (35◊) | 1.03 (?) | ? (?) | ? (?) | ? |
|  |  |  | **PTB 3:** Rotadent with hollow cup-shaped brush | 36 (35◊) | 1.18 (?) | ? (?) | ? (?) | ? |
|  |  |  | **MTB:** Blend-a-Med Dental Plus Junior toothbrush | 36 (35◊) | 1.07 (?) | ? (?) | ? (?) | ? |
| **Ho & Niederman**  **1997**  4 weeks | Silness & Löe plaque index (PI, 1964) | 6 sites around teeth | **PTB:** Sonicare sonic toothbrush | 12◊ (?) | 2.65 (0.38) | 1.15 (0.17) | -1.50◊ (0.10) | yes |
|  |  |  | **MTB:** Oral-B P35 | 12◊ (?) | 2.58 (0.33) | 2.33 (0.44) | -0.25◊ (0.10) | no |
| **White**  **1996**  Around 3 months | Hygiene analysis index (HAI) | Facial surfaces | **PTB:** Sonicare | 20◊ (18) | 0.98 (0.08) | 0.80 (0.23) | -0.18◊ (0.20◊) | yes |
|  |  |  | **MTB:** multi-tufted toothbrush | 20◊ (14) | 0.99 (0.02) | 0.93 (0.13) | -0.06◊ (0.12◊) | no |
| **Trombeli et al.**  **1995**  2 weeks | Modified O’Leary plaque index (O’Leary PI) | Buccal surfaces | **PTB:** Interplak | 20 (20) | ? (?) | 25.0% (6.0%) | ? (?) | ? |
|  |  |  | **MTB:** GUM 311 conventional toothbrush | 20 (20) | ? (?) | 41.4% (5.0%) | ? (?) | ? |
| **Wilcoxon et al.**  **1991**  1 month | Modified O’Leary plaque index (O’Leary PI) | Facial & lingual surfaces  Prebrushing | **PTB:** Interplak | 10◊ (?) | 91.9% (7.1%) | 93.3% (6.3%) | 1.4%◊ (6.7%◊) | ? |
|  |  |  | **MTB:** Oral B-15 soft-bristle orthodontic toothbrush | 10◊ (?) | 94.5% (5.5%) | 91.9% (8.3%) | -2.6%◊ (7.3%◊) | ? |
|  |  | Facial & lingual surfaces  Postbrushing | **PTB:** Interplak | 10◊ (?) | 82.0% (11.6%) | 40.8% (17.8%) | -41.2%◊ (15.6%) | yes |
|  |  |  | **MTB:** Oral B-15 soft-bristle orthodontic toothbrush | 10◊ (?) | 84.4% (11.4%) | 76.3% (13.1%) | -8.1%◊ (12.3%) | no |
| **Jackson**  **1991**  >4 weeks | Jackson orthodontic plaque index (JOPI, 1991) | Bracket sides | **PTB:** 2500 arc-shaped vertical movements per minute | 20 (20) | 55.6% (14%) | 48.4% (15.5%) | -7.2%◊ (16.0%◊) | no◊ |
|  |  |  | **MTB:** patients’ own toothbrush | 20 (20) | 55.6% (14%) | 53.6% (11.9%) | -2.0%◊ (10.5%◊) | no◊ |
| **van Venrooy et al.**  **1985**  1 week | Silness & Löe plaque index (PI, 1964) | Buccal & lingual surfaces | **PTB:** Interplak | 24 (24) | ? (?) | ? (?) | ? (?) | ? |
|  |  |  | **MTB:** Oral-B 40 toothbrush | 24 (24) | ? (?) | ? (?) | ? (?) | ? |

◊: calculated by the authors of this review based on the data presented in the selected paper; ?: unknown; SD: standard deviations; MTB: manual toothbrush; PTB: powered toothbrush; OBH: orthodontic brush head; RBH: regular brush head; CBH: CrossAction brush head

**S5B.** Overview of extracted data of **Gingivitis scores** after **the longest follow-up** of the selected studies with various indices and their modifications. Baseline, end-trial measurements and incremental differences are presented as the (adjusted) means and standard deviations (SD) shown in parentheses. Statistically significant changes within groups are presented.

| **Authors (year)**  **Intervention duration** | **Indices used for evaluation** | **Evaluation**  **surfaces** | **Groups** | **Included subjects**  **Baseline (endpoint)** | **Mean (SD)** | | | **Significant**  **within groups** |
| --- | --- | --- | --- | --- | --- | --- | --- | --- |
|  |  |  |  |  | **Baseline** | **Endpoint** | **Difference** |  |
| **Erden & Camcı**  **2023**  12 weeks | Löe & Silness gingival index (GI) | ? | **PTB:** 3D Oral-B Genius 8900 + Oral-B OBH | 20◊ (20◊) | 1.62 (0.19) | 1.29 (0.13) | -0.33◊ (0.17◊) | yes |
|  |  |  | **MTB:** Oral-B Orthodontic brush | 20◊ (20◊) | 1.60 (0.19) | 1.29 (0.11) | -0.31◊ (0.17◊) | yes |
| **Kumar et al.**  **2023**  2 months | Löe & Silness gingival index (GI) | ? | **PTB:** ? | 73 (70) | 1.5 (0.58) | 0.8 (0.43) | -0.7◊ (0.52◊) | yes |
|  |  |  | **MTB:** ? | 71 (70) | 1.4 (0.48) | 1.9 (0.30) | 0.5◊ (0.42◊) | yes |
| **Manphibool et al.**  **2023**  4 weeks | Löe & Silness gingival index (GI) | Bracket sides | **PTB:** LED toothbrush | 24 (23) | 1.49 (0.34◊) | 1.41 (0.34◊) | -0.09 (0.34◊) | no◊ |
|  |  |  | **MTB:** Systema OD orthodontic toothbrush | 24 (23) | 1.40 (0.39◊) | 1.37 (0.34◊) | -0.03 (0.38◊) | no◊ |
| **Mylonopoulou et al.**  **2021**  3 months | Modified Löe & Silness gingival index (MGI) | Labial surfaces (mesial, buccal, distal) | **PTB:** 3D Oral-B Pro-1000 + Oral-B OBH | 40 (40) | 0.62 (0.13) | 0.62 (0.13) | 0.01◊ (0.10◊) | no◊ |
|  |  |  | **MTB:** Oral-B Orthodontic brush | 40 (40) | 0.62 (0.13) | 0.62 (0.10) | 0.00◊ (0.10◊) | no◊ |
| **Bilen et al.**  **2021**  4 months | Silness & Löe gingival index (GI, 1964) | ? | **PTB:** Oral B Interactive 8900 + Oral-B CBH | 18 (18) | 1.07 (0.08) | 0.63 (0.16) | -0.44◊ (0.16) | yes |
|  |  |  | **MTB:** Oral-B Orthodontic brush | 18 (18) | 1.00 (0.24) | 0.75 (0.26) | -0.25◊ (0.14) | yes |
| **Naqvi et al.**  **2020**  8 weeks | Löe & Silness gingival index (GI,1963) | ? | **PTB 1:** ? | 20 (?) | 1.08 (?) | 1.05 (?) | -0.03◊ (?) | no |
|  |  |  | **PTB 2:** Sonic toothbrush | 20 (?) | 1.09 (?) | 1.05 (?) | -0.04◊ (?) | yes |
|  |  |  | **MTB:** Orthodontic toothbrush | 20 (?) | 1.10 (?) | 1.06 (?) | -0.04◊ (?) | no |
| **Farhadian et al.**  **2015**  2 weeks | Löe & Silness gingival index (GI, 1963) | Facial surfaces | **PTB** Oral-B Vitality toothbrush | 18 (?) | 1.82 (0.28) | ? (?) | ? (?) | yes |
|  |  |  | **MTB:** Oral-B orthodontic toothbrush | 18 (?) | 1.83 (0.18) | ? (?) | ? (?) | yes |
| **Sadiq & Badea**  **2009**  8 weeks | Gingival index by Ainamo et al. (AGI, 1982) | ? | **PTB:** ? | 27 (?) | 1.82 (?) | 0.60 (?) | -1.22◊ (?) | yes |
|  |  |  | **MTB:** ? | 27 (?) | 1.77 (?) | 0.98 (?) | -0.79◊ (?) | yes |
| **Hickman et al.**  **2002**  8 weeks | Löe gingival index (GI, 1967) | ? | **PTB:** Braun Oral-B Plaque Remover D5 + OBH | 33 (31) | 1.17 (0.24) | 1.12 (0.18) | -0.04 (0.17) | no |
|  |  |  | **MTB:** Reach Compact Head Medium | 30 (29) | 1.18 (0.28) | 1.12 (0.23) | -0.06 (0.18) | no |
| **Thienpont et al.**  **2001**  4 weeks | Lobene modified gingival index (LMGI, 1986) | No lingual surfaces | **PTB 1:** Braun Oral-B 3D Plaque Remover + OBH | 33 (33) | ? (?) | ? (?) | ? (?) | ? |
|  |  |  | **PTB 2:** Philips-Jordan HP 510 + RBH | 33 (33) | ? (?) | ? (?) | ? (?) | ? |
|  |  |  | **MTB 1:** Oral B Advantage Control Grip | 33 (33) | ? (?) | ? (?) | ? (?) | ? |
|  |  |  | **MTB 2:** Lactona orthodontic brush | 33 (33) | ? (?) | ? (?) | ? (?) | ? |
| **Clerehugh et al. 1998**  8 weeks | Löe gingival index (GI, 1967) | Buccal surfaces | **PTB:** Braun Oral-B Plaque Remover (D5) + OBH (OD5) | 41 (37) | 1.68 (0.17) | 1.67 (0.18) | -0.02◊ (0.17◊) | no |
|  |  |  | **MTB:** Reach Compact Head Medium | 43 (42) | 1.71 (0.16) | 1.70 (0.17) | -0.01◊ (0.17◊) | no |
| **Trimpeneers et al.**  **1997**  2 months | Lobene modified gingival index (LMGI, 1986) | All surfaces | **PTB 1:** Interplak | 36 (35◊) | 1.36 (?) | ? (?) | ? (?) | ? |
|  |  |  | **PTB 2:** Philips | 36 (35◊) | 1.38 (?) | ? (?) | ? (?) | ? |
|  |  |  | **PTB 3:** Rotadent with hollow cup-shaped brush | 36 (35◊) | 1.40 (?) | ? (?) | ? (?) | ? |
|  |  |  | **MTB:** Blend-a-Med Dental Plus Junior toothbrush | 36 (35◊) | 1.38 (?) | ? (?) | ? (?) | ? |
| **Ho & Niederman**  **1997**  4 weeks | Löe & Silness gingival index (GI, 1963) | 6 sites around teeth | **PTB:** Sonicare sonic toothbrush | 12◊ (?) | 2.00 (0.00) | 1.42 (0.27) | -0.58◊ (0.08) | yes |
|  |  |  | **MTB:** Oral-B P35 | 12◊ (?) | 2.02 (0.07) | 1.96 (0.14) | -0.06◊ (0.05) | no |
| **Wilcoxon et al.**  **1991**  1 month | Ramfjord gingival index (RGI, 1959) | All surfaces | **PTB:** Interplak | 10◊ (?) | 55.7% (13.7%) | 54.6% (14.0%) | -1.1%◊ (13.9%◊) | no |
|  |  |  | **MTB:** Oral B-15 soft-bristle orthodontic toothbrush | 10◊ (?) | 60.8% (12.5%) | 61.2% (8.7%) | 0.4%◊ (11.1%◊) | ? |
| **Jackson**  **1991**  >4 weeks | Modified Löe & Silness gingival index (MGI) | Labial surfaces (mesial, buccal, distal) | **PTB:** 2500 arc-shaped vertical movements per minute | 20 (20) | 0.90 (0.32) | 0.53 (0.33) | -0.37◊ (0.24◊) | yes ◊ |
|  |  |  | **MTB:** patients’ own toothbrush | 20 (20) | 0.90 (0.32) | 0.59 (0.31) | -0.32◊ (0.24◊) | yes ◊ |

◊: calculated by the authors of this review based on the data presented in the selected paper; ?: unknown; SD: standard deviations; MTB: manual toothbrush; PTB: powered toothbrush; OBH: orthodontic brush head; RBH: regular brush head; CBH: CrossAction brush head

**S5C.** Overview of extracted data of **Gingival Bleeding scores** after **the longest follow-up** of the selected studies with various indices and their modifications. Baseline, end-trial measurements and incremental differences are presented as the (adjusted) means and standard deviations (SD) shown in parentheses. Statistically significant changes within groups are presented.

| **Authors (year)**  **Intervention duration** | **Indices used for evaluation** | **Evaluation**  **surfaces** | **Groups** | **Included subjects**  **Baseline (endpoint)** | **Mean (SD)** | | | **Significant**  **within groups** |
| --- | --- | --- | --- | --- | --- | --- | --- | --- |
|  |  |  |  |  | **Baseline** | **Endpoint** | **Difference** |  |
| **Erden & Camcı 2023**  12 weeks | Bleeding on probing (BOP) | ? | **PTB:** 3D Oral-B Genius 8900 + Oral-B OBH | 20◊ (20◊) | 59.51% (19.74%) | 28.75% (13.42%) | -30.76%◊ (17.46%◊) | yes |
|  |  |  | **MTB:** Oral-B Orthodontic brush | 20◊ (20◊) | 59.69% (19.41%) | 29.17% (11.01%) | -30.52%◊ (16.86%◊) | yes |
| **Kumar et al.**  **2023**  2 months | Modified papillary bleeding index (MPBI) | ? | **PTB:** ? | 73 (70) | 1.1 (0.33) | 0.6 (0.52) | -0.5◊ (0.46◊) | no |
|  |  |  | **MTB:** ? | 71 (70) | 1.2 (0.86) | 1.2 (0.51) | 0◊ (0.75◊) | yes |
| **Mylonopoulou et al.**  **2021**  3 months | The modified simplified gingival index by Lindhe. (MGI-S, 1984) | Labial surfaces | **PTB:** 3D Oral-B Pro-1000 + Oral-B OBH | 40 (40) | 0.92 (0.16) | 0.91 (0.15) | -0.01◊ (0.14◊) | no◊ |
|  |  |  | **MTB:** Oral-B Orthodontic brush | 40 (40) | 0.91 (0.14) | 0.92 (0.14) | 0.01◊ (0.12◊) | no◊ |
| **Bilen et al.**  **2021**  4 months | Bleeding on probing (BOP) | ? | **PTB:** Oral B Interactive 8900 + Oral-B CBH | 18 (18) | 22.78% (6.69%) | 10.55% (6.39%) | -12.22%◊ (7.32%) | yes |
|  |  |  | **MTB:** Oral-B Orthodontic brush | 18 (18) | 21.67% (6.42%) | 13.89% (7.39%) | -7.78%◊ (4.28%) | yes |
| **Naqvi et al.**  **2020**  8 weeks | Eastman interdental bleeding index by Caton & Polson (EIBI,1985) | ? | **PTB 1:** ? | 20 (?) | 1.19 (?) | 0.08 (?) | -1.11◊ (?) | no |
|  |  |  | **PTB 2:** Sonic toothbrush | 20 (?) | 1.20 (?) | 0.07 (?) | -1.13◊ (?) | yes |
|  |  |  | **MTB:** Orthodontic toothbrush | 20 (?) | 1.22 (?) | 0.12 (?) | -1.10◊ (?) | no |
| **Farhadian et al.**  **2015**  2 weeks | Bleeding on probing (BOP) | Facial surfaces | **PTB** Oral-B Vitality toothbrush | 18 (?) | 51.86% (25.72%) | ? (?) | ? (?) | yes |
|  |  |  | **MTB:** Oral-B orthodontic toothbrush | 18 (?) | 50.97% (14.91%) | ? (?) | ? (?) | yes |
| **Silvestrini Biavati et al.**  **2010**  8 weeks | Gingival bleeding index by Ainamo and Bay (BI, 1975) | ? | **PTB:** Oral-B Professional Care 8500 + Oral-B OBH | 10 (10) | 18.10% (?) | 0.70% (?) | -16.40%◊ (?) | yes |
|  |  |  | **MTB:** Oral-B 35 orthodontic toothbrush | 10 (10) | 12.10% (?) | 1.30% (?) | -10.80%◊ (?) | yes |
| **Sadiq & Badea**  **2009**  8 weeks | Eastman interdental bleeding index by Caton & Polson (EIBI,1985) | Interdental surfaces | **PTB:** ? | 27 (?) | 47.9% (?) | 22.2% (?) | -25.7%◊ (?) | yes |
|  |  |  | **MTB:** ? | 27 (?) | 49.5% (?) | 37.8% (?) | -11.7%◊ (?) | yes |
| **Costa et al.**  **2007**  30 days | Löe and Silness gingival index for bleeding (LSBI, 1963), presented in percentage | Buccal surfaces | **PTB:** Braun Oral-B 3D Plaque Remover | 21 (21) | 11.15% (?) | 15.51% (?) | 4.36%◊ (?) | no |
|  |  |  | **MTB:** Oral-B Model 30 toothbrush | 21 (21) | 17.22% (?) | 15.22% (?) | -2.00%◊ (?) | no |
| **Kossack & Jost-Brinkmann**  **2005**  4 weeks | Papillary bleeding index by Mühlemann (PBI, 1977) | mesial-approximal and distal-approximal | **PTB:** Water Pik Sonic Speed sonic toothbrush SR-100E | 40 (37) | 0 (0) | ? (?) | -0.004◊ (?) | no |
|  |  |  | **MTB:** elmex interX short brush-head toothbrush | 40 (37) | 0 (0) | ? (?) | 0.006◊ (?) | no |
| **Hickman et al.**  **2002**  8 weeks | Eastman interdental bleeding index by Caton & Polson (EIBI, 1985) | Interdental surfaces | **PTB:** Braun Oral-B Plaque Remover D5 + OBH | 33 (31) | 53.0% (22.3%) | 43.4% (20.6%) | -8.6% (20.9%) | yes |
|  |  |  | **MTB:** Reach Compact Head Medium | 30 (29) | 47.3% (19.3%) | 44.0% (20.1%) | -3.6% (19.0%) | no |
| **Thienpont et al.**  **2001**  4 weeks | Bleeding on probing (BOP) | No lingual surfaces | **PTB 1:** Braun Oral-B 3D Plaque Remover + OBH | 33 (33) | ? (?) | ? (?) | ? (?) | ? |
|  |  |  | **PTB 2:** Philips-Jordan HP 510 + RBH | 33 (33) | ? (?) | ? (?) | ? (?) | ? |
|  |  |  | **MTB 1:** Oral B Advantage Control Grip | 33 (33) | ? (?) | ? (?) | ? (?) | ? |
|  |  |  | **MTB 2:** Lactona orthodontic brush | 33 (33) | ? (?) | ? (?) | ? (?) | ? |
| **Clerehugh et al.**  **1998**  8 weeks | Eastman interdental bleeding index by Caton & Polson (EIBI, 1985) | Interdental surfaces | **PTB:** Braun Oral-B Plaque Remover (D5) with + OBH (OD5) | 41 (37) | 0.74 (0.15) | 0.65 (0.17) | -0.10◊ (0.31◊) | yes |
|  |  |  | **MTB:** Reach Compact Head Medium | 43 (42) | 0.69 (0.17) | 0.67 (0.19) | -0.02◊ (0.25◊) | no |
| **Heasman et al.**  **1998**  4 weeks | Gingival bleeding index by Ainamo and Bay (BI, 1975) | 4 sites around teeth (mesial, buccal, lingual, distal) | **PTB 1:** Braun Oral-B Plaque Remover (D7) + OBH (OD5-1) | 60 (60) | 26% (16%) | 17% (12%) | -9%◊ (14%◊) | ? |
|  |  |  | **PTB 2:** Dental Logic HP550 + OBH (HP5924) | 60 (60) | 26% (16%) | 18% (10%) | -8%◊ (14%◊) | ? |
|  |  |  | **MTB:** Oral-B P35 orthodontic toothbrush | 60 (60) | 26% (16%) | 17% (10%) | -9%◊ (14%◊) | ? |
| **Trimpeneers et al.**  **1997**  2 months | Bleeding on probing (BOP, 0-2) | 6 sites around teeth | **PTB 1:** Interplak | 36 (35◊) | 0.45 (?) | ? (?) | ? (?) | ? |
|  |  |  | **PTB 2:** Philips | 36 (35◊) | 0.44 (?) | ? (?) | ? (?) | ? |
|  |  |  | **PTB 3:** Rotadent with hollow cup-shaped brush | 36 (35◊) | 0.44 (?) | ? (?) | ? (?) | ? |
|  |  |  | **MTB:** Blend-a-Med Dental Plus Junior toothbrush | 36 (35◊) | 0.47 (?) | ? (?) | ? (?) | ? |
| **Ho & Niederman**  **1997**  4 weeks | Bleeding on probing (BOP) | 6 sites around teeth | **PTB:** Sonicare sonic toothbrush | 12◊ (?) | 78.12% (11.94%) | 24.53% (10.64%) | -53.59%◊ (11.35%◊) | yes |
|  |  |  | **MTB:** Oral-B P35 | 12◊ (?) | 70.13% (8.12%) | 64.71% (11.16%) | -5.42%◊ (9.99%◊) | no |
| **White**  **1996**  Around 3 months | Modified papillary bleeding index (MPBI) | Mesial papillae between the anterior teeth | **PTB:** Sonicare | 20◊ (18) | 1.74 (0.70) | 1.34 (0.53) | -0.40◊ (0.63◊) | yes |
|  |  |  | **MTB:** multi-tufted toothbrush | 20◊ (14) | 1.57 (0.42) | 1.81 (0.24) | 0.24◊ (0.37◊) | yes |

◊: calculated by the authors of this review based on the data presented in the selected paper; ?: unknown; SD: standard deviations; MTB: manual toothbrush; PTB: powered toothbrush; OBH: orthodontic brush head; RBH: regular brush head; CBH: CrossAction brush head

**S6A.** Forrest Plots of the overall meta-analysis for **Plaque scores,** subgroup meta-analyses for the same plaque index, subgroup meta-analyses for plaque scores based on the same action mode of powered toothbrushes, and subgroup meta-analyses based on the same plaque index and action mode of powered toothbrushes after the longest follow up. SMD or MD are presented for the baseline, end-trial and incremental difference scores using a random- effects model.

●Overall meta-analysis on different indices*: Quigley-Hein plaque index (1962) and its other modifications (QHPI), the Turesky-modified Quigley–Hein index (TMQH, 1970), the orthodontic modification of the Silness & Löe plaque index (1964) by Williams et al. (OMPI, 1991), O’Leary plaque index and its modification (O’Leary PI), plaque index by Silness & Löe (PI, 1964), visible plaque index by Ainamo & Bay (VPI, 1975), hygiene analysis index (HAI), Jackson orthodontic plaque index (JOPI, 1991)

**S6A-1**.Forrest Plots at baseline


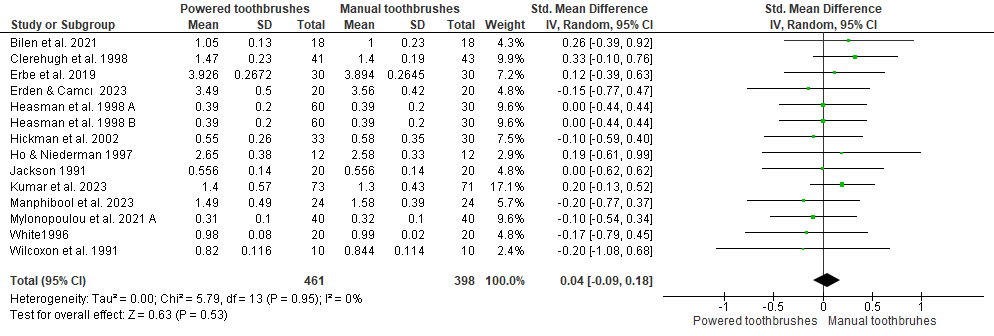


**S6A-2**.Forrest Plots of end-trial scores


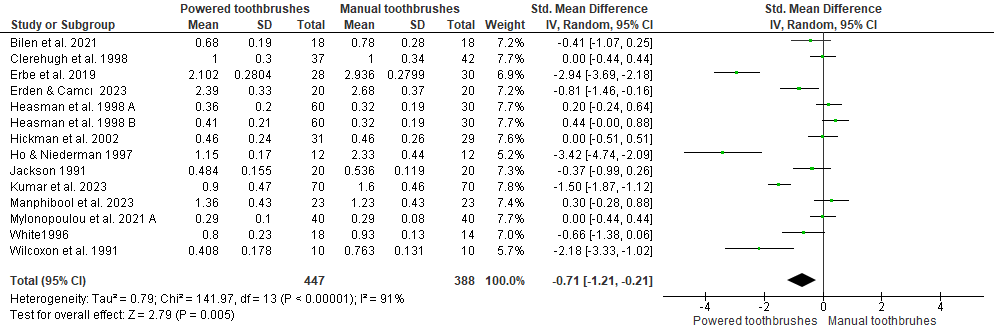


**S6A-3**.Forrest Plots of incremental difference


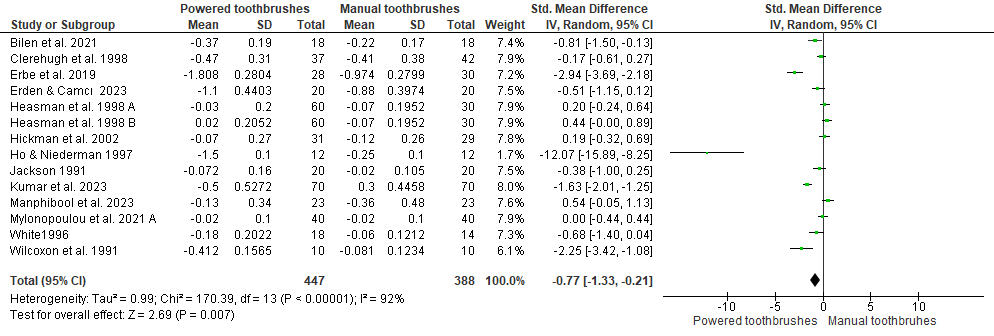


●Subgroup meta-analysis on the same index 1: OMPI

**S6A-4**.Forrest Plots at baseline


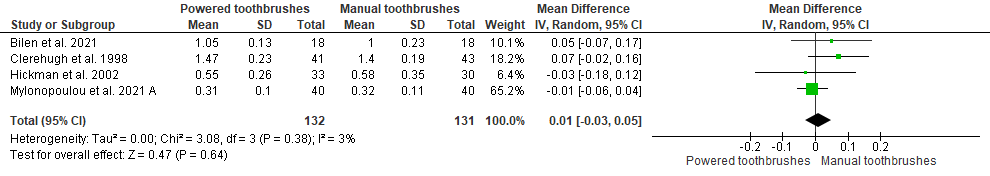


**S6A-5**.Forrest Plots of end-trial scores


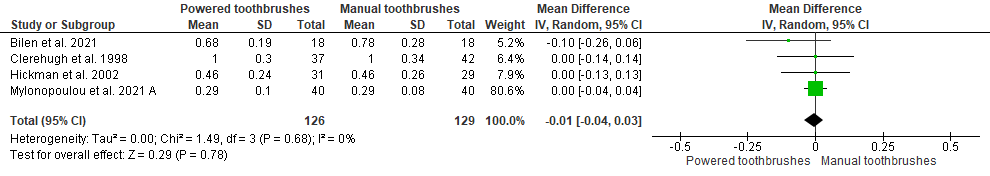


**S6A-6**.Forrest Plots of incremental difference


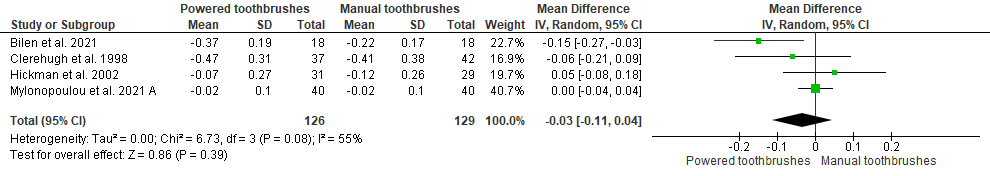


●Subgroup meta-analysis on the same index 2: QHPI/TMQH

**S6A-7**.Forrest Plots at baseline


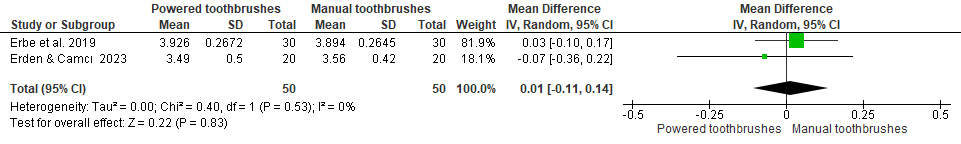


**S6A-8**.Forrest Plots of end-trial scores

**
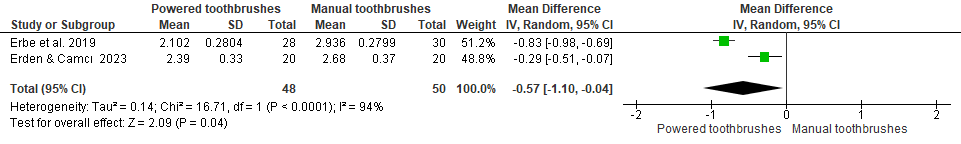
**

**S6A-9**.Forrest Plots of incremental difference

**
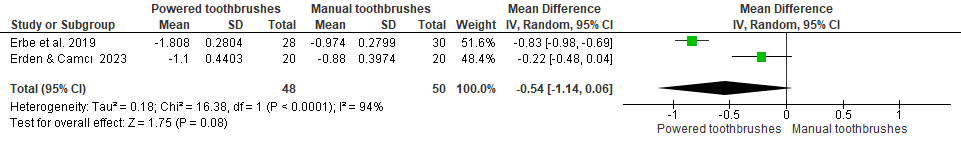
**

●Subgroup meta-analysis on the same index 3: O’Leary PI

**S6A-10**.Forrest Plots at baseline


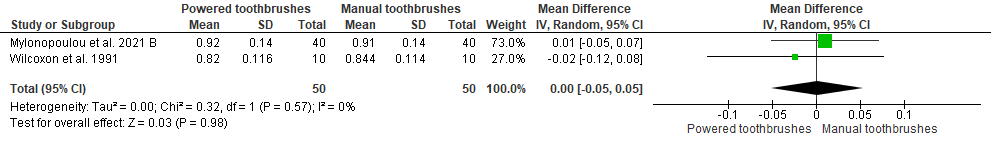


**S6A-11**.Forrest Plots of end-trial scores


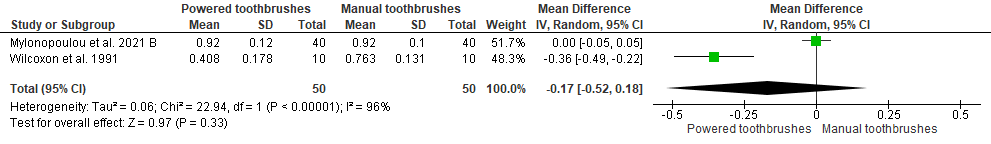


**S6A-12**.Forrest Plots of incremental difference


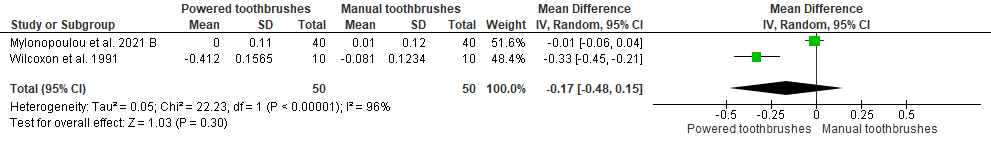


●Subgroup meta-analysis on the same index 4: PI

**S6A-13**.Forrest Plots at baseline


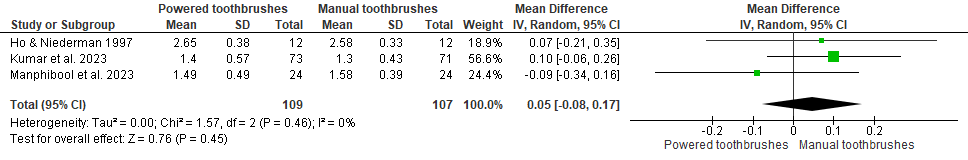


**S6A-14**.Forrest Plots of end-trial scores


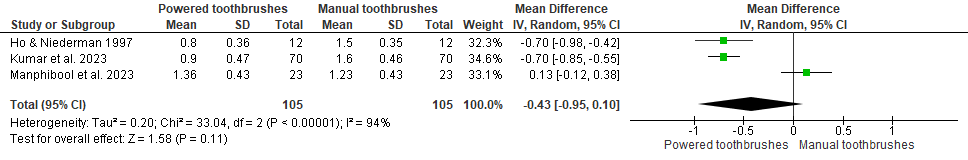


**S6A-15**.Forrest Plots of incremental difference


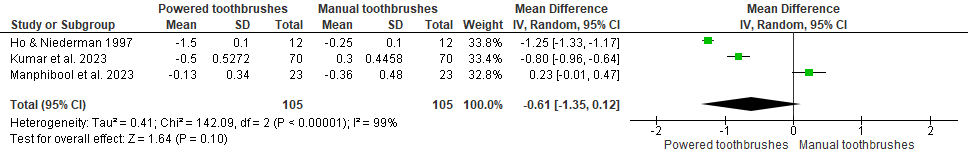


●Subgroup meta-analysis on the same index 5: VPI

**S6A-16**.Forrest Plots at baseline


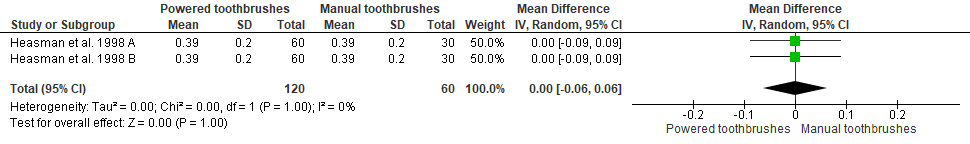


**S6A-17**.Forrest Plots of end-trial scores


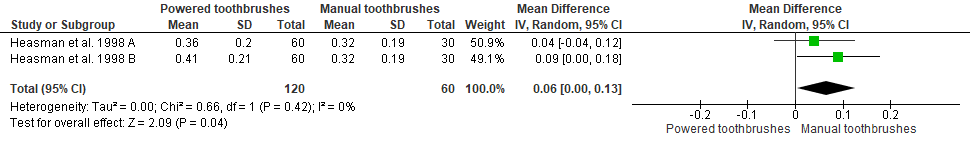


**S6A-18**.Forrest Plots of incremental difference


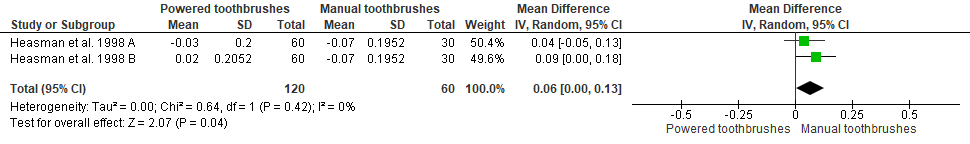


●Subgroup meta-analysis on the same action model 1: Oscillating-rotating-pulsating action (ORP)/Oscillating-rotating action (OR)

**S6A-19**.Forrest Plots at baseline


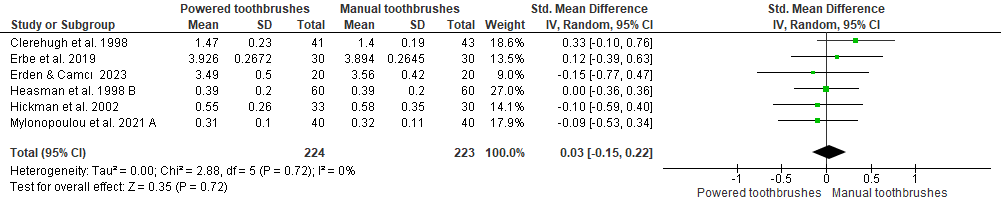


**S6A-20**.Forrest Plots of end-trial scores


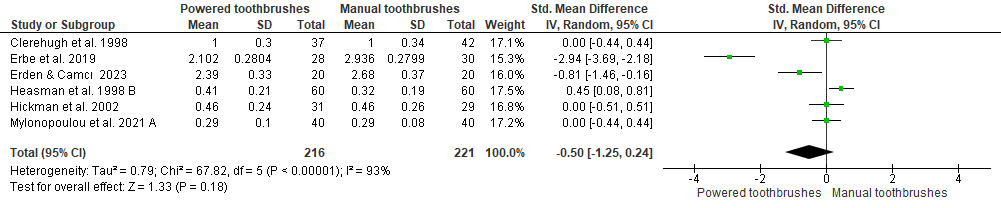


**S6A-21**.Forrest Plots of incremental difference

**
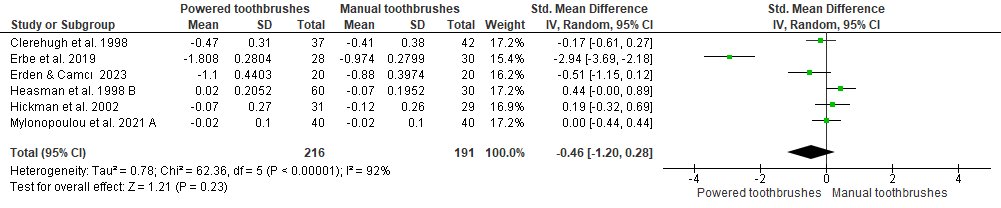
**

●Subgroup meta-analysis on the same action model 2: ORP

**S6A-22**.Forrest Plots at baseline


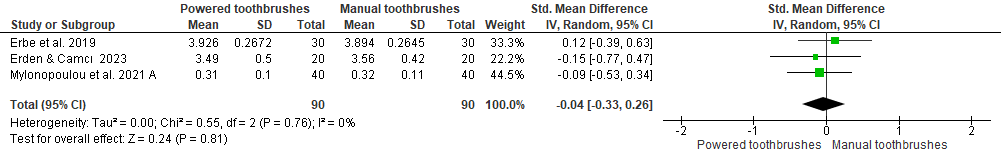


**S6A-23**.Forrest Plots of end-trial scores


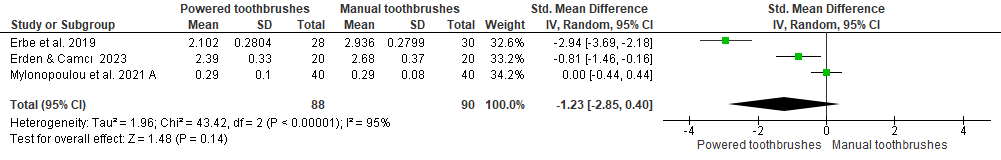


**S6A-24**.Forrest Plots of incremental difference

**
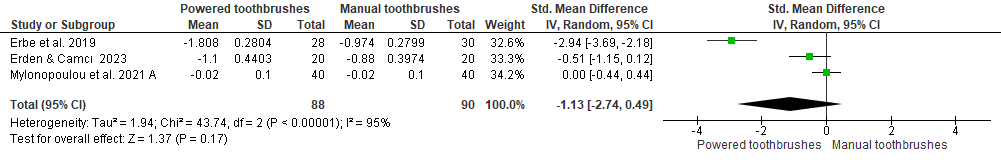
**

●Subgroup meta-analysis on the same action model 3: OR

**S6A-25**.Forrest Plots at baseline


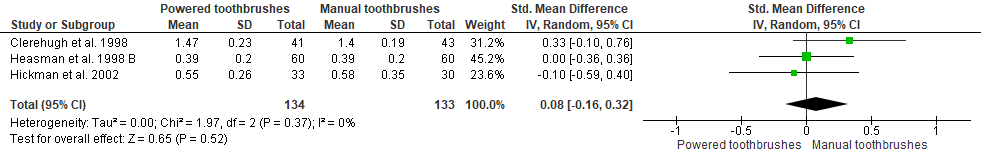


**S6A-26**.Forrest Plots of end-trial scores


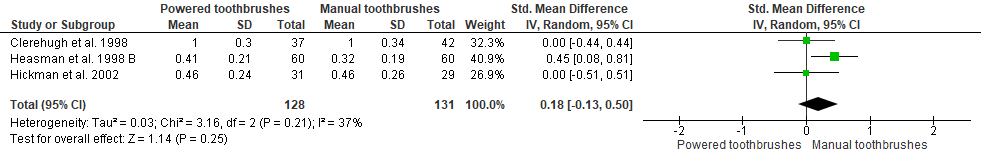


**S6A-27**.Forrest Plots of incremental difference

**
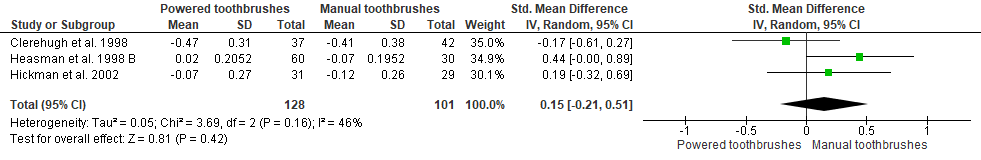
**

●Subgroup meta-analysis on the same action model 4: Counter-rotational (CR)

**S6A-28**.Forrest Plots at baseline


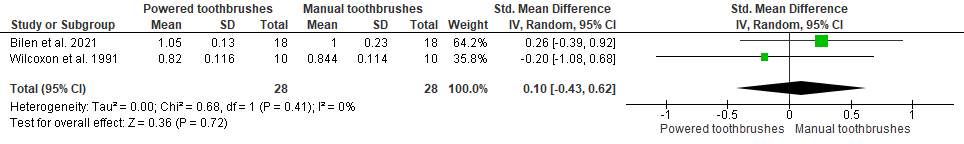


**S6A-29**.Forrest Plots of end-trial scores


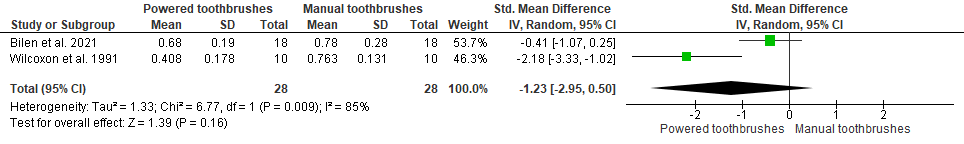


**S6A-30**.Forrest Plots of incremental difference


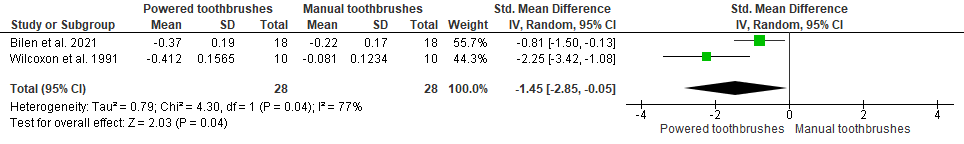


●Subgroup meta-analysis on action model 5: Sonic

**S6A-31**.Forrest Plots at baseline


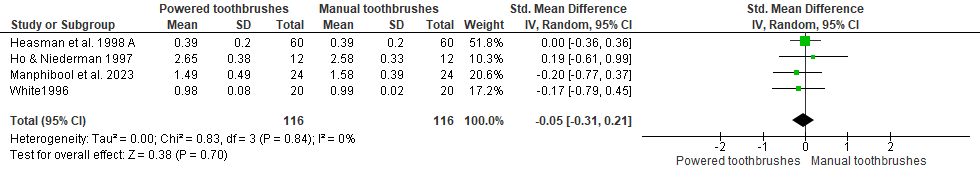


**S6A-32**.Forrest Plots of end-trial scores


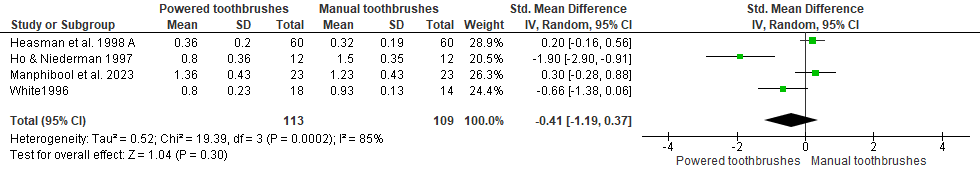


**S6A-33**.Forrest Plots of incremental difference


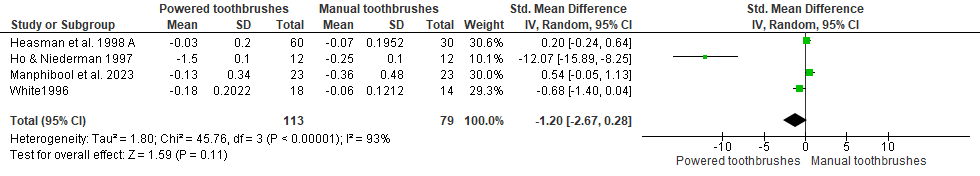


●Subgroup meta-analysis on the same index and action model 1: ORP/OR on OMPI

**S6A-34**.Forrest Plots at baseline


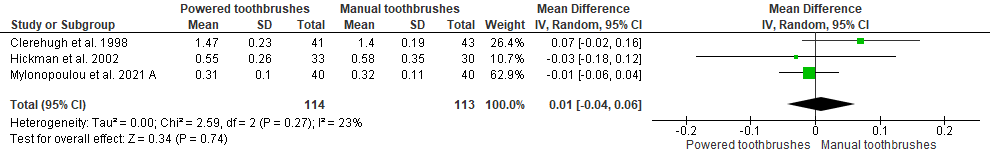


**S6A-35**.Forrest Plots of end-trial scores


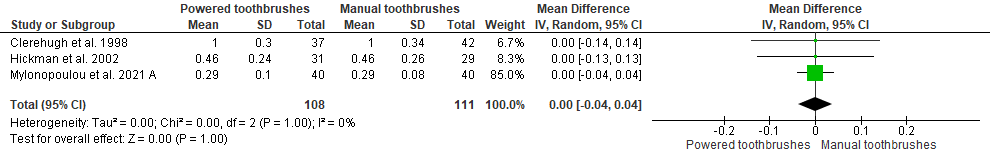


**S6A-36**.Forrest Plots of incremental difference

**
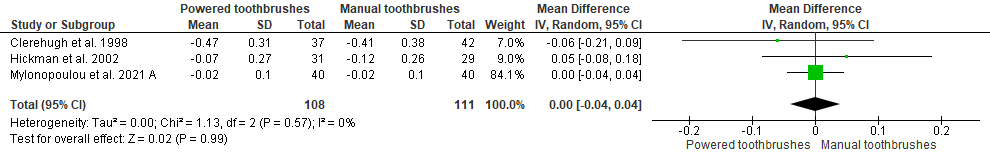
**

●Subgroup meta-analysis on the same index and action model 2: ORP on QHPI & TMQH

**S6A-37**.Forrest Plots at baseline


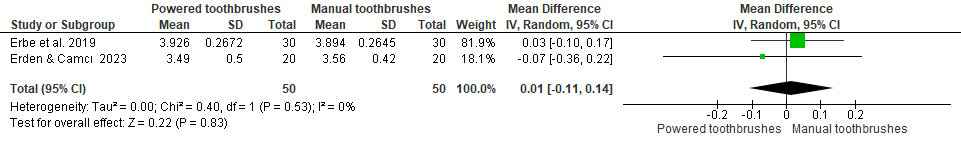


**S6A-38**.Forrest Plots of end-trial scores

**
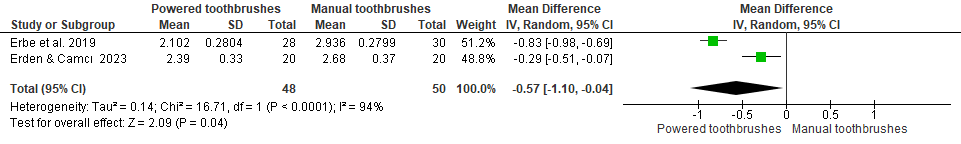
**

**S6A-39**.Forrest Plots of incremental difference

**
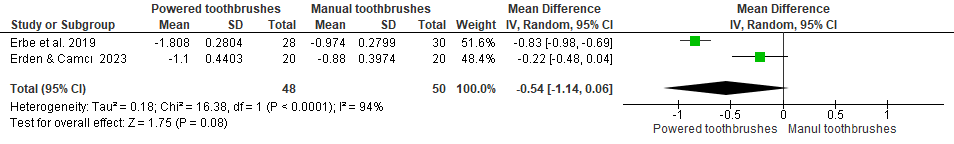
**

Subgroup meta-analysis on the same index and action model 3: OR on OMPI

**S6A-40**.Forrest Plots at baseline


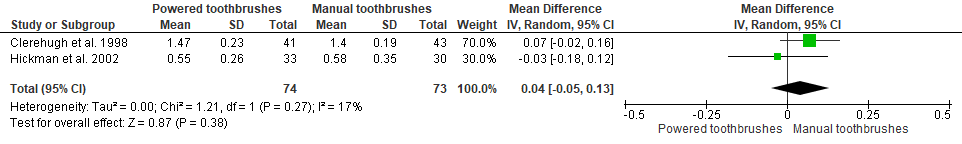


**S6A-41**.Forrest Plots of end-trial scores


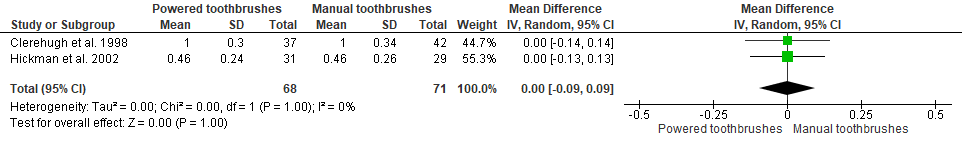


**S6A-42**.Forrest Plots of incremental difference

**
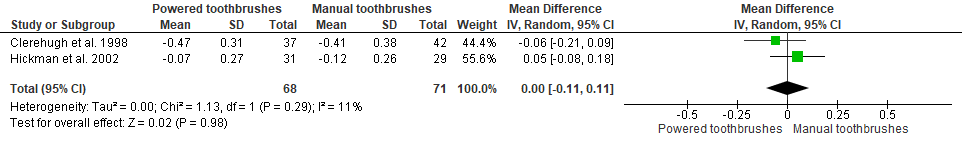
**

Subgroup meta-analysis on the same index and action model 4: Sonic on PI

**S6A-43**.Forrest Plots at baseline


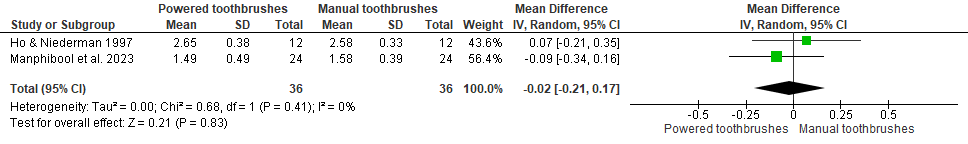


**S6A-44**.Forrest Plots of end-trial scores


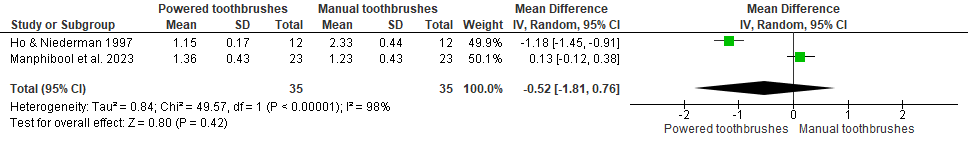


**S6A-45**.Forrest Plots of incremental difference

**
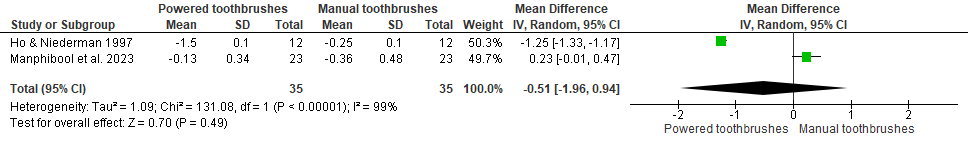
**

**S6B.** Forrest Plots of the overall meta-analysis for **Gingivitis scores,** subgroup meta-analyses for the same gingival index, subgroup meta-analyses for gingivitis scores based on the same action mode of powered toothbrushes, and subgroup meta-analyses based on the same gingival index and action mode of powered toothbrushes after the longest follow up. SMD or MD are presented for the baseline, end-trial and incremental difference scores using a random-effects model.

●Overall meta-analysis on different indices: Löe & Silness gingival index (GI, 1963&1964&1967), Ramfjord gingival index (RGI, 1959)

**S6B-1**.Forrest Plots at baseline


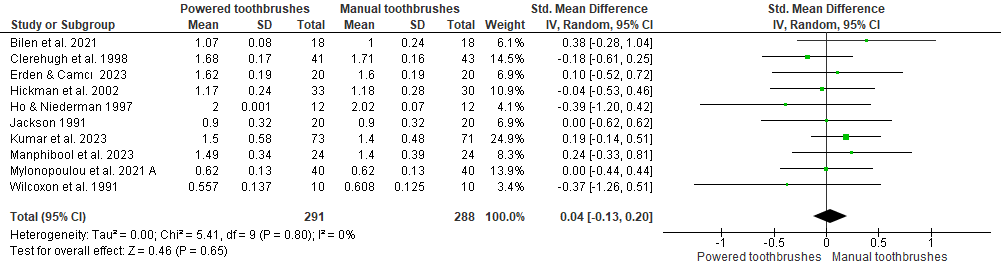


**S6B-2**.Forrest Plots of end-trial scores


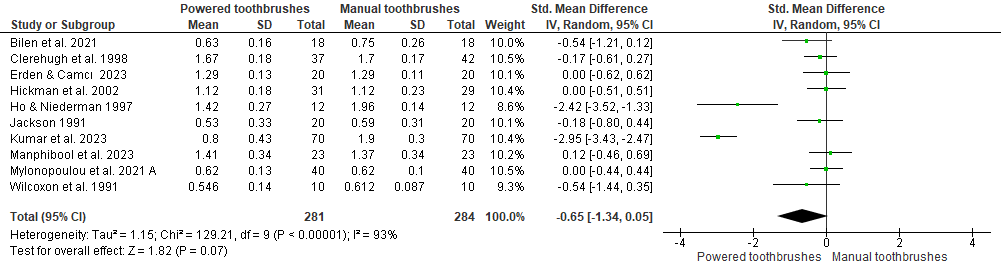


**S6B-3**.Forrest Plots of incremental difference


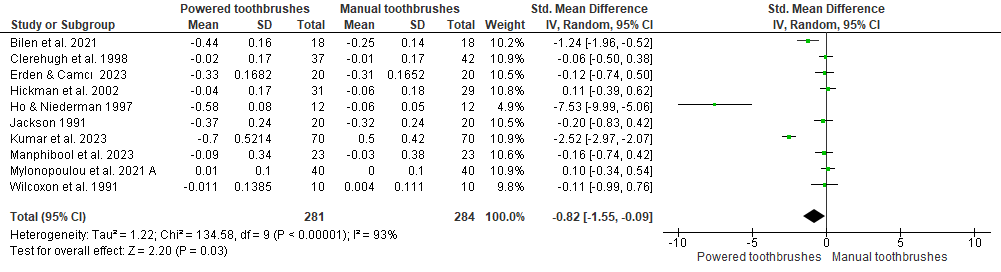


●Subgroup meta-analysis on the same index: GI

**S6B-4**.Forrest Plots at baseline


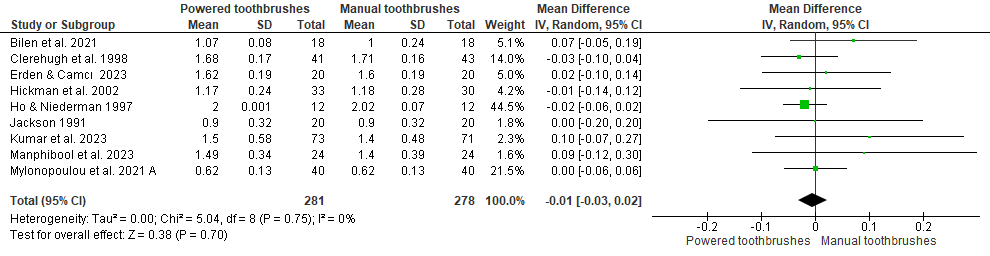


**S6B-5**.Forrest Plots of end-trial scores


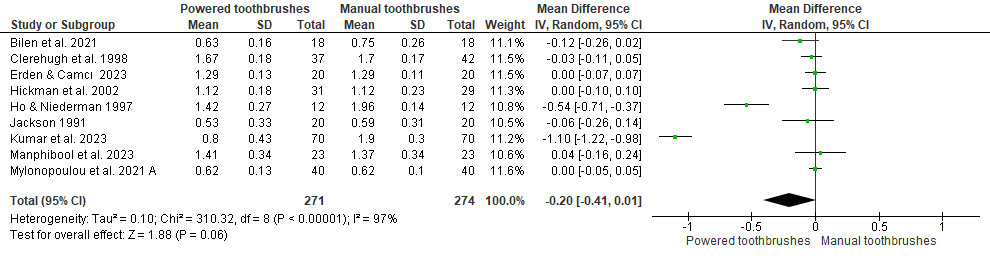


**S6B-6**.Forrest Plots of incremental difference


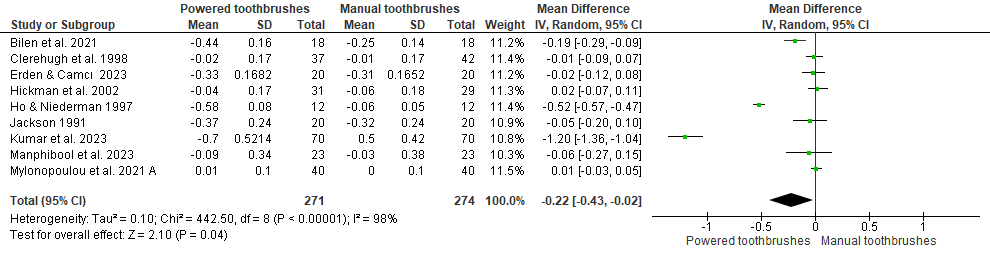


●Subgroup meta-analysis on the same action model 1: ORP/OR

**S6B-7**.Forrest Plots at baseline


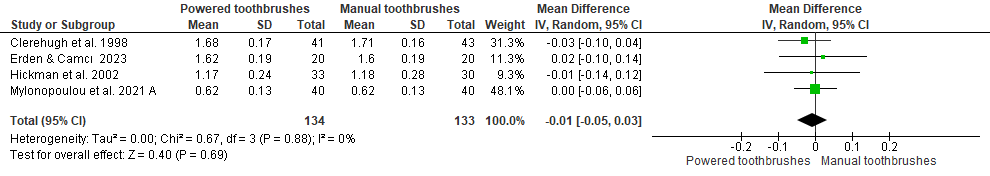


**S6B-8**.Forrest Plots of end-trial scores


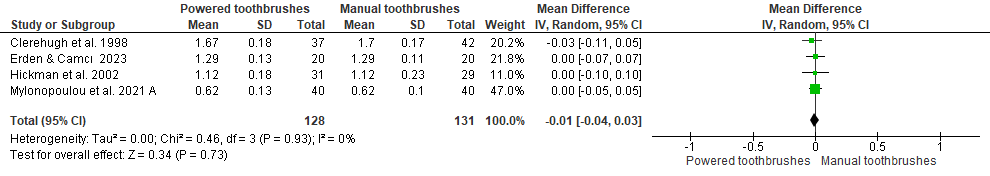


**S6B-9**.Forrest Plots of incremental difference

**
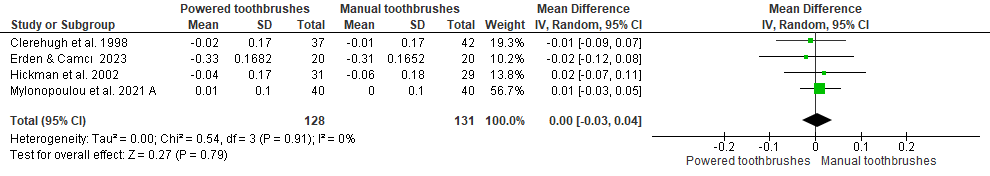
**

●Subgroup meta-analysis on the same action model 2: ORP

**S6B-10**.Forrest Plots at baseline


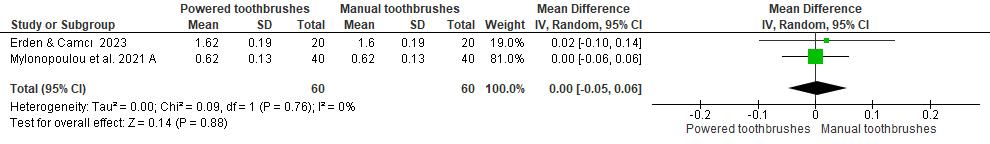


**S6B-11**.Forrest Plots of end-trial scores


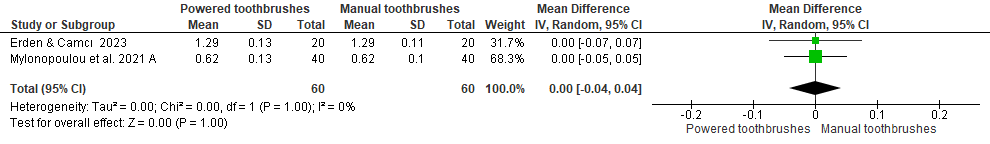


**S6B-12**.Forrest Plots of incremental difference


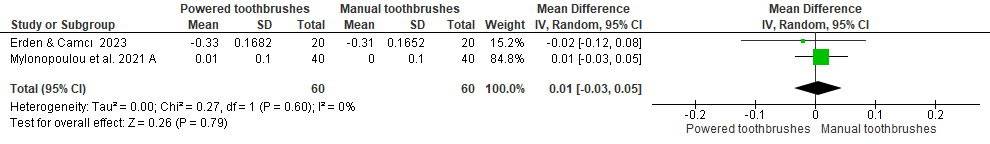


●Subgroup meta-analysis on the same action model 3: OR

**S6B-13**.Forrest Plots at baseline


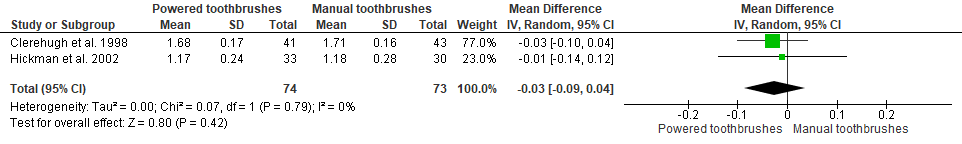


**S6B-14**.Forrest Plots of end-trial scores


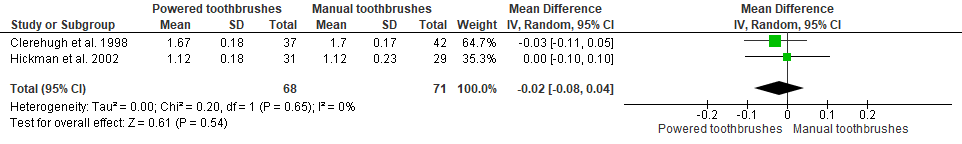


**S6B-15**.Forrest Plots of incremental difference

**
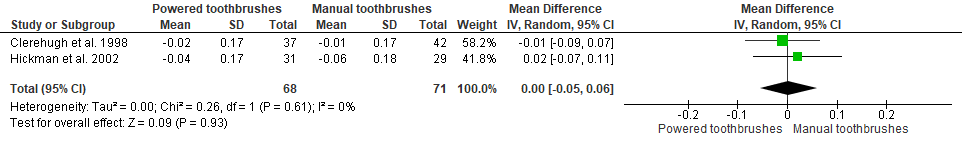
**

●Subgroup meta-analysis on the same action model 4: CR

**S6B-16**.Forrest Plots at baseline


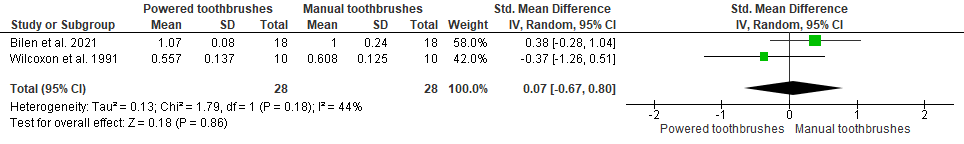


**S6B-17**.Forrest Plots of end-trial scores

**
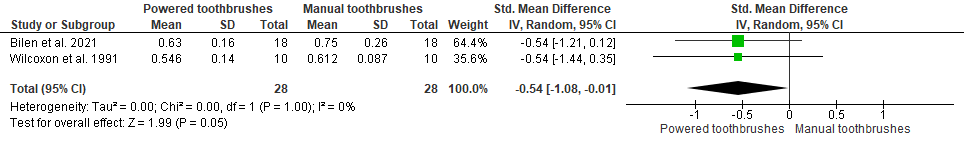
**

**S6B-18**.Forrest Plots of incremental difference

**
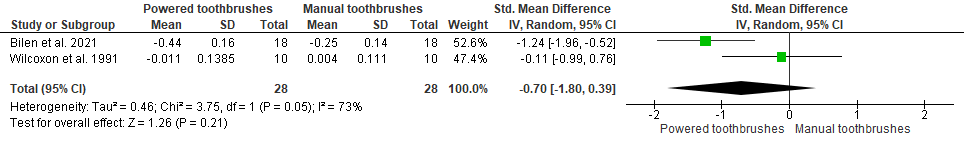
**

●Subgroup meta-analysis on the same action model 5: Sonic

**S6B-19**.Forrest Plots at baseline


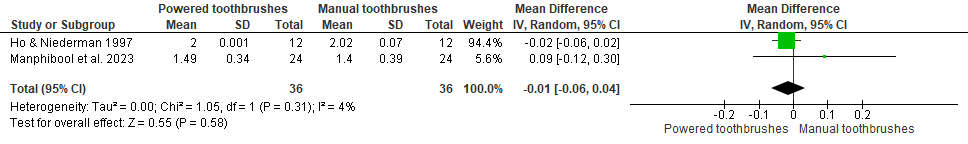


**S6B-20**.Forrest Plots of end-trial scores


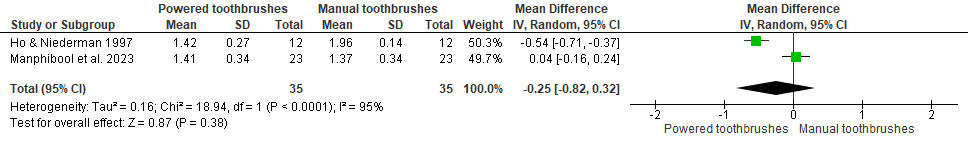


**S6B-21**.Forrest Plots of incremental difference


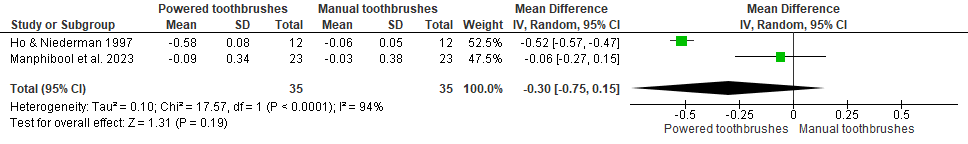


●Subgroup meta-analysis on the same index and action model 1: ORP/OR on GI

**S6B-22**.Forrest Plots at baseline


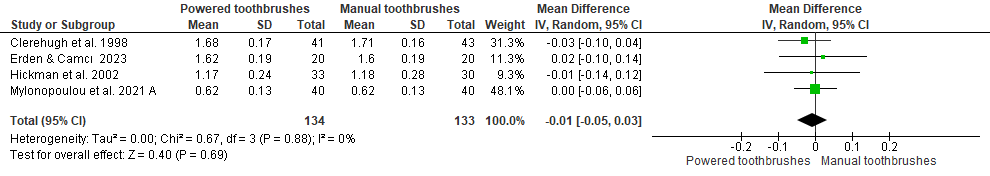


**S6B-23**.Forrest Plots of end-trial scores


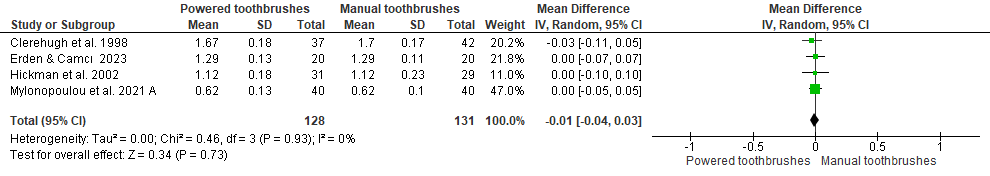


**S6B-24**.Forrest Plots of incremental difference

**
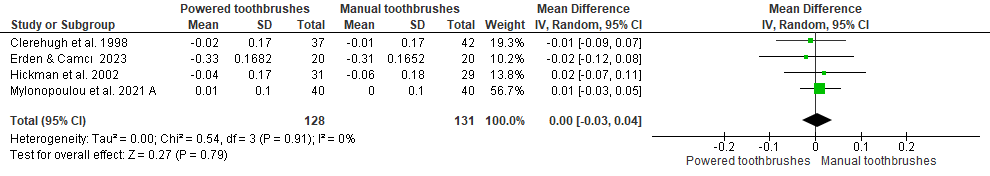
**

●Subgroup meta-analysis on the same index and action model 2: ORP on GI

**S6B-25**.Forrest Plots at baseline


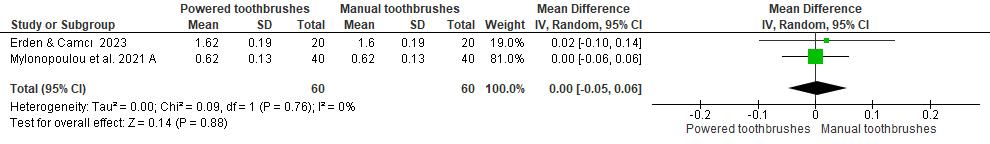


**S6B-26**.Forrest Plots of end-trial scores


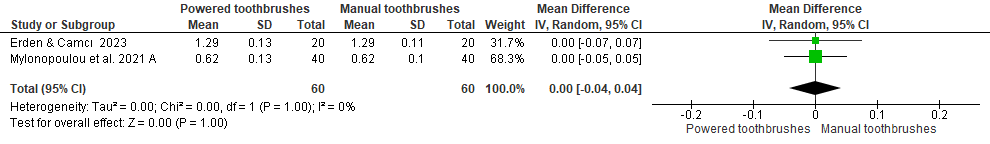


**S6B-27**.Forrest Plots of incremental difference


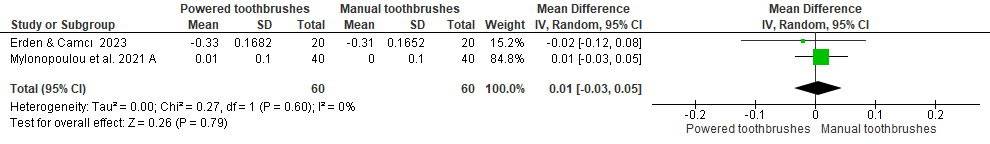


●Subgroup meta-analysis on the same index and action model 3: OR on GI

**S6B-28**.Forrest Plots at baseline


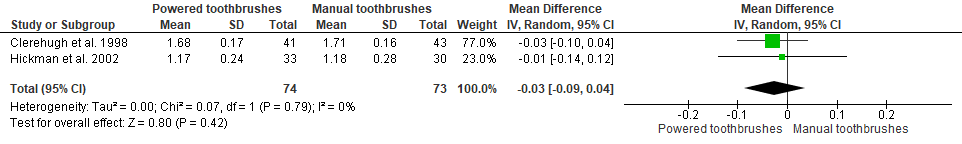


**S6B-29**.Forrest Plots of end-trial scores


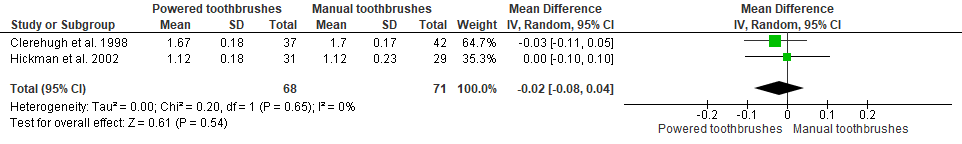


**S6B-30**.Forrest Plots of incremental difference

**
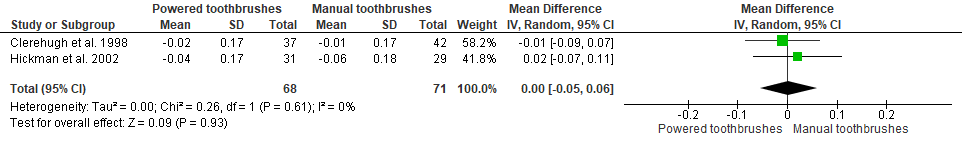
**

●Subgroup meta-analysis on the same index and action model 4: Sonic on GI

**S6B-31**.Forrest Plots at baseline


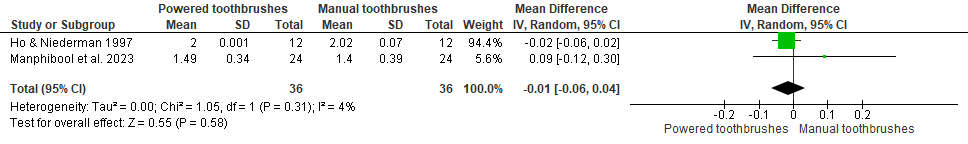


**S6B-32**.Forrest Plots of end-trial scores


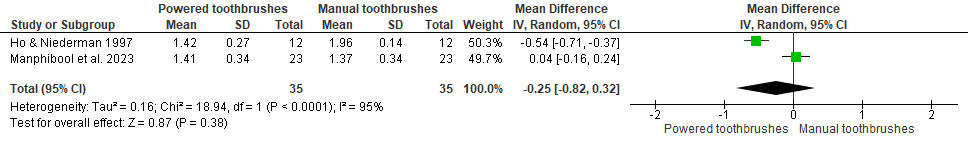


**S6B-33**.Forrest Plots of incremental difference

**
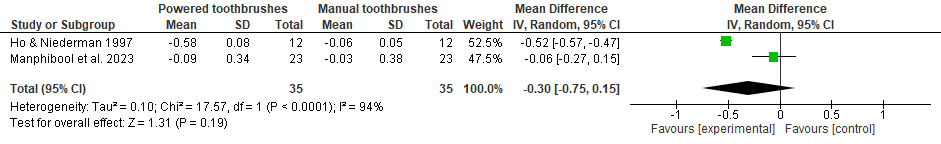
**

**S6C.** Forrest Plots of the overall meta-analysis for **Gingival** **Bleeding scores,** subgroup meta-analyses for the same bleeding index, subgroup meta-analyses for gingival bleeding scores based on the same action mode of powered toothbrushes, and subgroup meta-analyses based on the same bleeding index and action mode of powered toothbrushes after the longest follow up after the longest follow up. SMD or MD are presented for the baseline, end-trial and incremental difference scores using a random-effects model.

●Overall meta-analysis on different indices: Bleeding on probing (BOP), modified papillary bleeding index (MPBI), Eastman interdental bleeding index by Caton & Polson (EIBI, 1985), gingival bleeding index by Ainamo and Bay (BI, 1975), the modified simplified gingival index by Lindhe (MGI-S, 1984)

**S6C-1**.Forrest Plots at baseline


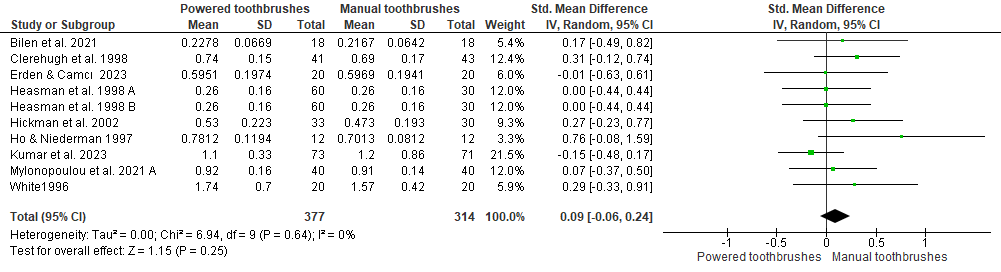


**S6C-2**.Forrest Plots of end-trial scores


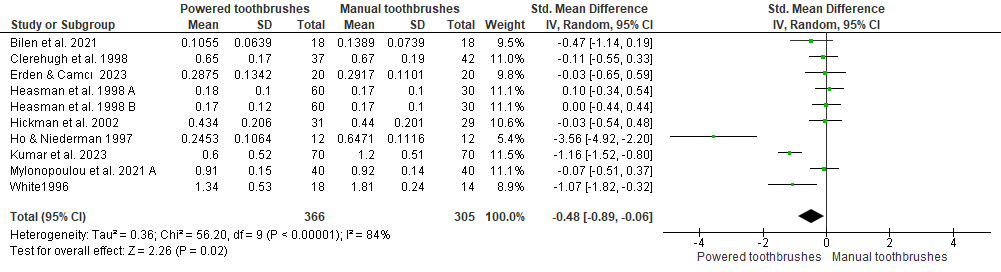


**S6C-3**.Forrest Plots of incremental difference


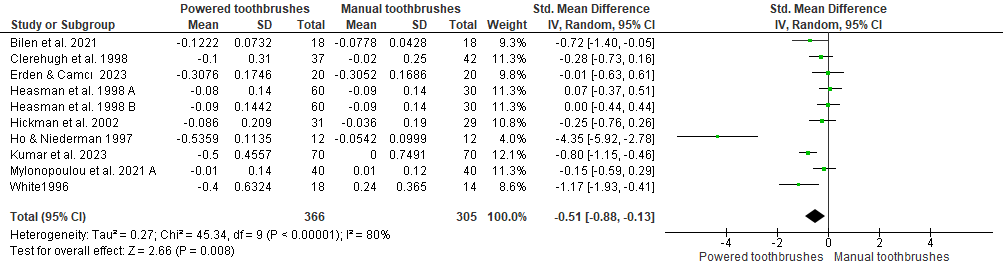


●Subgroup meta-analysis on the same index 1: BOP

**S6C-4**.Forrest Plots at baseline


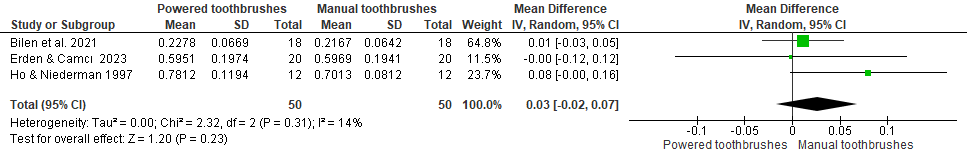


**S6C-5**.Forrest Plots of end-trial scores


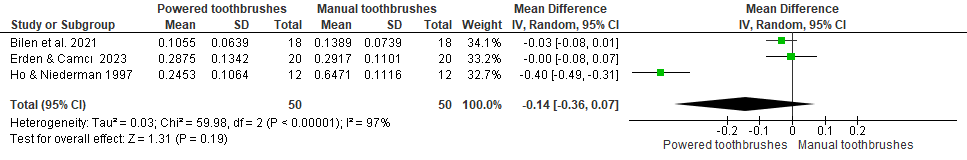


**S6C-6**.Forrest Plots of incremental difference


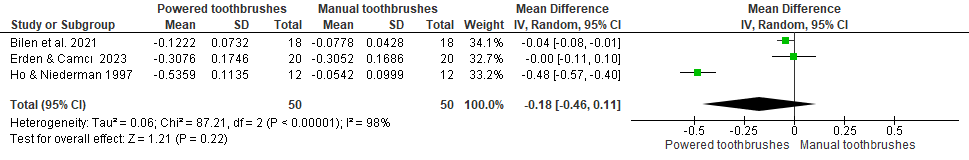


●Subgroup meta-analysis on the same index 2: MPBI

**S6C-7**.Forrest Plots at baseline


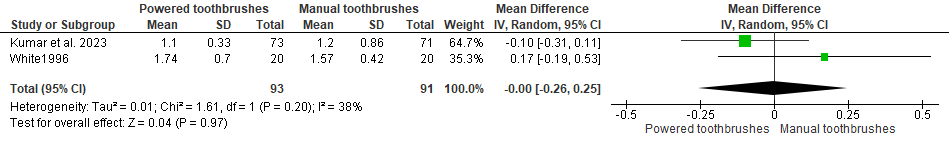


**S6C-8**.Forrest Plots of end-trial scores


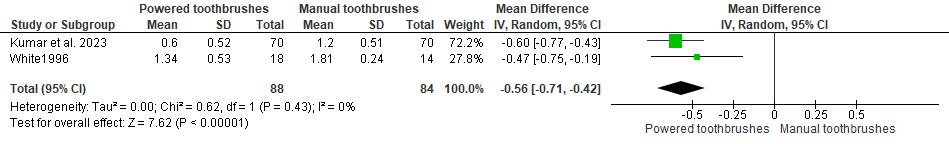


**S6C-9**.Forrest Plots of incremental difference


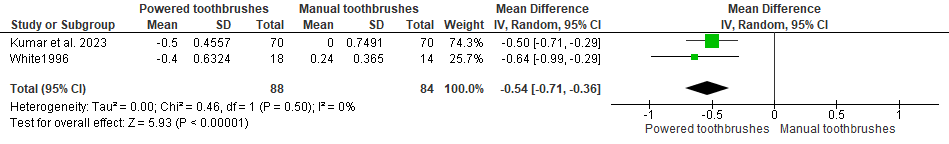


●Subgroup meta-analysis on the same index 3: EIBI

**S6C-10**.Forrest Plots at baseline


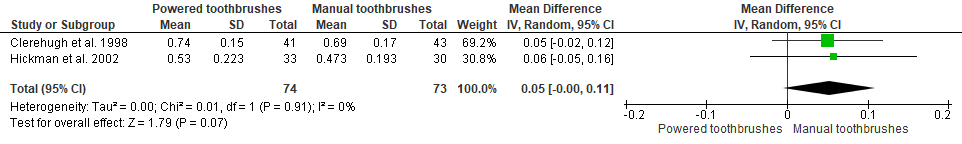


**S6C-11**.Forrest Plots of end-trial scores


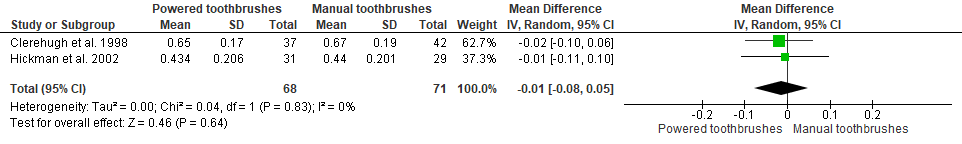


**S6C-12**.Forrest Plots of incremental difference

**
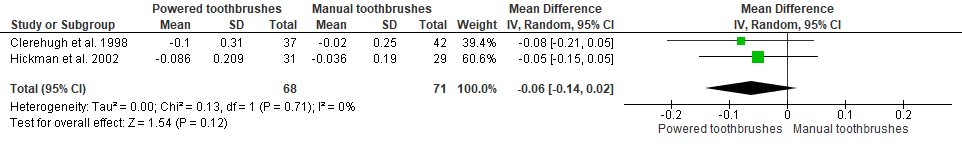
**

●Subgroup meta-analysis on the same index 4: BI

**S6C-13**.Forrest Plots at baseline


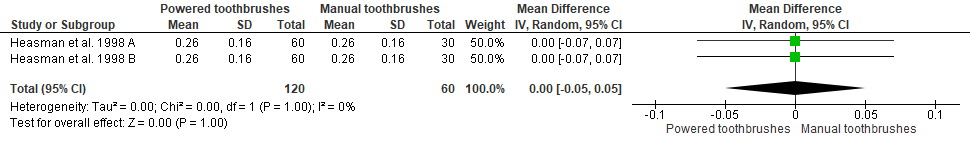


**S6C-14**.Forrest Plots of end-trial scores


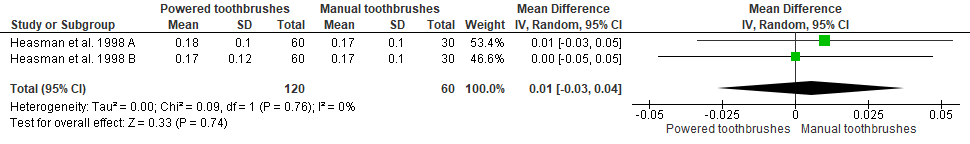


**S6C-15**.Forrest Plots of incremental difference


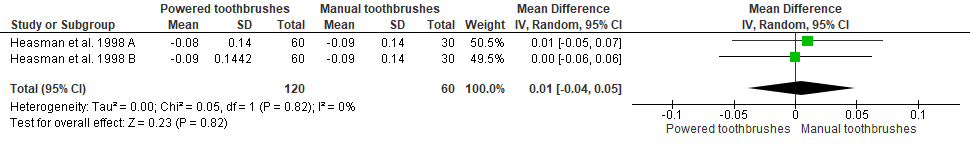


●Subgroup meta-analysis on the same action model 1: ORP/OR

**S6C-16**.Forrest Plots at baseline


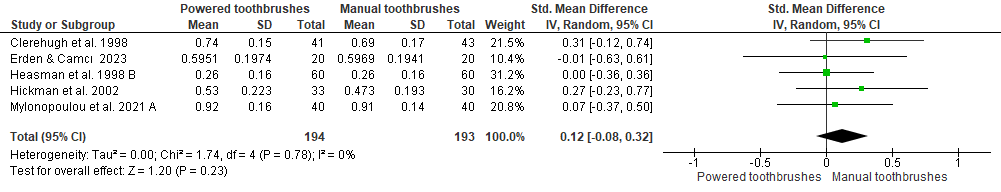


**S6C-17**.Forrest Plots of end-trial scores

**
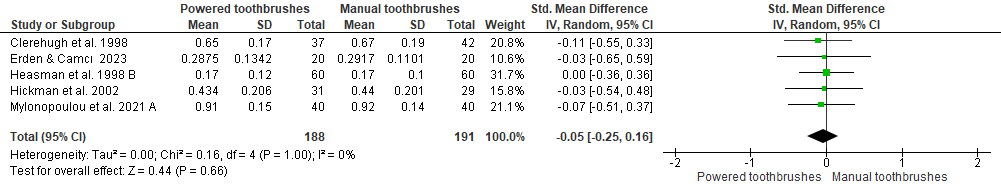
**

**S6C-18**.Forrest Plots of incremental difference

**
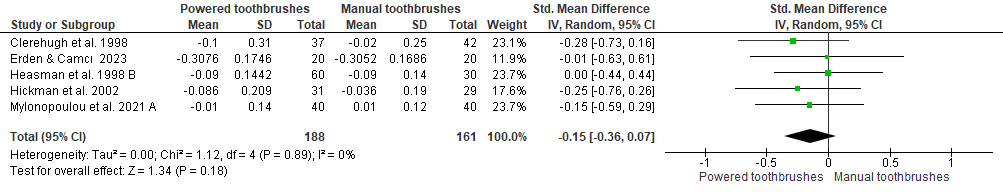
**

●Subgroup meta-analysis on the same action model 2: ORP

**S6C-19**.Forrest Plots at baseline


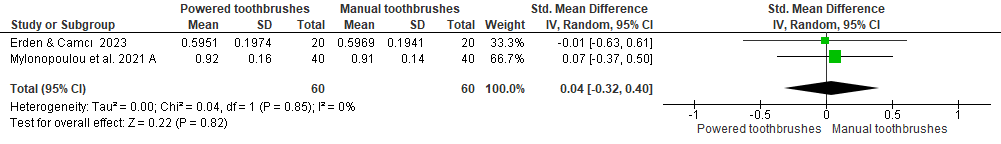


**S6C-20**.Forrest Plots of end-trial scores

**
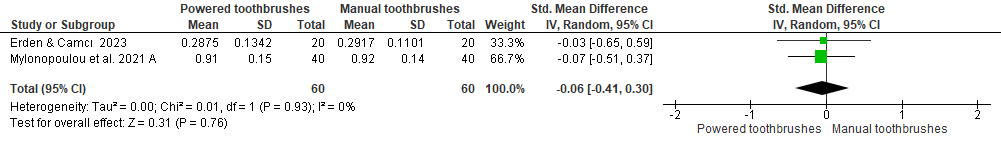
**

**S6C-21**.Forrest Plots of incremental difference

**
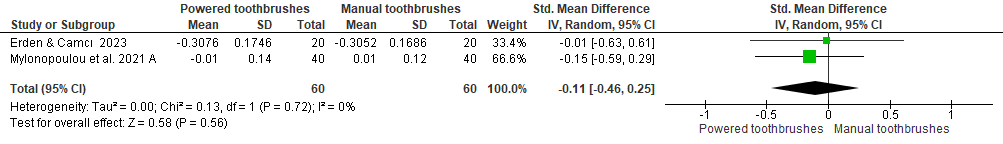
**

●Subgroup meta-analysis on the same action model 3: OR

**S6C-22**.Forrest Plots at baseline


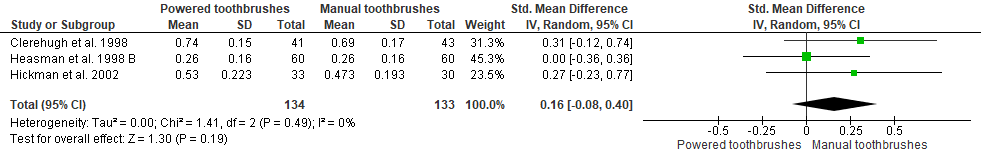


**S6C-23**.Forrest Plots of end-trial scores

**S6C-24**.Forrest Plots of incremental difference

●Subgroup meta-analysis on the same action model 4: Sonic

**S6C-25**.Forrest Plots at baseline

**S6C-26**.Forrest Plots of end-trial scores

**S6C-27**.Forrest Plots of incremental difference

●Subgroup meta-analysis on the same index and action model: OR on EIBI

**S6C-28**.Forrest Plots at baseline

**S6C-29**.Forrest Plots of end-trial scores

**S6C-30**.Forrest Plots of incremental difference

**S7. Sensitivity analyses** of overall meta-analyses with end-trial data.

**S7A. Plaque scores**

**S7A-1.** Leave-One-Out sensitivity analyses ordered by increasing effect estimate.

The above plot is ordered by increasing effect estimate, measured by SMD. The numerically lowest pooled effect estimate occurred after omitting Erbe et al. (2019), whereas the numerically lowest I² heterogeneity occurred after omitting either Erbe et al. (2019) or Kumar et al. (2023).

**S7A-2.** Sensitivity analysis including only studies with low risk of bias.

**S7A-3.** Sensitivity analysis including only studies without industry involvement

**S7A-4.** Sensitivity analysis including only studies designed as RCTs

**S7A-5.** Sensitivity analysis including only studies with low risk of bias and designed as RCTs

**S7A-6.** Sensitivity analysis including only studies without industry involvement and designed as RCTs

**S7B. Gingivitis scores**

**S7B-1.** Leave-One-Out sensitivity analyses ordered by increasing effect estimate.

The above plot is ordered by increasing effect estimate, measured by SMD. The numerically lowest pooled effect estimate and I^2^ heterogeneity occurred when omitting Kumar et al. (2023).

**S7B-2.** Sensitivity analysis including only studies with low risk of bias.

**S7B-3.** Sensitivity analysis including only studies without industry involvement.

**S7B-4.** Sensitivity analysis including only studies designed as RCTs

**S7B-5.** Sensitivity analysis including only studies with low risk of bias and designed as RCTs

**S7B-6.** Sensitivity analysis including only studies without industry involvement and designed as RCTs

**S7C. Gingival Bleeding scores**

**S7C-1.** Leave-One-Out sensitivity analyses ordered by increasing effect estimate.

The above plot is ordered by increasing effect estimate, measured by SMD. The numerically smallest pooled effect estimate and I^2^ heterogeneity occurred when omitting Ho & Niederman (1997).

**S7C-2.** Sensitivity analysis including only studies with low risk of bias.

**S7C-3.** Sensitivity analysis including only studies without industry involvement.

**S7C-4.** Sensitivity analysis including only studies designed as RCTs

**S7C-5.** Sensitivity analysis including only studies with low risk of bias and designed as RCTs

**S7C-6.** Sensitivity analysis including only studies without industry involvement and designed as RCTs

**S8. Publication bias** assessment of end-trial meta-analyses.

**S8A. Plaque scores**

By adding contour lines that indicate conventional milestones in levels of statistical significance (e.g., <0.01, <0.05, <0.1), the contour-enhanced funnel plot above improves interpretation of funnel plot asymmetry [33]. The unshaded (white) region corresponds to no significance, the light gray region to significance between 10% and 5%, the gray region between 5% and 1%, and the dark gray region to 1% significance. This plot could be used naturally in conjunction with the trim-and-fill method, which estimates the likely location of missing studies [33]. Visually, the plot reveals funnel plot asymmetry, with the trim-and-fill method filling 1 missing studies significantly favoring MTBs. After adjusting for bias using the trim-and-fill method, the difference between PTBs and MTBs is not significant (p = 0.07). Egger’s test suggests no publication bias (p = 0.057), while Begg’s test indicates its presence (p = 0.033)[30, 31, 101].

**S8B. Gingivitis scores**

Visually, the plot reveals funnel plot asymmetry; however, the trim-and-fill method did not add any missing studies. Egger’s test suggests no publication bias (p = 0.393), while Begg’s test indicates its presence (p = 0.025) [30, 31, 101].

**S8C. Gingival Bleeding scores**

Visually, the plot reveals funnel plot asymmetry, with the trim-and-fill method filling 1 missing studies significantly favoring MTBs. After adjusting for bias using the trim-and-fill method, the difference between PTBs and MTBs is not significant (p = 0.125). However, Egger’s and Begg’s tests support the absence of publication bias (p = 0.084 and p = 0.089, respectively)[30, 31, 101].

**S9. Trial Sequential Analysis (TSA)** results**.**

**S9A. Plaque scores**

**S9A-1.** TSA of incremental-difference plaque scores using orthodontic modification of the Silness & Löe plaque index by Williams et al. (OMPI, 1991)[84] to compare powered and manual toothbrushes.

The accumulated number of participants did not reach the required information size and the cumulative Z-curve did not cross any monitoring boundary.

**S9A-2.** TSA of incremental-difference plaque scores using Quigley-Hein plaque index [91], its modification and the Turesky-modified Quigley–Hein index [90] to compare oscillating-rotating-pulsating powered and manual toothbrushes.

The accumulated number of participants did not reach the required information size and the cumulative Z-curve did not finally cross any monitoring boundary.

**S9A-3.** TSA of incremental-difference plaque scores using O’Leary plaque index (1972)[89] and its modification to compare powered and manual toothbrushes.

The accumulated number of participants did not reach the required information size and the cumulative Z-curve did not finally cross any monitoring boundary.

**S9A-4.** TSA of incremental-difference plaque scores using Silness & Löe plaque index [85] to compare powered and manual toothbrushes.

The accumulated number of participants did not reach the required information size and the cumulative Z-curve did not finally cross any monitoring boundary.

**S9A-5.** TSA of incremental-difference plaque scores using visible plaque index by Ainamo & Bay [88] to compare powered and manual toothbrushes.

The accumulated number of participants did not reach the required information size and the cumulative Z-curve did not cross the O’Brian-Fleming boundaries.

**S9B. Gingivitis scores**

**S9B-1.** TSA of incremental-difference gingivitis scores using Löe & Silness gingival index [86] to compare powered and manual toothbrushes.

The accumulated number of participants did not reach the required information size and the cumulative Z-curve did not cross any monitoring boundary.

**S9B-2.** TSA of incremental-difference gingivitis scores using Löe & Silness gingival index [86] to compare sonic powered and manual toothbrushes.

The accumulated number of participants did not reach the required information size and the cumulative Z-curve did not finally cross any monitoring boundary.

**S9C. Gingival Bleeding scores**

**S9C-1.** TSA of incremental-difference gingival bleeding scores using bleeding on probing index to compare powered and manual toothbrushes.

The accumulated number of participants did not reach the required information size and the cumulative Z-curve did not finally cross any monitoring boundary.

**S9C-2.** TSA of incremental-difference gingival bleeding scores using modified papillary bleeding index [102] to compare powered and manual toothbrushes.

The accumulated number of participants reached the required information size and the cumulative Z-curve crossed the O’Brian-Fleming boundaries.

**S9C-3.** TSA of incremental-difference gingival bleeding scores using Eastman interdental bleeding index by Caton & Polson [103] to compare oscillating-rotating powered and manual toothbrushes.

The accumulated number of participants did not reach the required information size and the cumulative Z-curve did not cross any monitoring boundary.

**S10A.** Estimated evidence profile and the strength of recommendations on the efficacy of PTBs with different action modes vs. MTBs for reducing **Plaque scores** in orthodontic patients with fixed appliances.

| **Determinants of the Quality** | **ORP/OR** | **ORP** | **OR** | **CR** | **Sonic** |
| --- | --- | --- | --- | --- | --- |
| Study design | RCT | RCT | RCT | RCT/CCT | RCT/CCT |
| # studies (Figure 1, Table 2)  # comparisons (Figure 1, Table 2)  # meta-analysis (Figure 1, Table 3) | 10  18  6 | 6  10  3 | 5  8  3 | 5  7  2 | 6  6  4 |
| Risk of bias (Supplementary S2, S4) | Low to high | Low to high | Low to high | Moderate to high | Moderate to high |
| Consistency | Inconsistent | Inconsistent | Rather consistent | Rather inconsistent | Inconsistent |
| Directness | Direct | Direct | Direct | Direct | Direct |
| Precision | Precise | Imprecise | Precise | Imprecise | Rather precise |
| Publication bias | Possible | Possible | Possible | Possible | Possible |
| The quality of a body of evidence | Low | Very low | Moderate | Very low | Low |
| Magnitudes of the effect size | None | None | None | Large | None |

PTB: powered toothbrush; MTB: manual toothbrush; RCT: randomized controlled clinical trial; CCT: controlled clinical trial; ORP: oscillating-rotating-pulsating action; OR: oscillating-rotating action; CR: counter-rotational action

**S10B.** Estimated evidence profile and the strength of recommendations on the efficacy of PTBs with different action modes vs. MTBs for reducing **Gingivitis scores** in orthodontic patients with fixed appliances.

| **Determinants of the Quality** | **ORP/OR** | **ORP** | **OR** | **CR** | **Sonic** |
| --- | --- | --- | --- | --- | --- |
| Study design | RCT | RCT | RCT | RCT/CCT | RCT/CCT |
| # studies (Figure 1, Table 2)  # comparisons (Figure 1, Table 2)  # meta-analysis (Figure 1, Table 3) | 6  9  4 | 3  4  2 | 4  5  2 | 3  3  2 | 3  3  2 |
| Risk of bias (Supplementary S2, S4) | Low to high | Low to high | Low to high | Moderate to high | Moderate to high |
| Consistency | Consistent | Consistent | Consistent | Rather inconsistent | Inconsistent |
| Directness | Direct | Direct | Direct | Direct | Direct |
| Precision | Rather precise | Imprecise | Rather imprecise | Imprecise | Imprecise |
| Publication bias | Possible | Possible | Possible | Possible | Possible |
| The quality of a body of evidence | Moderate | Low | Low | Very low | Very low |
| Magnitudes of the effect size | None | None | None | None | None |

PTB: powered toothbrush; MTB: manual toothbrush; RCT: randomized controlled clinical trial; CCT: controlled clinical trial; ORP: oscillating-rotating-pulsating action; OR: oscillating-rotating action; CR: counter-rotational action

**S10C.** Estimated evidence profile and the strength of recommendations on the efficacy of PTBs with different action modes vs. MTBs for reducing **Gingival** **Bleeding scores** in orthodontic patients with fixed appliances.

| **Determinants of the Quality** | **ORP/OR** | **ORP** | **OR** | **CR** | **Sonic** |
| --- | --- | --- | --- | --- | --- |
| Study design | RCT | RCT | RCT | RCT | RCT/CCT |
| # studies (Figure 1, Table 2)  # comparisons (Figure 1, Table 2)  # meta-analysis (Figure 1, Table 3) | 9  12  5 | 5  6  2 | 5  6  3 | 2  2  0 | 5  5  3 |
| Risk of bias (SupplementaryS2, S4) | Low to high | Low to high | Low to high | Moderate to high | Moderate to high |
| Consistency | Consistent | Consistent | Consistent | Consistent | Inconsistent |
| Directness | Direct | Direct | Direct | Direct | Direct |
| Precision | Precise | Imprecise | Precise | Imprecise | Rather precise |
| Publication bias | Possible | Possible | Possible | Possible | Possible |
| The quality of a body of evidence | Moderate | Low | Moderate | Low | Low |
| Magnitudes of the effect size | None | None | None | Not available | None |

PTB: powered toothbrush; MTB: manual toothbrush; RCT: randomized controlled clinical trial; CCT: controlled clinical trial; ORP: oscillating-rotating-pulsating action; OR: oscillating-rotating action; CR: counter-rotational action

**S11.** A descriptive presentation of Supplementary S2-3 and other relevant information.

*Study design*

Of the 23 included papers[38-60], 19 were RCTs and the other 4 were CCTs[43, 56, 58, 59]. Blinding procedures were unspecified in 4 papers[43, 46, 47, 56], and the remaining 19 were single-blinded, with 5 explicitly reporting the assessor was blinded to group allocation[41, 44, 45, 48, 57]. Additionally, 13 papers used a parallel-group design, whereas the other 10 employed a crossover design[40, 48, 49, 51, 53, 54, 57-60]. The washout periods in these crossover studies varied: from approximately 1 month in 3 papers[40, 51, 54], 2 weeks in 2[48, 49], to no washout in 5[53, 57-60].

*Intervention duration*

Of the 23 included papers, intervention durations varied: from 1 week in 1 paper[60], 2 weeks in 2[45, 57], approximate 1 month in 8[40, 48, 49, 51, 53, 55, 58, 59], 6 weeks in 1[44], approximate 2 months in 7[39, 43, 46, 47, 50, 52, 54], approximate 3 months in 3[38, 41, 56], to 4 months in 1[42].

*Oral prophylaxis*

Among the 23 included papers, 10 did not specify oral prophylaxis details; 6 reported no oral prophylaxis[39, 44, 46, 47, 50, 52], and the remaining 7, which were crossover studies, indicated participants received oral prophylaxis[51, 53, 54, 57-60]. Thienpont et al. provided scaling and polishing after each scoring[51]. Heasman et al. administered oral prophylaxis before crossing-over toothbrush types[53]. In four other studies, participants underwent oral prophylaxis before using each toothbrush type[54, 57, 58, 60]. In the study by Jackson, participants received scaling only after the first baseline scoring[59].

*Other Inclusion and exclusion criteria of the included papers*

In addition to required fixed appliances and oral prophylaxis, other inclusion criteria across the included papers were as follows: good general health[41, 44, 47, 50-52, 54, 57, 60], right-handed[38, 42], daily MTB use[38, 44], non-extraction orthodontic treatment[41, 42, 49], uniform bracket type and ligation technique[42, 44], moderate plaque-induced gingivitis[41, 55], at least 16 natural teeth (excluding third molars) with facial and lingual scorable surfaces[44] or at least 20 teeth[40], familiarity with smartphone use[44], gingival bleeding on at least 20% or 30% of sites[41, 47, 50, 52], and good compliance or willingness to participate[47, 50, 52, 57, 58, 60].

The exclusion criteria across the included studies were as follows: physical or mental disorders[38, 40-42, 47, 50, 52, 53, 55], systemic disease[38-42, 48, 60], craniofacial deformities[41], pregnancy[45], debilitating disease[52, 58], regular drug use[38, 42, 45], smoking or other tobacco use[38, 40, 41, 44, 48], poor [38, 47, 49-52], tooth agenesis[41], missing teeth[42], dental protheses or implants[41], perioral or intraoral piercing[41], additional materials (chains, figure-eight ligatures or coil springs)[38], lingual orthodontic appliances[39], rotations or a diastema in arches[38], restorations affecting labial surfaces[38], more than 2 cervical or proximal fillings[41], local predisposing factors (i.e. composite remnant)[45], active carie[41, 44, 47, 50, 52, 57], periodontitis or severe periodontal problems[40-42, 44, 47, 48, 50, 52, 57, 60], periodontal disease history[45, 53], severe gingivitis[44], taking antibacterial agents or medication influencing gingival health/plaque[38-41, 44, 45, 47-50, 52, 55-58, 60], additional plaque-eliminating tools (flossing or interdental brushes)[39], other chemotherapeutics[56], other antiseptic mouthwash (MW) such as chlorhexidine (CHX)[44, 47, 50, 52, 57], oral hygiene instruction within 6 months[53], experience with orthodontic MTBs (O-MTBs)[53], previous or current PTB use[41, 42, 47, 50, 53], and participation in other trials[41]. No additional specific inclusion and exclusion criteria were reported in the remaining 3 studies[43, 46, 59].

*Toothbrush Types*

The 23 included papers examined PTBs with various action modes and brush head designs, as well as MTBs with different brush head designs. PTBs incorporated at least 6 different action modes: 3D (oscillating-rotating-pulsating, ORP) in 6 papers[38, 41, 44, 46, 48, 51], oscillating-rotating (OR) in 5[45, 50-53], counter-rotational (CR) in 5[42, 54, 57, 58, 60], rotary in 1[54], and sonic in 6[40, 43, 49, 53, 55, 56]. Despite this, some PTB action modes were unspecified in 2 mentioned papers[43, 54] and the remaining 3 did not report any PTB action modes[39, 47, 59]. Regarding PTB brush heads, regular brush heads (RBH) were used in 2 papers[38, 44], orthodontic brush heads (OBH) in 4[41, 46, 50, 52], both RBH and OBH in 2[51, 53], and CrossAction brush head (CBH) in 1[42]. Among MTBs, 10 papers examined O-MTBs[38, 40-43, 45, 46, 51, 53, 58], while 3 did not provide MTB details[39, 47, 59].

*Brushing regimen*

Brushing regimens varied considerably among the included papers. In 3 papers, both PTB and MTB groups used the Bass technique or its modification[40, 45, 56]. In 14 papers, PTB users followed the manufacturers' instructions, with one specifying the 'daily clean' mode[38]. For the MTB users of the same 14 papers, 10 studies recommended the Bass technique or its modification; Silvestrini Biavati et al. advised the rotation/vibration technique[46]; Heasman et al. combined the Bass and Charters techniques[53]; and the remaining 2 studies indicated that participants followed their own customary manner[44] or orthodontic patient instructions[59], without further details. The reported brushing duration ranged from 1 to 4 minutes, with a frequency of at least twice daily.

*Toothpaste, other oral hygiene products or tools, compliance strategy*

Of the 23 included papers, 16 reported that participants in both PTB and MTB groups were provided with the same toothpaste (TP), with 6 specifying fluoride TP[46, 47, 50, 52-54] and 1 specifying non-fluoride TP[47]. Three studies indicated participants in both groups used the same fluoride mouthwash (MW)[47, 50, 52]. Trombeli et al. specified that chlorhexidine (CHX) TP and MW were not permitted[57]. No other oral hygiene products were used in 13 papers [38, 41, 42, 44, 46, 48, 51, 52, 54, 56-59].

In 4 studies, participant cooperation was reinforced through various methods: 1 employed a compliance worksheet and routine SMS notifications[39], 2 used a toothbrushing diary[50, 52], and 1 required parental reinforcement at home[55].

*Clinical indices for evaluation*

Significant variability in the three parameters of interest was noted among the included papers, as summarized in Supplementary S2.

The following clinical indices were used to assess PS:

- Silness & Löe plaque index (PI, 1964)[85] in 6 papers[39, 40, 43, 47, 55, 60];
- Quigley-Hein plaque index (QHPI, 1962)[91] and its modification[49] in 5 papers[38, 44, 49, 51, 54];
- Turesky-modified Quigley–Hein index (TMQH, 1970) [90] in 2 papers[38, 44];
- Orthodontic modification of the Silness & Löe plaque index by Williams et al. (OMPI, 1991)[84] in 6 papers[41, 42, 48, 50-52];
- O’Leary plaque index (1972)[89] and its modification in 5 papers[41, 45, 46, 57, 58];
- Visible plaque index by Ainamo & Bay (VPI, 1975)[88] in 1 paper[53];
- Plaque index brackets (BPI) in 1 paper[54];
- Hygiene analysis index (HAI) in 1 paper[56];
- Orthodontic plaque index by Jackson (JOPI, 1991) in 1 paper[59].

The following clinical indices were used to assess GS:

- Löe & Silness Gingival index (GI, 1963,1964,1967)[85-87] and its modifications in 11 papers[38-43, 45, 50, 52, 55, 59];
- Lobene modified gingival index (LMGI, 1986)[92] in 2 papers[51, 54];
- Gingival index by Ainamo et al. (AGI, 1982)[104] in 1 paper[47];
- Ramfjord gingival index (RGI, 1959)[93] in 1 paper[58].

The following clinical indices were used to assess BS:

- Bleeding on probing (BOP) in 6 papers[38, 42, 45, 51, 54, 55];
- Modified papillary bleeding index (MPBI, 1980)[102] in 2 papers[39, 56];
- Modified simplified gingival index by Lindhe (MGI-S, 1984)[105] in 1 paper[41];
- Eastman interdental bleeding index (EIBI,1985)[103] in 4 papers[43, 47, 50, 52];
- Ainamo & Bay gingival bleeding index (BI, 1975)[88] in 2 papers[46, 53];
- Papillary bleeding index by Mühlemann (PBI, 1977)[106] in 1 paper[49];
- Löe & Silness gingival/plaque index for bleeding (LSBI, 1964)[85] in 1 paper[48].

***References continue from the main manuscript:***

101. Sterne JA, Sutton AJ, Ioannidis JP, et al. Recommendations for examining and interpreting funnel plot asymmetry in meta-analyses of randomised controlled trials. *BMJ*. 2011;343:d4002. <https://doi.org/10.1136/bmj.d4002>

102. Barnett ML, Ciancio SG, Mather ML. The modified papillary bleeding index: Comparison with gingival index during the resolution of gingivitis. *The Journal of Preventive Dentistry*. 1980;6:135-38.

103. Caton JG, Polson AM. The interdental bleeding index: a simplified procedure for monitoring gingival health. Compend Contin Educ Dent (Lawrenceville). 1985;6(2):88, 90-2.

104. Ainamo J, Barmes D, Beagrie G, et al. Development of the World Health Organization (WHO) community periodontal index of treatment needs (CPITN). *Int Dent J*. 1982;32(3):281-91.

105. Lindhe J. Textbook of Clinical Periodontology. Copenhagen，Danmark: Munskgaard International; 1984.

106. Muhlemann HR. Psychological and chemical mediators of gingival health. *J Prev Dent*. 1977;4(4):6-17.
